# Novel multigene molecular characterization of avian reovirus strains and associated embryonic pathogenicity

**DOI:** 10.1128/jvi.01982-25

**Published:** 2026-05-05

**Authors:** Islam Nour, Julia R. Blakey, Sonsiray Alvarez-Narvaez, Arun Kulkarni, Quentin D. Read, Sujit K. Mohanty

**Affiliations:** 1 United States Department of Agriculture, Agricultural Research Service (USDA-ARS), Southeast Poultry Research Laboratories, US National Poultry Research Center, Athens, Georgia, USA; 2 Georgia Poultry Laboratory Network, Gainesville, Georgia, USA; 3 US Department of Agriculture, Agricultural Research Service, Office of the Southeast Area Director 503363 , Raleigh, North Carolina, USA; Emory University School of Medicine, Atlanta, Georgia, USA

**Keywords:** avian reovirus, genotyping, tenosynovitis, embryonic pathogenicity, phylogenetic analysis, antigenic epitopes

## Abstract

Avian reovirus (ARV) infection causes a variety of health problems in poultry, including tenosynovitis/arthritis, hepatitis, malabsorption, etc., leading to huge economic losses. Therefore, regular monitoring of ARV strains is crucial for detecting new variants to mitigate clinical disease. Traditional classification approaches have focused primarily on a single gene, σC, which encodes the viral spike protein that elicits neutralizing antibodies. However, additional capsid proteins could play a role by inducing a host antiviral immune response. In this study, the λC, μB, σC, and σB proteins encoded by the L3, M2, S1, and S3 genomic segments, respectively, were analyzed to classify clinical ARV strains with respect to embryonic pathogenicity and genotype–phenotype relationships. Embryo inoculation revealed strain-dependent pathogenicity ranging from the absence of tendon lesions (strain 23-272) to multifocal ulceration with fibrinoheterophilic crusting (22-806) and multifocal necrotizing hepatitis (23-087). Pairwise log-rank and Cox regression tests demonstrated highly significant strain-dependent differences in survival, defining a gradient from rapid embryonic lethality to delayed mortality, with one low-virulence strain exhibiting partial survival. While the λB-σB dual genotyping approach showed insignificant concordance with virulence clusters, a secondary structure-based μB-λC-σC triple model emerged as a better predictor of virulence. Notably, σC exhibited substantial variability in antigenic epitopes, whereas σB remained relatively conserved. These results might explain reduced vaccine efficacy against recent variants and support shifting vaccine design from σC to σB antigen or a combination to achieve broader protection. In conclusion, this multigene, structure-informed framework enhances ARV classification, links genotypes with virulence, and informs improved vaccination strategies.

**IMPORTANCE:**

Avian reovirus (ARV) represents a major threat to poultry health and production, primarily through its association with tenosynovitis/arthritis and the emergence of vaccine-resistant strains driven by high genetic diversity. Effective control requires an ARV classification system beyond traditional schemes based solely on the σC protein, which have proven insufficient to capture pathogenic diversity. We propose a novel, structure-informed triple genotyping approach incorporating the major capsid (μB), the turret (λC), and the virus attachment (σC) proteins. This method improves the prediction of disease severity in chicken embryos while reducing reliance on extensive animal challenge models. Furthermore, our protein-based analysis highlights that the conserved σB protein or a combination of σC & σB protein might be a promising vaccine target capable of providing broader protection across diverse ARV strains, thereby offering new avenues for both improved strain classification and rational vaccine design.

## INTRODUCTION

Avian reovirus (ARV) is a pathogen of domestic poultry as well as of wild birds. Infection leads to a wide spectrum of clinical presentations, including tenosynovitis/arthritis, enteric disease, hepatitis, myocarditis, malabsorption, runting–stunting syndrome, and immunosuppression (1–4). While ARV infections often result in subclinical disease, the associated pathological consequences can lead to diminished productivity parameters such as lower weight gain, reduced flock uniformity, and decreased feed conversion rates. In addition to animal welfare concerns, ARV infections increase the risk of lameness-related condemnations during processing, resulting in substantial economic losses (5, 6). Infection with ARV can result in high morbidity (20%–40%) and mortality (up to 10%) (7). ARV was identified as a top 10 research priority in the broiler industry and the number one priority in turkey production according to the 2022 American Association of Avian Pathologists (AAAP) Research Priorities survey (8). Recent data from the United States Animal Health Association (USAHA) underscores this, with ARV-related issues ranking among the top 10 challenges in 2023 (9). A particularly devastating ARV outbreak occurred in California in 2015, characterized by exceptionally high morbidity and mortality rates (10). Despite decades of routine vaccination with traditional strains, the emergence of vaccine-resistant ARV isolates has been identified as a key factor contributing to this outbreak (11).

ARV is a non-enveloped virus with a segmented double-stranded (ds) RNA genome (12). The 10 segments comprising the genome are divided into three classes based on their electrophoretic mobility: L-class (L1–L3), M-class (M1–M3), and S-class (S1–S4) (13, 14). The high mutation rate and recombination potential characteristic of RNA viruses, coupled with selective pressure exerted by the traditional live-attenuated vaccines, has most probably contributed to the development of immune escape ARV variants and the breakdown of vaccine efficacy (10, 15). Therefore, comprehensive characterization of circulating ARV strains is essential for developing effective control measures. We recently reported arthritis/tenosynovitis-causing ARV strains belonging to genocluster (GC) VI in North Carolina (16). The emergence of these strains might be linked to the use of autogenous vaccines containing a single GC V variant and two GC I isolates in breeder flocks, potentially favoring the fitness of GC VI strains over others (10, 17).

Considerable attention has been focused on the S1 segment, which encodes the viral attachment and major immunogenic protein σC. Although the σC gene has been commonly used to classify ARV strains, other viral genes have been found to contribute significantly to the genetic diversity of the virus (18). So far, several genogroup classification approaches have been reported based on σC (7, 19–22), but single-gene analysis is no longer sufficient to accurately define the genotype and pathotype of an ARV strain (11). The vaccine genogroup (G I) was reported to contain ARV strains belonging to various pathotypes with different organ/tissue tropisms in the same genogroup (23–25). Moreover, other viral genes add high variability to the ARV genome (10). The µB and λC genes, for instance, show significant genetic divergence and have been suggested to be used together along with σC to enhance reovirus classification (18). The S3-encoded σB protein is a major outer capsid component that forms a stable tertiary complex with μB and μBC required for efficient virion assembly, and it harbors group-specific neutralizing epitopes (26, 27). Therefore, we hypothesized that a multi-gene-based analysis could be better than only σC-based genotyping and would allow the correlation between viral genome and virulence. In the current study, we tested our hypothesis on clinical strains with well-characterized clinical signs to develop a more accurate ARV genomic classification that would correlate with virulence. Moreover, we investigated whether the viral-associated pathogenesis and severity observed in birds could be replicated in chicken embryos.

## MATERIALS AND METHODS

### Clinical samples

ARV field strains (*n* = 15) were collected from diseased commercial broiler flocks located in Georgia (22-460, 22-806, 22-835, 23-272, 23-087, and 22-861), Iowa (ARV_126484, ARV_ 106761, and ARV_127720), Texas (ARV_115940), Alabama (ARV_94584 and ARV_141045), and Arkansas (ARV_122301 and ARV_126695). Tendon samples (*n* = 14) and a liver sample (strain 23-087) were collected from birds presenting the symptoms of ARV infection and were confirmed by RT-qPCR to be exclusively infected with ARV for virus isolation and propagation. ARV isolates originating from Iowa, Texas, Alabama (ARV_94584 and ARV_141045), and Arkansas were provided by Dr. Sellers at the Poultry Diagnostic Research Center, Athens, GA, US. The Alabama isolate was obtained from Dr. Conrad’s repository at the US National Poultry Research Center (USNPRC, Athens, GA, USA). All experiments were approved by the Institutional Biosafety Committee (Registration No. 2023-17).

### Virus isolation and propagation

Tendons and liver tissues obtained from broiler chickens exhibiting arthritis or tenosynovitis were minced with sterile scissors in a plastic container in Dulbecco’s modified Eagle medium (DMEM, Fisher Scientific, USA). The mixture was then mechanically disrupted and homogenized for 10 min. The tissue homogenate was transferred to a 15 mL sterile polypropylene tube and centrifuged at 12,000 rpm for 10 min at 4°C. The supernatant was filtered through a 0.22-μm filter (Corning, USA), further diluted with DMEM, and added to freshly cultured QM5 (quail fibrosarcoma) cells. Subsequently, the cells were monitored for the development of reovirus-specific cytopathic effects (CPE) over a 5-day period. Following the formation of virus-induced syncytia and complete destruction of the cell monolayer, three freeze-thaw cycles were performed. The entire cell lysate was centrifuged for 30 min at 5,000 rpm at 4°C in the same centrifuge for removal of cellular debris, followed by ultracentrifugation for 2 h at 36,000 × *g* and 4°C. Subsequently, the resulting pellet was resuspended in 1 mL of DMEM and stored at −80°C for future use.

### Virus titration

QM5 cells were seeded in 24-well CellBIND plates (Corning) at 80% confluency and incubated overnight at 37 °C with 5% CO₂. Ten-fold serial dilutions of virus (10⁻¹–10⁻^7^) were prepared in DMEM, and 200 µL was inoculated onto confluent monolayers. After 1 h adsorption at 37 °C, inocula were removed and replaced with 500 µL overlay medium consisting of a 1:1 mixture of 2% hydroxypropyl methylcellulose (Sigma, USA) and maintenance medium (DMEM with 10% FBS and 1% penicillin/streptomycin). Cells were incubated for 5 days with overlay replenished as needed. Plaques were visualized by fixation/staining with 1% crystal violet, 10% formaldehyde, and 5% ethanol for 2 h, rinsed with water, and air-dried. All plaque assays were performed in triplicate.

Viral titers were calculated as plaque-forming units per milliliter (PFU/mL) using the following formula:

$PFU/\;mL=\frac{Number\;of\;plaques\;}{Dilution\;factor\;\times \;Volume\;of\;inoculum\;\left(mL\right)}$

### Embryo pathogenesis analysis

Fertilized eggs (*n* = 126), obtained from a specific pathogen-free (SPF) flock (maintained in-house at the US National Poultry Research Center [USNPRC]) and routinely screened for ARV, were incubated at 37°C for 10 days. Embryo viability was assessed by candling, with the locations of the embryo and air sac marked. Titrated virus stocks, diluted to a concentration of 10^3^ PFU per 0.1 mL in DMEM, were inoculated onto the dropped chorioallantoic membrane (CAM) of the embryos (seven eggs/strain) using a 23-guage needle. Control samples received 0.1 mL of DMEM only. Eggshell puncture sites were sealed with glue (Elmer’s Nontoxic), and the embryonated eggs were returned to the incubator for continued development. Daily candling allowed for monitoring of embryo viability, with a lack of embryo movement and loss of blood vessels in the shell membranes serving as indicators of non-viability. Mortality occurring within 24 h post-inoculation was considered non-specific and discarded. Mortality was recorded for 7 days post-inoculation, and surviving embryos were assessed for gross lesions at 7 days post-inoculation.

### Histopathological examination

Tissue samples obtained from the tendons, synovial area, hearts, liver, bursa of Fabricius, and other organs of sick chicks exhibiting notable gross lesions were processed at Georgia Poultry Laboratory Network (GPLN, Gainesville, GA, USA) facilities, while the hearts, livers, and limbs of chicken embryos were immediately immersed in 10% neutral-buffered formalin (Sigma Aldrich) for 24–48 h and then stored in 70% ethanol (Fisher Scientific, USA) before being subsequently submitted to the veterinary histopathology lab at the University of Georgia (UGA, Athens, GA, USA). The tissues underwent a series of routine histological processing steps, including dehydration, clearance, embedding in paraffin wax for support, and casting into blocks. The paraffin blocks were then sectioned into 4-μm thick slices using a microtome. These sections were mounted on glass slides and stained with hematoxylin and eosin (H&E).

### Immunohistochemistry for ARV detection

Formalin-fixed, paraffin-embedded (FFPE) sections (4 µm) of chicken embryo liver, heart, and limb tissues were subjected to immunohistochemistry (IHC) for the detection of ARV antigens. Following deparaffinization and graded rehydration, antigen retrieval was performed using citrate buffer (pH 6.0) (Vector Laboratories). Immunohistochemical processing included heat-induced antigen retrieval and detergent-based permeabilization, which are known to increase apparent intercellular spacing in fragile embryonic tissues when compared with conventional H&E staining. Endogenous peroxidase activity was quenched with 3% hydrogen peroxide (Thermo Scientific Chemicals), and nonspecific binding was blocked using 5% goat serum (Gibco). Sections were then incubated overnight at 4°C with chicken-derived anti-ARV polyclonal antibody (AVS Bio). A goat anti-chicken IgG secondary antibody conjugated to horseradish peroxidase (Invitrogen) was applied for 1 h at room temperature. Visualization was achieved using 3,3′-diaminobenzidine (Pierce DAB Substrate Kit, Thermo Scientific) as the chromogen, producing a brown precipitate at sites of antigen localization, which was compared with corresponding H&E-stained sections to assess colocalization with characteristic lesions (28, 29).

### Reverse transcription and full-length genomic amplification

Total RNA was extracted from all viral isolates (*n* = 15) using the Qiagen viral mini-RNA isolation kit (Qiagen), following the manufacturer’s protocol. Reverse transcription of total RNA to cDNA was conducted in a 20 μL reaction volume containing 200 ng of random hexamers, 1 μL of 10 mM dNTP mix, 5 μL of 5X buffer, 1 μL of 0.1 M DTT, and 1 μL of Superscript III reverse transcriptase (Invitrogen). The reaction mixture was incubated under the following conditions: 25°C for 5 min, 50°C for 60 min, and 70°C for 15 min for heat inactivation. Subsequently, the reaction mixture was treated with 1 μL of RNase H (Promega) for 20 min at 37 °C. To amplify the entire genome of each viral segment, PCR was performed using ARV-specific tagged primers (16). A 25 μL PCR mixture was prepared using 2 μL of cDNA template, 500 nM of each forward and reverse primer, 300 μM dNTPs, 5 μL of 5× LongAmp Taq Reaction Buffer, and 1 μL (2.5 U) of LongAmp Taq DNA Polymerase (New England Biolabs). The PCR cycling conditions were as follows: 94°C for 3 min, followed by 35 cycles of 94°C for 30 s and 65°C for 4 min 30 s, with a final extension at 65°C for 10 min. The amplified products were visualized on a 1% agarose gel. The PCR products of the ARV’s genomic segments were purified using the MinElute PCR purification kit (Qiagen).

### ARV targeted-genomic segment sequencing and raw-data processing

The Qubit dsDNA HS assay kit (Invitrogen) was employed to accurately determine the concentration of each purified segment. Subsequently, Oxford Nanopore Technologies’ long-read sequencing was performed on 300 ng of purified PCR product. Individual segments were submitted to Eurofins Genomics (Louisville, KY, USA), where they were subjected to library preparation using the Rapid Barcoding Kit 24 V14 (SQK-RBK114.24), followed by priming with the Flow Cell Priming Kit V14 (EXP-FLP004). The prepared libraries were then loaded onto SpotON R10.4.1 flow cells (FLO-MIN114). NanoPack was utilized for the visualization and processing of the generated long-read sequencing data (30), NanoFilt was employed to filter and trim reads based on their length, mean quality, and GC content. Quality control assessments were conducted using NanoStat and NanoPlot. NanoStat provided comprehensive statistical analysis, including mean and median read quality and length, read length N50, and read counts based on quality cutoffs. NanoPlot generated quality control graphs, such as read-length histograms, bivariate plots, and cumulative yield plots. Reads that passed quality control were assembled *de novo* using the Geneious assembler of Geneious Prime (Version 2023.0.1), resulting in the generation of a consensus sequence. The amino acid (aa) sequences of the four viral capsid proteins (σC, σB, μB, and λC) of each ARV strain were manually retrieved from the consensus sequence, and secondary structure was predicted using the EMBOSS tool Garnier of Geneious Prime (31).

### Phylogenetic analyses

The complete genomes of the 15 ARV isolates sequenced in this study, together with publicly available genomes of ARV strains representing well-defined genotypes according to Lu’s classification (7), were used in several phylogenetic analyses. For each ARV, the aa sequences of σC, σB, μB, and λC were segregated by gene and aligned using ClustalW, with the default settings (opening penalty:15 and extension penalty:6.66) in MEGA 11 (32). Each gene alignment was fed to the RAxML (33, 34) with rapid bootstrapping (1,000 replicates) and searched for best scoring ML tree algorithm, with a preset random seed (command line options: -f a -x 1) in Geneious Prime (Version 2023.0.1). Additionally, the aa sequence alignments were used in pairwise distance analysis using MEGA 11 software (35). The phylogenetic relatedness of the tested sequences was estimated using the best-fitting substitution model, and the Nelson Bay reovirus (strains MLBC1313 or WDBP1716) was used as an outgroup. The names and NCBI accession numbers of all the ARV sequences used in this study are summarized in Tables S1 and S2.

### Statistical analysis

#### Survival analysis and phenotypic clustering

Survival was modeled using the number of days until death for each embryo, with embryos still surviving at 7 days being considered right-censored. We used pairwise log-rank tests to compare the survival rates between each pair of avian reovirus strains. The *P*-values were adjusted for multiple comparisons using the Benjamini-Hochberg procedure to maintain the false discovery rate at 0.05. In addition, we fit a Cox proportional-hazards model to generate a χ^2^ likelihood ratio statistic (df = 17) to test whether there was an overall effect of strain on the hazard (cumulative risk of death). Power of the survival analysis was estimated prospectively using a pilot data set with *n* = 5 embryos per strain; a power of 70%–80% was targeted. The power analysis indicated that *n* = 7 embryos per strain would result in 75% power to detect differences in survival between highly and moderately virulent strains and approach 100% power to detect differences between highly virulent and low-virulent strains. Analysis was done in R software v4.5.0 (36) using packages survival v3.8-3 (37), survminer v0.5.0 (38), and powerSurvEpi v0.1.5 (39).

For phenotypic clustering, strains were partitioned into three *a priori* virulence groups (low, moderate, and high) using k-means clustering on two standardized features: 7-day restricted mean survival time (RMST7d) and 7-day mortality (Mortality 7d%). These metrics were selected to jointly encode “how fast” and “how completely” a strain kills. To mitigate local minima, we used multiple random starts (50). Cluster separation was quantified by the average silhouette width (40). To confirm that the derived clusters represented distinct survival patterns, we compared the Kaplan–Meier survival curves across the three virulence clusters using the log-rank test (41, 42).

#### Genotype–phenotype correlation analysis

To evaluate the extent to which molecular genotyping classifications recapitulate phenotypic virulence clusters, each genotyping approach (single-, dual-, triple-, and quadruple-gene strategies) was compared to the survival-derived virulence groups (low, moderate, and high). The agreement between genotype-based classifications and phenotypic clusters was quantified using the Adjusted Rand Index (ARI) (43) and Normalized Mutual Information (NMI) (44). Additionally, χ^2^ tests of independence were performed to assess statistical association, with effect size summarized using Cramér’s V with bias correction (45). Analyses were conducted in Python (scikit-learn v1.7.2, SciPy v1.16.1, pandas v2.3.2, and matplotlib v3.10) (46–49)

To assess whether variation in secondary-structure features of viral proteins predicts phenotypic virulence, we used ordinal logistic regression (proportional odds model) with virulence cluster (low, moderate, and high) as the ordered outcome. Models were fitted using Nagelkerke’s pseudo-R² (50) to evaluate variance explained and were compared by likelihood-ratio tests (LRTs) against null models (51). Predictive performance was further assessed using leave-one-out cross-validation (LOOCV) to estimate classification accuracy, expressed as the percentage of strains assigned to the correct virulence level (52, 53).

##### Sliding-window spatial scan

To localize the regions of sequence most strongly correlated with virulence, we implemented a sliding-window correlation scan across each protein. For each protein, windows of 10, 15, 20, and 25 amino acids were analyzed, with a stride of 5 residues between windows to ensure coverage, following the principle of sliding-window profiling previously established in protein feature analyses (54, 55). For each window, we computed the mean fractional content of α-helix, β-strand, coil, and turn elements (defined as secondary-structure features) using established secondary-structure assignments (56) or predicted states where structural models were unavailable (57). The resulting window-specific structural profiles were then correlated with virulence clusters using Spearman’s rank correlation (ρ) (58). This strategy was used to identify discrete protein regions whose secondary-structure composition exhibited the strongest correspondence with experimentally defined virulence phenotypes, similar to other biological sequence correlation scans (59, 60). To control for multiple testing across overlapping windows, Benjamini–Hochberg false discovery rate (FDR) correction was applied to the *P*-values (61). For transparency, we reported both statistically significant windows (q ≤ 0.10) and the top absolute correlations even if not formally significant. Hotspots were defined as windows with either FDR-adjusted significance or among the strongest |ρ| values within a given protein.

##### Multi-feature family models

In addition to single-protein models, we fitted combined models (dual, triple, and quadruple proteins) to test whether integrative classification improves predictive power. Family-level summaries were derived by ranking models by Nagelkerke R² and LOOCV accuracy, highlighting top-performing feature combinations. In addition, we also calculated the cross-validated coefficient of determination (CV-R²). For each model, the CV-R² was estimated by leaving out one strain at a time, fitting the model on the remaining data, and predicting the excluded strain’s virulence level. This approach reduces bias from model complexity and provides a variance-explained estimate under true predictive conditions.

## RESULTS

### Clinical and histopathological findings associated with ARV infections in field samples

Diagnostic cases of ARV infection in commercial broiler chickens were studied to characterize the clinical signs, age of onset, and associated histopathological lesions. Most cases showed tenosynovitis as the major clinical sign. In terms of age of onset, chickens infected with the ARV strain 23-087 showed clinical signs as early as 5 days old and presented gross hepatic lesions, with ARV recovered from the liver. In contrast, ARV strain 22-460 was isolated from an older bird showing arthritis and caseous peritonitis (Table 1). Several chickens presented with severe gross lesions affecting the pelvic limbs, including hemorrhage and edema surrounding the femorotibiotarsal joint (stifle) and intertarsal joint (hock), as well as rupture of the flexor tendons at the level of the intertarsal joint.

**TABLE 1:** Clinical isolate description

Reovirus isolate	Source	Significant morphological findings^*a*^	Clinical sign	Age	State	Collection year
22-460	Tendon	–	Tenosynovitis	9 weeks	Georgia	2023
22-806	Tendon	Multifocal ulceration with fibrinoheterophilic crusting	Tenosynovitis	43 days	Georgia	2023
ARV_126484	Tendon	–	Tenosynovitis	–	Iowa	2018
22-835	Tendon	–	Tenosynovitis	11 days	Georgia	2023
ARV_115940	Tendon	–	Tenosynovitis	–	Texas	2016
23-087	Liver	Severe, acute, multifocal necrotizing hepatitis	–	5 days	Georgia	2023
ARV_ 106761	Tendon	–	Tenosynovitis	–	Iowa	2014
23-272	Tendon	Bursal hypoplasia and atrophy	–	17 days	Georgia	2023
ARV_94584	Tendon	–	Tenosynovitis	–	Alabama	2012
ARV_122301	Tendon	–	Tenosynovitis	–	Arkansas	2018
ARV_141045	Tendon	–	Tenosynovitis	–	Alabama	2021
ARV_127720	Tendon	–	Tenosynovitis	–	Iowa	2018
ARV_126695	Tendon	–	Tenosynovitis	–	Arkansas	2018
22-861	Tendon	Focal fibrinoheterophilic tenosynovitis and focal vascular thrombi	Tenosynovitis	14 days	Georgia	2023
Alabama	Tendon	–	Tenosynovitis	–	Alabama	2021

^*a*^ Morphological findings refer to gross and histopathological lesions observed in field cases for which primary tissue samples and diagnostic records were available. –, for strains obtained from external repositories (*n* = 9), original histopathology and immunohistochemistry materials were not accessible, and therefore detailed morphological descriptions from the source birds could not be included. All strains were confirmed to be associated with clinical tenosynovitis at the time of original diagnosis.

**Fig 1:**
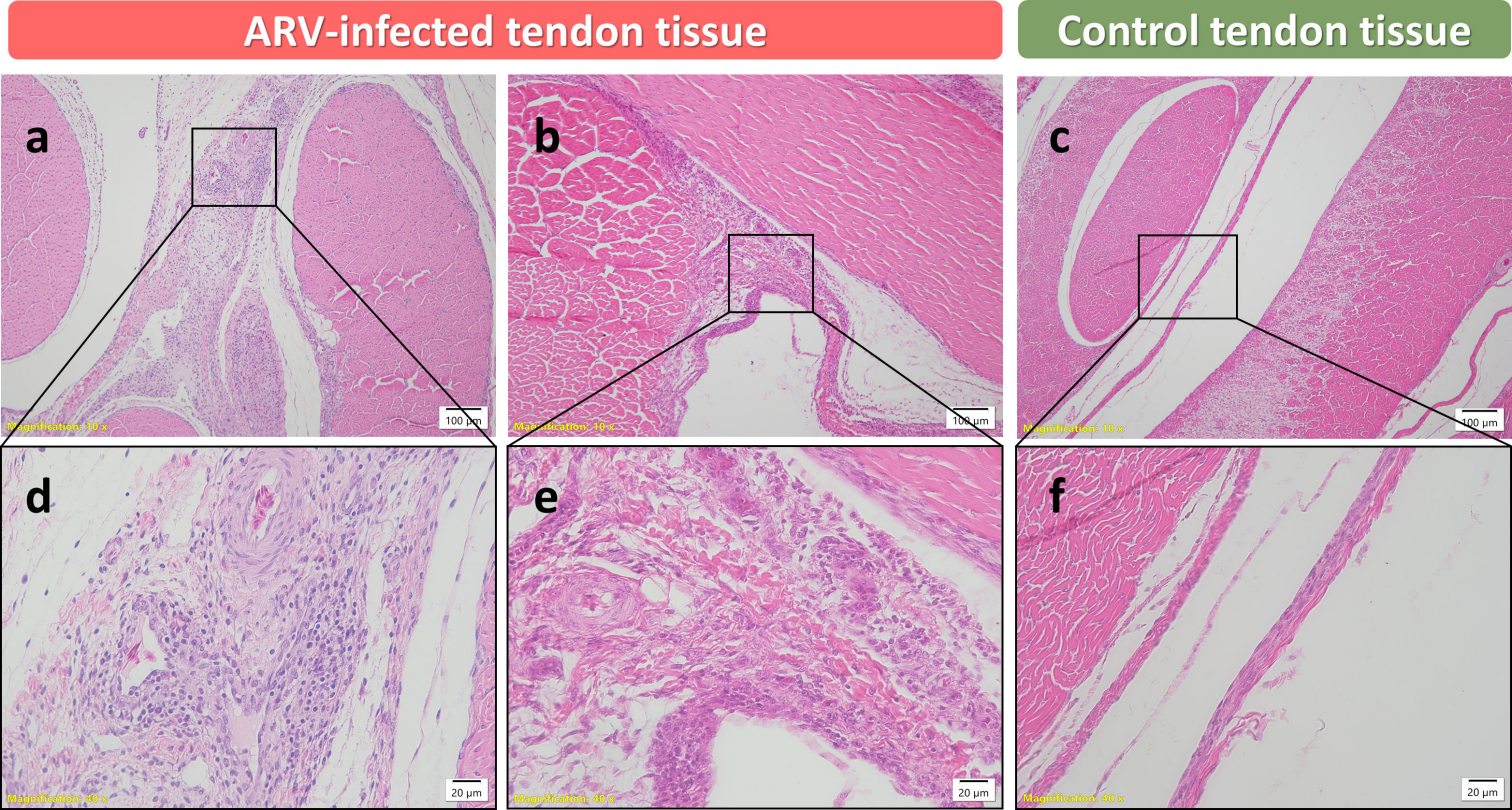
Histopathology in tendon tissue of 14-day-old and 43-day-old ARV-infected broilers. Tendon tissues of 14-day-old broilers infected with ARV strain 22-861 showed multifocal tenosynovitis characterized by synovial cell hyperplasia, fibrin deposition, and mixed inflammatory cell infiltration (**a and d**). Lymphocytic tenosynovitis with synovial cell hyperplasia, fibrin deposition, and lymphoplasmacytic inflammatory cell infiltrates (**b and e**) in 43-day-old chicken infected with ARV strain 22-806. Control tendon tissue (**c and f**). Panels d–f are higher magnifications (40×) of boxed areas in a–c, respectively.

Histopathological examination of available field cases revealed distinct tendon and synovial lesions in chickens infected with specific ARV strains. Strain 22-806 showed moderate synovial cell hyperplasia with mild multifocal lymphoplasmacytic cell infiltration (Fig. 1a and d**;** Fig. S1a through d). In contrast, ARV strain 22-861 exhibited focal fibrinoheterophilic tenosynovitis and focal vascular thrombi (Fig. 1b and e). Although ARV strain 23-272 did not display overt tendon lesions, it was associated with moderate multifocal lymphocyte depletion of the bursa of Fabricius, extending from the medulla to the cortex, along with mild interfollicular edema (Fig. S1e through f). These findings may indicate an immunosuppressive effect of this particular ARV strain.

### Embryonic mortality and pathological findings following ARV infection

**Fig 2:**
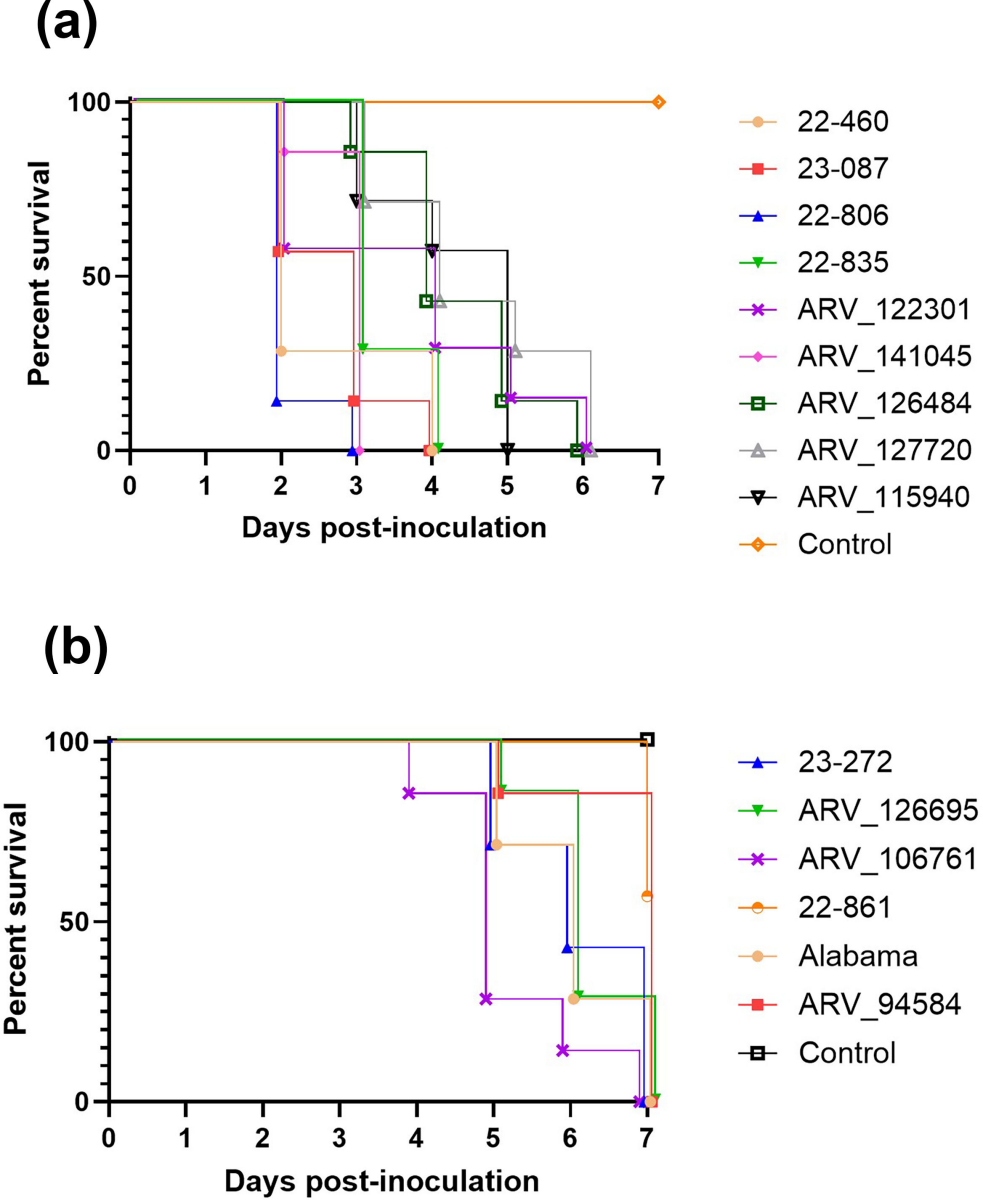
Kaplan–Meier survival plot demonstrating a range of mortality over a 7-day period in specific pathogen-free chicken embryos (*n* = 7 embryos/strain) due to infection with different ARV strains: (**a**) highly virulent strains and (**b**) moderate and low virulent strains.

**Fig 3:**
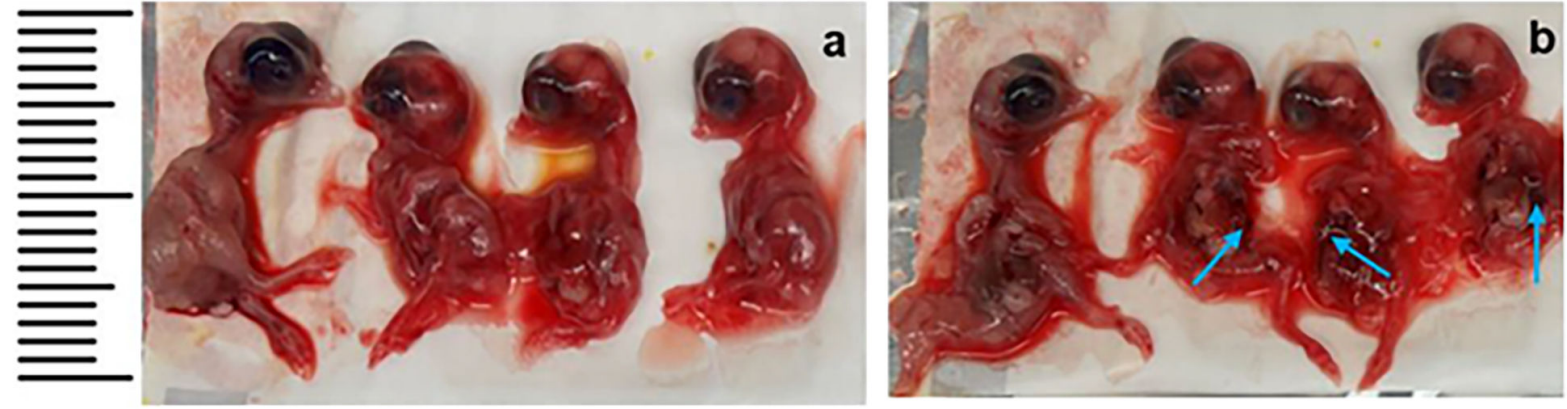
ARV-infected embryos showing typical hemorrhage and stunting. The embryo on the far left is an uninfected control, whereas the second-to-fourth embryos succumbed at 3 days post-inoculation (dpi) after chorioallantoic membrane (CAM) inoculation at embryonic day 10 with 10^3^ PFU of ARV strains 22-835, 22-806, and 23-087, respectively. Panel **a** presents a lateral view illustrating growth retardation and external lesions in comparison to the control, while panel **b** shows a frontal view highlighting internal hemorrhagic lesions. Blue arrows denote the internal visceral hemorrhagic lesions.

Following ARV infection, embryonic mortality rates varied significantly among different ARV strains. The 22-806 and ARV_141045 strains exhibited the highest mortality, resulting in the death of all the embryos within 3 days post-infection (Fig. 2). In contrast, the 22-861 strain resulted in the lowest mortality rate (~43%) at 7 days post-infection. The dead embryos were typically hemorrhagic and stunted (Fig. 3). Surviving embryos displayed no significant lesions.

The pairwise log-rank tests showed that there were significant differences in survival between the strains (Benjamini-Hochberg-adjusted *P* < 0.05, Table S3). The least virulent strain, 22-861, did not significantly differ from the control (*P* = 0.081). All other strains were significantly more virulent than the control (*P* = 0.001). There were many other significant differences. For example, strains 22-806, 22-460, 23-087, and 2177 were significantly more virulent than most of the other strains, all with mean survival times of less than 3 days. The Cox model showed that there was a significant overall effect of strain on survival ($\chi_{17}^{2}$ =195.5, *P* < 2 × 10^−16^).

Phenotypically, we successfully obtained three clusters with good separation (average silhouette = 0.594). Highly virulent strains showed rapid and complete embryonic lethality (*n* = 9): ARV_126484, ARV_127720, ARV_115940, ARV_122301, ARV_141045, 23-087, 22-460, 22-835, and 22-806 (Mean RMST7d = 3.37 days; Mortality7d = 100%; median time-to-death among events ≈ 3 dpi). Moderately virulent strains had complete but delayed lethality compared to the highly virulent group (*n* = 5): ARV_94584, 23-272, ARV_126695, Alabama, ARV_106761 (Mean RMST7d = 6.06 days; Mortality7d = 100%; median time-to-death ≈ 6 dpi). However, the low virulent strain (*n* = 1, 22-861), which was assigned to its own cluster, displayed substantial survival at the end of follow-up with late or no deaths (RMST7d = 7.00 days; Mortality7d = 42.9%; median time-to-death among events ≈ 7 dpi). These clusters reflect a coherent gradient from rapid, early mortality (high virulence) to slower, late mortality (moderate virulence) to partial survival (low virulence), capturing both the tempo (RMST/time-to-death) and extent (mortality %) of lethality (Fig. S2).

**Fig 4:**
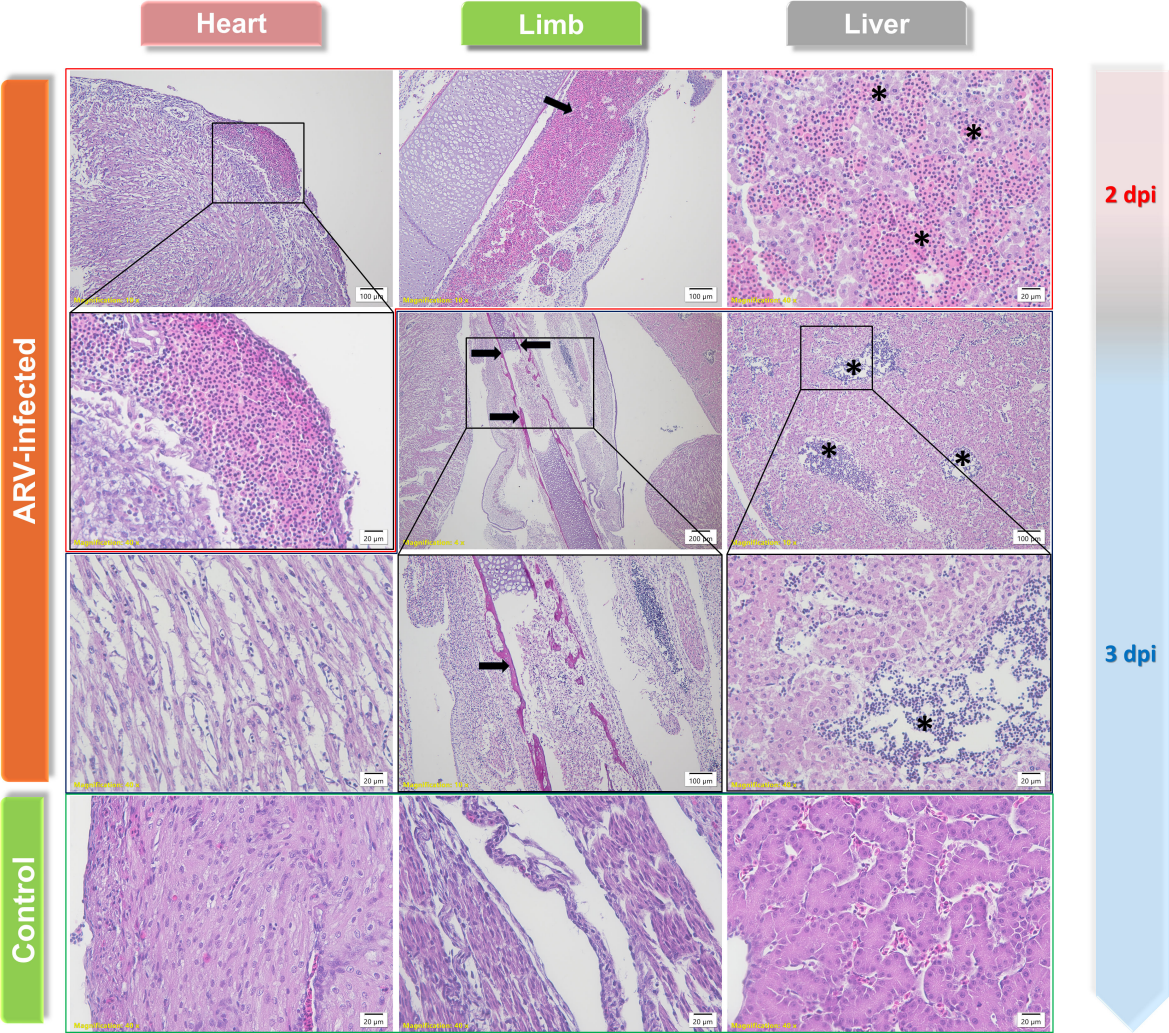
Histopathology of ARV isolate 22-806 in chicken embryos at 2 and 3 days post-infection. Focal epicardial hemorrhage (black box) with myocardial edema and fragmentation of cardiomyocytes in the heart. Multifocal subcutaneous hemorrhage in limbs (arrows). Multifocal hemorrhage of the liver parenchyma (asterisks). Negative control sections of liver, heart, and limbs were obtained from an ARV-uninfected embryo.

Histopathological examination of ARV-infected chicken embryos revealed varying degrees of tissue damage across different ARV strains. Among the highly virulent strains, 22-460 displayed mild multifocal edema in the cardiac interstitium and cardiomyocytes, while 22-806 exhibited more severe cardiac pathology, including focal epicardial hemorrhage, myocardial edema, and fragmentation of cardiomyocytes (Fig. 4). Liver lesions were pronounced in both 22-460 and 22-806, with moderate-to-severe multifocal hemorrhage. Interestingly, mild bile stasis was observed in 22-460 infections. The other highly virulent strain 22-835, caused milder liver lesions (i.e., less severe multifocal hemorrhage and vascular congestion, Fig. S3) compared to strain 22-806 (Fig. 4) but exhibited significant subcutaneous hemorrhage in limb sections. The highly virulent strain 23-087 showed moderate multifocal edema of the interstitium and myocyte fragmentation in the heart, and mild-to-moderate multifocal hemorrhage in the liver, besides moderate multifocal hemorrhage of the interstitium of the kidney (Fig. S3).

IHC revealed distinct DAB-positive immunolabeling of ARV antigens within embryonic tissues, indicating a strong colocalization of ARV antigens with the pathological lesions in the corresponding hematoxylin and eosin (H&E)-stained sections (Fig. S4). In the liver, signals were consistently detected in areas exhibiting multifocal parenchymal hemorrhage and vascular congestion, confirming the presence of viral antigens within hepatocellular regions undergoing degenerative changes. Similarly, in the heart, IHC demonstrated intense antigen deposition in fragmented cardiomyocytes, mirroring the structural damage identified histologically. In the limbs, ARV antigens were localized to foci of subcutaneous hemorrhage and adjacent soft tissues, aligning precisely with H&E-identified pathological changes. Because IHC and H&E staining were performed on serial sections, minor differences in cellular arrangement were observed, particularly in embryonic tissues; these differences are consistent with known effects of antigen retrieval and detergent-based permeabilization during IHC processing and do not indicate biological cell separation.

Importantly, the patterns of IHC staining overlapped with the distribution of histopathological lesions across the various investigated tissues, thereby excluding the possibility of nonspecific antigen detection or incidental pathological findings. Since the embryos were obtained from SPF flocks, the observed colocalization establishes a direct causal link between ARV infection and the associated clinical manifestations in embryonic liver, heart, and limb tissues, ruling out confounding contributions from other avian pathogens or background viral infections.

### Predicted secondary structure-driven triple genotyping approach of μb/λC/σC associated with ARV strain pathogenesis

In this study, we compared the discriminatory power of multiple gene-based genotyping approaches for ARVs, both aa sequence-driven and secondary structure-driven, and assessed their potential to link genetic clustering with strain virulence, as defined by the severity of clinical signs, embryonic mortality, and pathological lesions. Overall predicted secondary-structure features derived from μB, λC, and σC aa sequences were observed to be associated with differences in ARV strain virulence. These analyses indicated that variation in aa composition corresponds to distinct predicted secondary-structure profiles that correlate with experimentally defined virulence phenotypes.

#### Amino acid sequence-driven genotyping failed to predict virulence

**Fig 5:**
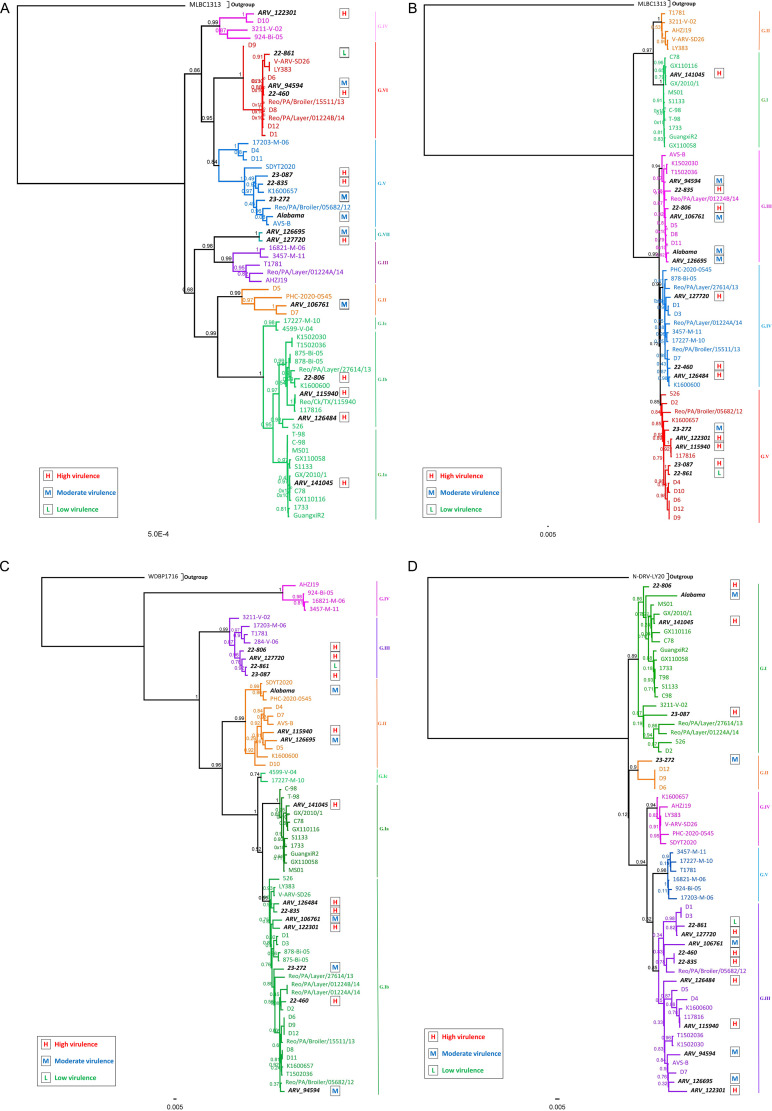
Phylogenetic trees of outer viral protein amino acid sequences using the maximum likelihood method and JTT matrix-based model. The tree with the highest log likelihood is shown. A discrete Gamma distribution was used to model evolutionary rate differences among sites. Each tree shows the phylogenetic relatedness based on the amino acid sequence of σC (**a**), λC (**b**), μB (**c**), and σB (**d**). Each color denotes a different genotypic cluster or subcluster. The study strains are displayed in bold italic font. Clustering probability based on the bootstrapping method is shown at the nodes. The NRV strains MLBC1313 or WDBP1716 were used as an outgroup. Accession numbers, country of origin, and year of collection of the ARVs’ sequences used for the phylogenetic analysis are displayed in Table S2.

Phylogenetic analysis of σC resolved seven clusters (G I–G VII; >70% aa identity, Fig. 5a). Highly virulent strains were restricted to G I (ARV_141045, ARV_126484, ARV_115940, and 22-806) and G IV (ARV_122301), showing closest relationships to reference strains C78 (G Ia, d = 0.000001, Table S4), 526 (G Ib, d = 0.094497), Reo/Ck/TX/115940 and 117816 (d = 0.000001 and 0.0056), the Californian K1600600 (d = 0.03133), and Canadian D10 (d = 0.08023). Moderately virulent strains were either distinct (e.g., ARV_106761 in G II) or co-clustered with highly virulent strains, as in G V (23-272 and Alabama with 22-835, 23-087) and G VII (ARV_126695 with ARV_127720; d = 0.032407). G VI contained strains from all virulence categories (22-460, ARV_94584, and 22-861) closely related to Canadian strains D6 and D12 (d = 0.002864–0.0381789).

In λC phylogeny, five clusters (G I–G V; >95% aa identity, Fig. 5b) were identified. G I and G IV exclusively comprised highly virulent strains (ARV_141045, ARV_127720, 22-460, and ARV_126484), related to GX/2010/1 (d = 0.002303, Table S5), Canadian D1 (d = 0.020944), Pennsylvanian 15511 (d = 0.00999), and Californian K1600600 (d = 0.003842). Other highly virulent strains (22-806 and 22-835) grouped with moderately virulent strains (ARV_94594, ARV_106761, ARV_126695, and Alabama) in G III. By contrast, G V contained strains across virulence tiers, including 23-087 and 22-861, which were nearly identical (99.07% aa identity, d = 0.00923), despite divergent virulence.

µB phylogeny resolved four clusters (G I–IV; >93.2% aa identity, Fig. 5c). G I was the largest, containing highly virulent strains (ARV_141045, ARV_126484, ARV_122301, 22-835, and 22-460) together with moderately virulent strains (23-272, ARV_106761, and ARV_94594). ARV_126484 clustered with Georgia 22-835 (d = 0.011337, Table S6), while ARV_141045 and 22-835/23-272/ARV_122301 were closely related to Chinese strains GX110116 (d = 0.005644) and V-ARV-SD26 (d = 0.011302–0.018512). G II included both highly (ARV_115940) and moderately virulent (ARV_126695, Alabama) strains, whereas G III contained highly virulent (23-087, 22-806, and ARV_127720) and low-virulent (22-861) strains. Consistent with λC results, 23-087 and 22-861 were nearly identical (99.56% aa identity, d = 0.004257).

σB phylogeny identified five clusters (G I–V; >94.5% aa identity, Fig. 5d). Moderately virulent strains formed distinct lineages (e.g., 23-272 in G II, aligned with Canadian D12/D9/D6, 96.73% identity, d = 0.03250, Table S7) or grouped with highly virulent strains (Alabama with 22-806, ARV_141045, and 23-087 in G I). Despite relatedness between 23-087 and 22-806 (96.73% identity, d = 0.032629), 22-806 was more closely related to Chinese strain MS01 (98.37%, d = 0.016184). G III contained the majority of strains (*n* = 10) across virulence levels, including 22-861 (low virulent), closely related to ARV_127720 (high virulent, d = 0.00805). ARV_126695, ARV_94594, and ARV_122301 clustered with U.S. strain AVS-B (d = 0.005359-0.01895), originally associated with runting-stunting syndrome.

Collectively, the multi-gene phylogenies demonstrated that while σC and λC analyses provided the broadest genotypic resolution, µB and σB further refined evolutionary relationships and revealed intra-genogroup diversity. Several strains (e.g., 22-087/22-861, ARV_106761/ARV_115940/117,816, and 22-460/22-835) exhibited near-identical sequence homology yet displayed divergent clinical or pathological outcomes, suggesting that virulence cannot be fully explained by single-gene clustering alone. Conversely, certain phylogenetic signals, such as the association of G V σC/λC clusters and G III σB clusters with embryonic lesions and mortality, support the contribution of multi-locus approaches for linking genotype with phenotype. These findings underscore the importance of incorporating multiple genomic targets to achieve more accurate discrimination among ARV strains and to better capture the complex genetic determinants underlying strain virulence.

To define a robust genotypic classification framework linking ARV genetic content to strain virulence, we first evaluated three strategies based on dual-, triple-, and quadruple-gene combinations of the four outer capsid proteins and compared them against single-gene phylogenies. This yielded six dual-gene schemes, four triple-gene methods, and one four-gene system (Table 2).

**TABLE 2:** Outer ARV protein-mediated genotyping approaches for our clinical strains^*a*^

Genotyping approach		22-806↑	22-861↓	23-087↑	22-835↑	23-272→	22-460↑	Alabama→	ARV_ 141045↑	ARV_ 127720↑	ARV_ 106761→	ARV_ 126484↑	ARV_ 115940↑	ARV_ 94594→	ARV_ 126695→	ARV_ 122301↑
Single-gene based genotyping	MB	**3**	**3**	**3**	**1b**	**1b**	**1b**	**2**	1a	**3**	**1b**	**1b**	**2**	**1b**	**2**	**1b**
	SB	**1**	**3**	**1**	**3**	2	**3**	**1**	**1**	**3**	**3**	**3**	**3**	**3**	**3**	**3**
	SC	**1b**	**6**	**5**	**5**	**5**	**6**	**5**	1a	**7**	2	**1b**	**1b**	**6**	**7**	4
	LC	**3**	**5**	**5**	**3**	**5**	**4**	**3**	1	**4**	**3**	**4**	**5**	**3**	**3**	**5**
Dual-gene-based genotyping	MB-SB	**3.1**	**3.3**	**3.1**	**1b.3**	1b.2	**1b.3**	2.1	1a.1	**3.3**	**1b.3**	**1b.3**	**2.3**	**1b.3**	**2.3**	**1b.3**
	MB-SC	3.1b	3.6	3.5	**1b.5**	**1b.5**	**1b.6**	2.5	1a.1a	3.7	1b.2	1b.1b	2.1b	**1b.6**	2.7	1b.4
	MB-LC	3.3	**3.5**	**3.5**	**1b.3**	**1b.5**	**1b.4**	**2.3**	1a.1	3.4	**1b.3**	**1b.4**	2.5	**1b.3**	**2.3**	**1b.5**
	SB-SC	1a.1b	**3.6**	1b.5	3.5	2.5	**3.6**	1.5	1.1a	**3.7**	3.2	**3.1b**	**3.1b**	**3.6**	**3.7**	3.4
	SB-LC	**1.3**	**3.5**	1.5	**3.3**	2.5	**3.4**	**1.3**	1.1	**3.4**	**3.3**	**3.4**	**3.5**	**3.3**	**3.3**	**3.5**
	SC-LC	1b.3	6.5	**5.5**	**5.3**	**5.5**	6.4	**5.3**	1a.1	7.4	2.3	1b.4	1b.5	6.3	7.3	4.5
Triple-gene-based genotyping	MB-SB-SC	3.1.1b	3.3.6	3.1.5	1b.3.5	1b.2.5	**1b.3.6**	2.1.5	1a.1.1a	3.3.7	1b.3.2	1b.3.1b	2.3.1b	**1b.3.6**	2.3.7	1b.3.4
	MB-SB-LC	3.1.3	3.3.5	3.1.5	**1b.3.3**	1b.2.5	**1b.3.4**	2.1.3	1a.1.1	3.3.4	**1b.3.3**	**1b.3.4**	2.3.5	**1b.3.3**	2.3.3	1b.3.5
	MB-SC-LC	3.1b.3	3.6.5	3.5.5	1b.5.3	1b.5.5	1b.6.4	2.5.3	1a.1a.1	3.7.4	1b.2.3	1b.1b.4	2.1b.5	1b.6.3	2.7.3	1b.4.5
	SB-SC-LC	1.1b.3	3.6.5	1.5.5	3.5.3	2.5.5	3.6.4	1.5.3	1.1a.1	3.7.4	3.2.3	3.1b.4	3.1b.5	3.6.3	3.7.3	3.4.5
Quadruple gene-based genotyping	MB-SB-SC-LC	3.1.1b.3	3.3.6.5	3.1.5.5	1b.3.5.3	1b.2.5.5	1b.3.6.4	2.1.5.3	1a.1.1a.1	3.3.7.4	1b.3.2.3	1b.3.1b.4	2.3.1b.5	1b.3.6.3	2.3.7.3	1b.3.4.5

^*a*^ The bold text with gray highlighting denotes similar genotyping across strains. Genotypes I–VII were replaced by numbers 1–7 for easier visualization. MB, SB, SC, and LC refer to µB, σB, σC, and λC, respectively. The highly, moderately, and low virulent strains are denoted by up-, right-sided, and down arrows, respectively.

Comparative analyses revealed variable concordance between genotyping strategies and the phenotypic clusters derived from embryo survival experiments. Single-protein classifications generally demonstrated the weakest alignment with virulence groups, reflected by low ARI and NMI values (Fig. S5). Dual-protein strategies showed improved but inconsistent correspondence. Specifically, the σB+λC combination achieved the highest ARI (0.055) with moderate NMI (0.364); however, this was not statistically significant (χ² *P* = 0.365; Cramér’s V = 0.088) (Fig. S5). The λC single-gene model performed slightly lower (ARI = 0.030). Multi-gene approaches (triple/quadruple) produced higher NMI values (~0.45–0.48), indicating shared information with virulence groupings, although ARI remained near zero and χ² tests were non-significant. Clustering based on patristic distances further revealed heterogeneity in concordance depending on the gene(s) analyzed. The λC+σB pair achieved the highest performance (ARI = 0.060, NMI = 0.193, Cramér’s V = 0.168, Table S8), although again without statistical significance (χ² *P* = 0.286). In conclusion, the indices, including ARI, NMI, and effect size metrics, suggest that sequence-based phylogenetic clustering alone cannot robustly predict virulence.

#### The secondary structure of μB+λC+σC triple model exhibited the highest virulence predictive performance

**Fig 6:**
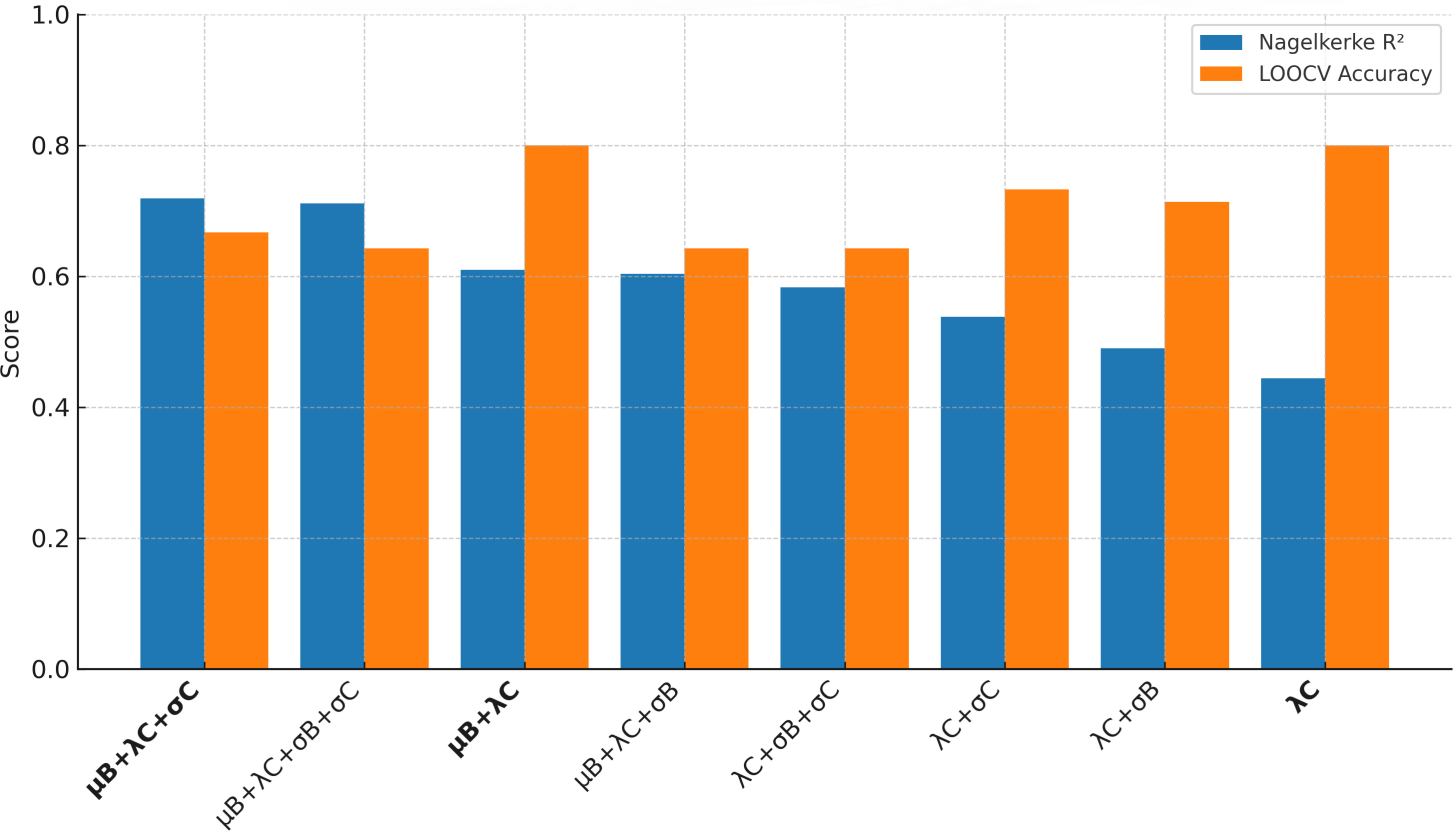
Family-level model performance in predicting virulence clusters. Bar chart shows Nagelkerke R² (blue) and LOOCV accuracy (orange).

The secondary structure analysis of the four proteins yielded differential abundance patterns of the secondary structure features. Logistic regression models were used to test if the structural features of μB, λC, σB, and σC, individually or in combination, could be used as virulence predictors. Among the evaluated models, the μB+λC+σC triple model explained the greatest proportion of variance in virulence under cross-validation, whereas the μB+λC dual model achieved the highest leave-one-out cross-validation (LOOCV) accuracy. These results indicate complementary strengths across models, reflecting a trade-off between variance explanation and prediction accuracy. For instance, the μB+λC+σC triple model provided the strongest predictive performance, with a Nagelkerke R² of ~0.80 and LOOCV accuracy of ~71% (Fig. 6). Both the λC single-gene model and the μB+λC dual model achieved LOOCV accuracy values approaching 80%, highlighting the predictive strength of λC and μB when integrated. To reduce potential overestimation associated with multivariable modeling in small data sets, CV-R² was calculated and used as a criterion for model comparison. The CV-R² values revealed clear stratification in predictive utility. Single-protein models explained only ~0.21–0.27 of variance under cross-validation, whereas the λB+σB dual model improved to 0.33. The μB+λC+σC triple model achieved 0.41, and the full four-protein model 0.47, demonstrating that integrative models captured an additional predictive signal without overfitting.

### Secondary structure-based candidate regions for ARV strain differentiation

**Fig 7:**
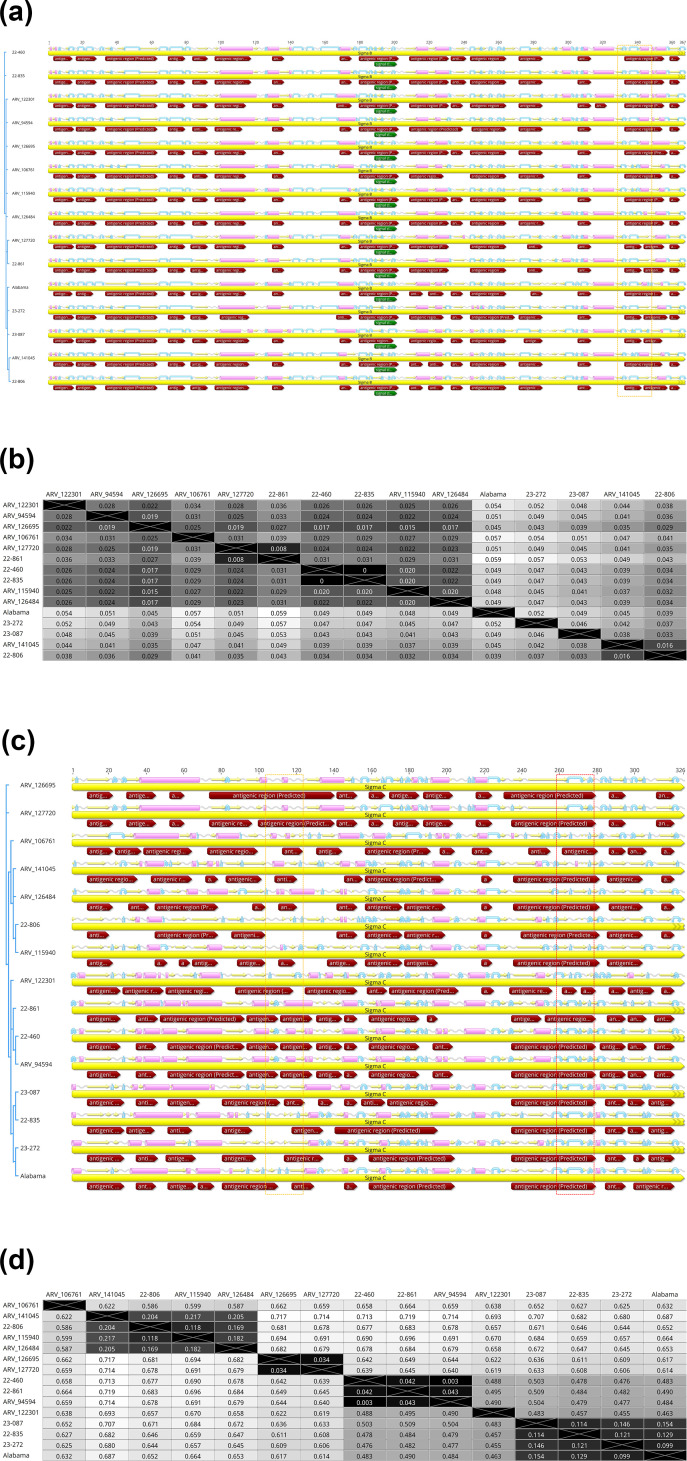
Amino acid sequence analysis and secondary structure prediction of σB (**a**) and σC (**c**) of our ARV strains, and the corresponding heatmaps of σB (**b**) and σC (**d**), denoting the patristic distances indicating the evolutionary relationship based on sequence identity, which was also reflected on the dendrograms on the left side of each figure. The darker the color in the heatmap, the more closely related the strains are to each other. The dashed boxes indicated the regions differentiating between our ARV strains based on the concluded secondary structure. The red-dashed box in σC refers to the potential candidate region in discrimination capacity, whereas the orange dashed boxes in σB and σC refer to the secondary candidate regions. The red arrows indicate the predicted antigenic regions mapped along the sequence, whereas the green arrows refer to the probable cleavage signals. The color coding of the secondary structures is as follows: pink barrels are α-helix, yellow arrows are β-strand, blue-curved arrows are turns, and white spirals are the coil structure.

Secondary structure comparisons of the four outer capsid proteins revealed that σB was the most structurally conserved, followed by λC. Specific regions enriched for predicted secondary-structure features were identified as being statistically associated with virulence clusters. These regions represent candidate sequence features whose predicted structural properties may contribute to phenotypic differences among strains. Hotspot analysis identified these structural motifs with differential distribution across virulence clusters (Table 3; Fig. S6). Within λC, the coil-rich region spanning residues 680–699 (Fig. S7a) exhibited the strongest correlation with virulence (ρ = +0.65, *P* = 0.012, Table 3). Despite the high conservation of σB overall, its C-terminus (aa 244–361) was highly discriminative among strains (Fig. 7a). Hotspot analysis highlighted a helix motif at residues 329–348, although its correlation with virulence was modest (ρ = +0.19, *P* = 0.541). In contrast, μB contributed a turn motif at residues 207–226 (Fig. S7c) with negative correlation (ρ = −0.40, *P* = 0.162). σC displayed the greatest variability among strains, with two discriminative regions: the N-terminus (aa 54–145) and C-terminus (aa 248–295). The C-terminal region, located in the globular head of the spike protein, appeared more critical; a coil motif at residues 259–278 (Fig. 7c) moderately correlated with virulence (ρ = +0.20, *P* = 0.499), while an upstream helix motif (residues 104–123) showed weaker but notable association (ρ = −0.18, *P* = 0.547). Although not all correlations were statistically significant, the consistent distribution of structural motifs across virulence clusters was stable across window sizes (10–25 aa; Fig. S8).

**TABLE 3:** Primary and secondary hotspots from sliding-window analysis^*a*^

Protein	Tier	Window	Feature	Spearman ρ	*P* (Spearman)	Q (Spearman)	Low	Moderate	High
μB	Secondary	207–226	Turn	−0.40	0.162	0.915	0.40	0.38 (0.33–0.42)	0.32 (0.23–0.40)
**λC**	**Primary**	**680–699**	**Coil**	**0.65**	**0.012^*b*^**	**0.177**	**0.05**	**0.11 (0.09–0.14**)	**0.17 (0.13–0.20**)
σB	Secondary	289–308	Helix	0.00	0.996	0.996	0.28	0.14 (0.00–0.29)	0.17 (0.07–0.27)
σB	Secondary	329–348	Helix	0.19	0.541	0.996	0.00	0.16 (0.05–0.28)	0.15 (0.05–0.25)
**σC**	**Primary**	**259–278**	**Coil**	**0.20**	**0.499**	**0.894**	**0.05**	**0.14 (0.00–0.31**)	**0.17 (0.07–0.27**)
σC	Secondary	104–123	Helix	−0.18	0.547	0.914	0.60	0.11 (0.00–0.24)	0.15 (0.03–0.27)

^*a*^ All values are outputs from the sliding-window pipeline (20 aa, stride 5) using the dominant-label method and ≥70% residue coverage per window. Low group shows a single-strain mean (no CI). Moderate/High shows mean ± 95% CI.

^*b*^ Significant at *P* < 0.05. Primary hotspots are highlighted with gray shading and bold type.

Finally, amino acid sequence variation not only shaped secondary structure clustering but also altered mapping of predicted antigenic epitopes. Antigenicity predictions demonstrated variable epitope mapping across strain clusters, except for σB epitopes, which were highly conserved across all groups (Fig. 7).

## DISCUSSION

Arthritis/tenosynovitis syndrome has been consistently documented in domestic poultry (62, 63). Over the past decade, the United States has experienced a marked increase in ARV diagnoses across broiler, layer, and turkey flocks, reflecting the continued emergence and spread of novel strains (16, 19, 64–69). Despite these trends, significant knowledge gaps remain concerning the pathogenic potential of circulating strains, the molecular determinants of virulence, the risk of cross-species transmission, and the efficacy of existing vaccines. Addressing these gaps is crucial for developing updated disease control strategies. In this study, we characterized ARV isolates from clinical cases to link molecular features with pathogenic outcomes. Field metadata revealed that ARV strain 23-087 induced clinical signs as early as 5 days post-hatch, whereas strain 22-460 produced symptoms only at 9 weeks. A recent investigation of ARV field variants in Pennsylvania (2017–2022) similarly reported early clinical onset in some broiler flocks as young as 7 days, while others remained susceptible until ~11 weeks, with younger birds consistently more vulnerable (70). The ARV strains examined here were primarily recovered from tendons of birds with a history of hepatitis and arthritis/tenosynovitis, consistent with previous reports identifying ARV in tendon, joint, and liver tissues (7, 10, 21, 71).

Histopathological examination revealed a wide spectrum of tendon lesions in the affected birds ranging from absent to severe tenosynovitis, with synovial cell hyperplasia, lymphoplasmacytic infiltration, and vascular thrombi. Several studies reported acute inflammatory responses on the synovium covering the joint surfaces and tendon sheaths (tenosynovitis) (72) and lymphocytes infiltrating into dense aggregates in multifocal areas or diffuse through the entire length of the tendon sheath (21). However, the 23-272 strain infection was unique in its association with multifocal lymphocyte depletion in the bursa of Fabricius, despite the absence of overt tendon lesions. This finding aligns with the immunosuppressive effects previously attributed to ARV (73–75), and lymphoid depletion was documented due to the emergence of genetic variants (76). Immunohistochemistry of FFPE sections confirmed that ARV antigens colocalized precisely with histopathological lesions, including hepatic hemorrhage, cardiomyocyte fragmentation, and limb hemorrhage. This spatial association strongly supports the possibility of a direct causative role of ARV in lesion development rather than opportunistic involvement. Previous studies using IHC have similarly demonstrated colocalization of ARV antigens with tissue lesions (29, 77). A limitation of this study is the unavailability of original histopathology for several externally sourced strains, which restricted retrospective morphological comparison in field cases. Nevertheless, all isolates were uniformly evaluated in the controlled embryo infection model, providing a standardized framework for phenotypic virulence assessment across the full strain panel.

Embryonic mortality has served as a reliable indicator of strain pathogenicity in several studies (5, 78–80). Previously published data showed that the highly pathogenic ARV-176 strain caused 80%–100% mortality within 4–5 days, whereas the low-virulent ARV-138 strain resulted in only 10% mortality (78). Consistent with these findings, our highly virulent strain 22-806 and ARV_141045 caused the earliest embryonic mortality at 3 dpi, leading to 100% mortality. In contrast, the strain 22-861 resulted in 42.9% mortality by 7 dpi. Our virulent strains killed embryos mostly within 3–4 dpi, whereas previous studies reported that virulent reoviruses induced embryonic death within 4 (79) or 5–6 dpi, often accompanied by hemorrhagic lesions and a necrotic liver (5). The partial discrepancy could be owing to the differences in the inoculation dose (10^3.5^ TCID_50_ rather than 10^3^ PFU) or administration route (yolk sac rather than CAM route).

The correlation between ARV strain virulence and its molecular genotype remains elusive, primarily due to the limitations of the prevailing σC-based genotyping system (11). Despite classifying strains into the same genotype (genotype 1), significant variations in clinical signs, lesion severity, and viral loads have been observed among ARV strains and the classical vaccine strain S1133 (76). Additionally, the L3 and M2 genes have exhibited substantial molecular divergence (16, 76). λC, encoded by L3, is responsible for viral mRNA capping required for virus replication, mediated by guanylyltransferase activity (81, 82). ARV strains with higher *in vivo* replication capabilities were found associated with greater pathogenicity (6). µB, the major outer capsid protein encoded by the M2 segment, is associated with virion stability, membrane association, and the production of infectious sub-virion particles, ultimately leading to apoptosis (83, 84). Moreover, previous studies have established σB as a type-specific marker, inducing broadly neutralizing antibodies similar to σ3 in mammalian reovirus (85). Therefore, we explored the potential of using the turret and capsid proteins, either alone or in combination with σC, to enhance strain segregation and elucidate potential virulence-genome associations. Our findings demonstrate that single-gene phylogenetic analyses, particularly those based on σC, provide valuable genotypic resolution of ARV strains; however, they are insufficient to fully account for phenotypic variation in virulence. This observation is consistent with earlier reports that σC, while serving as the major antigenic determinant and a key marker for genetic classification, does not reliably predict pathogenicity (76, 86). By integrating λC, µB, and σB analyses, we observed intra-genogroup variability, particularly within σB cluster III, where strains with near-identical sequences (22-460/22-835) nevertheless exhibited mortality and embryonic lesions despite minimal histopathological changes, but still statistically insignificant based on ARI, NMI, and effect metrices. Such discordance suggests that ARV virulence is polygenic, shaped by the interplay of structural and non-structural proteins rather than any single locus (17, 18, 26). Non-structural proteins and replication-associated factors encoded by other genomic segments may modulate viral fitness, host responses, and tissue tropism. The present analysis, therefore, represents a focused, structure-informed subset of potential contributors, and future studies incorporating a broader genomic scope are warranted. Agreement metrics such as ARI and NMI provide useful measures of correspondence between genotype-based clustering and phenotypic virulence groups; however, their interpretability is limited in small data sets and in scenarios with highly imbalanced cluster sizes (87–89). In particular, the presence of a single low-virulence strain constrains the maximum attainable ARI, even when biologically meaningful associations are present (87, 88). Consequently, low ARI or NMI values should not be interpreted as evidence against biological relevance but rather as a reflection of statistical constraints inherent to the study design.

Previously, a difference in the secondary structure of the predicted antigenic epitopes was deduced between ARVs belonging to various genotyping clusters due to aa substitutions as informed by σC structural modeling and antigenicity prediction (90). Our study provides evidence that aa sequence variation in ARV structural proteins is associated with differences in virulence and that these sequence differences correspond to distinct predicted secondary-structure features. While these findings suggest a potential role for protein structural organization in modulating pathogenicity, the secondary-structure component of this analysis is predictive in nature. Among the identified regions, the λC turret protein coil motif spanning residues 680–699 demonstrated the strongest association with virulence. The turret protein λC is known to play a central role in RNA capping and genome stability, and variation in its structural domains has previously been linked to altered pathogenicity and strain differences in orthoreoviruses (82, 91). Structural flexibility within coil-dominated motifs may influence RNA processing efficiency and viral fitness, thereby modulating virulence.

The σC attachment protein is another key determinant of virulence, functioning as the cell-attachment spike protein of ARV. Prior studies have shown that mutations and structural rearrangements in σC affect receptor binding and tissue tropism, with consequences for viral pathogenesis in chickens (92–94). Our detection of a coil-enriched motif between residues 259 and 278, and a candidate helix motif at residues 104–123, is consistent with this functional role (93). Flexibility in σC secondary structure may mirror analogous domains in rotavirus VP4, where conformational changes drive receptor recognition and immune evasion (95, 96).

By contrast, μB and σB exhibited weaker correlations, although their inclusion in integrative models enhanced predictive power. The μB protein is involved in membrane penetration during cell entry, and studies in mammalian orthoreoviruses (MRV) have shown that secondary-structure changes in analogous μ1 proteins alter entry efficiency and viral spread (97, 98). Similarly, σB corresponds to σ3 in MRV, a major capsid protein, contributing to virion stability, and helix–coil balance may influence capsid resilience under environmental stress (99). While the associations we observed for μB (turn 207–226) and σB (helix 329–348) were weaker, they remain biologically plausible contributors to virulence phenotypes. Together, the parallels suggest that multi-protein, structure-aware models may provide more accurate predictors of viral virulence than analyses of single motifs in isolation. Future studies employing experimentally resolved structures or targeted mutagenesis will be required to directly test whether the predicted secondary-structure differences identified here causally influence viral fitness and virulence. Moreover, comparison of predictive models highlights an important trade-off between parsimony and integrative explanatory power. The μB+λC dual model demonstrated superior LOOCV accuracy, suggesting strong classification performance without unnecessary parameters. In contrast, the μB+λC+σC model consistently explained a larger fraction of variance under cross-validation (CV-R²), indicating that σC contributes additional, non-redundant information relevant to virulence. It is important to pinpoint that the aa sequence variations among our strains’ sequences have not only shown diverse secondary structure affinities but also resulted in different antigenic epitope mapping. Therefore, this high variability among the predicted antigenic epitopes between the different clusters and the S1133 vaccine strain could explain the reasons behind the failure of commercial vaccines against the circulating ARV strains. Conversely, the predicted antigenic epitopes on the σB secondary structure of our strains are highly conserved. Previous studies have suggested that Sigma B proteins contain group-specific epitopes (21, 75). Importantly, the novelty of the present work lies not in establishing σB as a protective antigen *per se*, but in demonstrating—within a structure-informed, multigene analytical framework—that σB remains conserved across genetically and phenotypically diverse ARV strains, including those associated with severe pathogenic outcomes.

Our analyses were conducted at the aa level to emphasize functional and structural consequences of sequence variation. Protein-based approaches reduce noise introduced by synonymous substitutions and more directly reflect features relevant to virion architecture and host interaction (100–102). Nonetheless, nucleotide-based phylogenetic analyses may offer complementary insights, particularly for detecting reassortment, synonymous evolutionary pressures, or regulatory RNA features (103–106). Integrating nucleotide- and protein-level frameworks represents an important direction for future work.

In conclusion, the current study addressed major questions involving (i) pathogenesis of ARV strains and its probable linkage to embryonic mortality and clinical lesions, (ii) the secondary structure-informed multi-gene dependent genotyping approach to the relatedness to ARV strain virulence levels, and (iii) the discrimination power of structural motifs with differential distribution across virulence tiers. Our findings suggest that the triple integrative structural system, μB+λC+σC, could provide a more accurate and efficient method for ARV classification. This system could facilitate the linking of ARV strains to specific pathogenic phenotypes, thereby reducing the need for extensive animal experimentation. Furthermore, our findings reinforce prior observations that σB is more conserved than σC and highlight its potential relevance for future vaccine development. While direct evidence of broad protective efficacy was beyond the scope of this study, the conserved nature of σB across diverse ARV strains supports its prioritization for targeted immunogenicity and protection studies.

## Data Availability

The L3, M2, S1, and S3 genomic segments of our ARV strains were deposited in GenBank with accession numbers PQ381497-PQ381520. The sequences used in this study for phylogenetic analysis are openly available in the NCBI GenBank repository with the accession numbers mentioned in Tables S1 and S2.

## SUPPLEMENTAL MATERIAL

Supplemental figures jvi.01982-25-s0001.docx Figures S1 to S8.

Supplemental tables jvi.01982-25-s0002.docx Tables S1 to S8.

ASM does not own the copyrights to Supplemental Material that may be linked to, or accessed through, an article. The authors have granted ASM a non-exclusive, world-wide license to publish the Supplemental Material files. Please contact the corresponding author directly for reuse.
