# Supplementary material for: Novel multigene molecular characterization of avian reovirus strains and associated embryonic pathogenicity
Source: J Virol. 2026 May 5;100(6):e01982-25. doi: 10.1128/jvi.01982-25 (PMC13288925; doi:10.1128/jvi.01982-25)
Supplement: Supplemental figures — Figures S1 to S8. [file jvi.01982-25-s0001.docx]

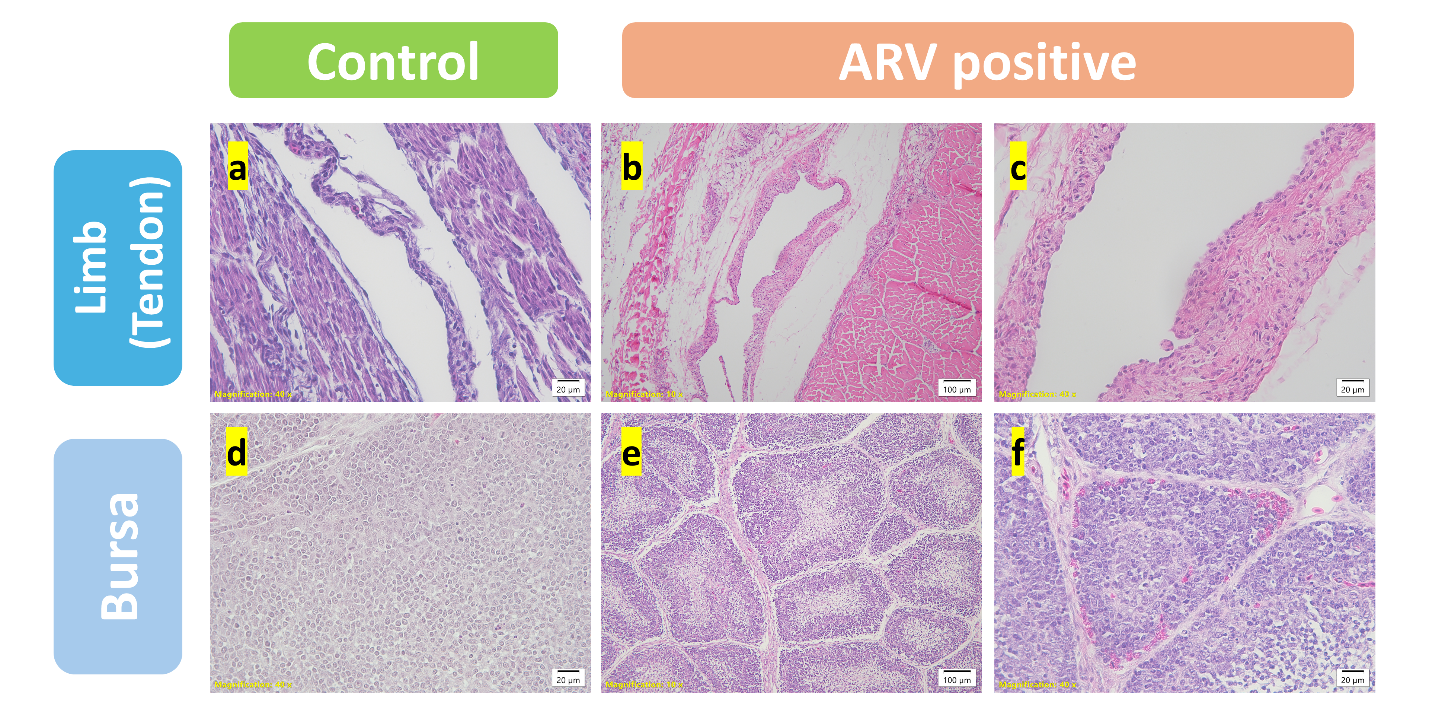


**Figure S1. Histopathology of ARV-infected broilers.**

(a, d) uninfected limb and bursal tissues. (b) moderate synovial cell hyperplasia with mild multifocal lymphoplasmacytic cell infiltration in chicken broiler infected with ARV strain 22-806. (e) moderate multifocal lymphocyte depletion of the bursa of Fabricius, extending from the medulla to the cortex, along with mild interfollicular edema due to infection with 23-272 strain. (c, and f) are magnification (40x) of (b, and e, respectively).


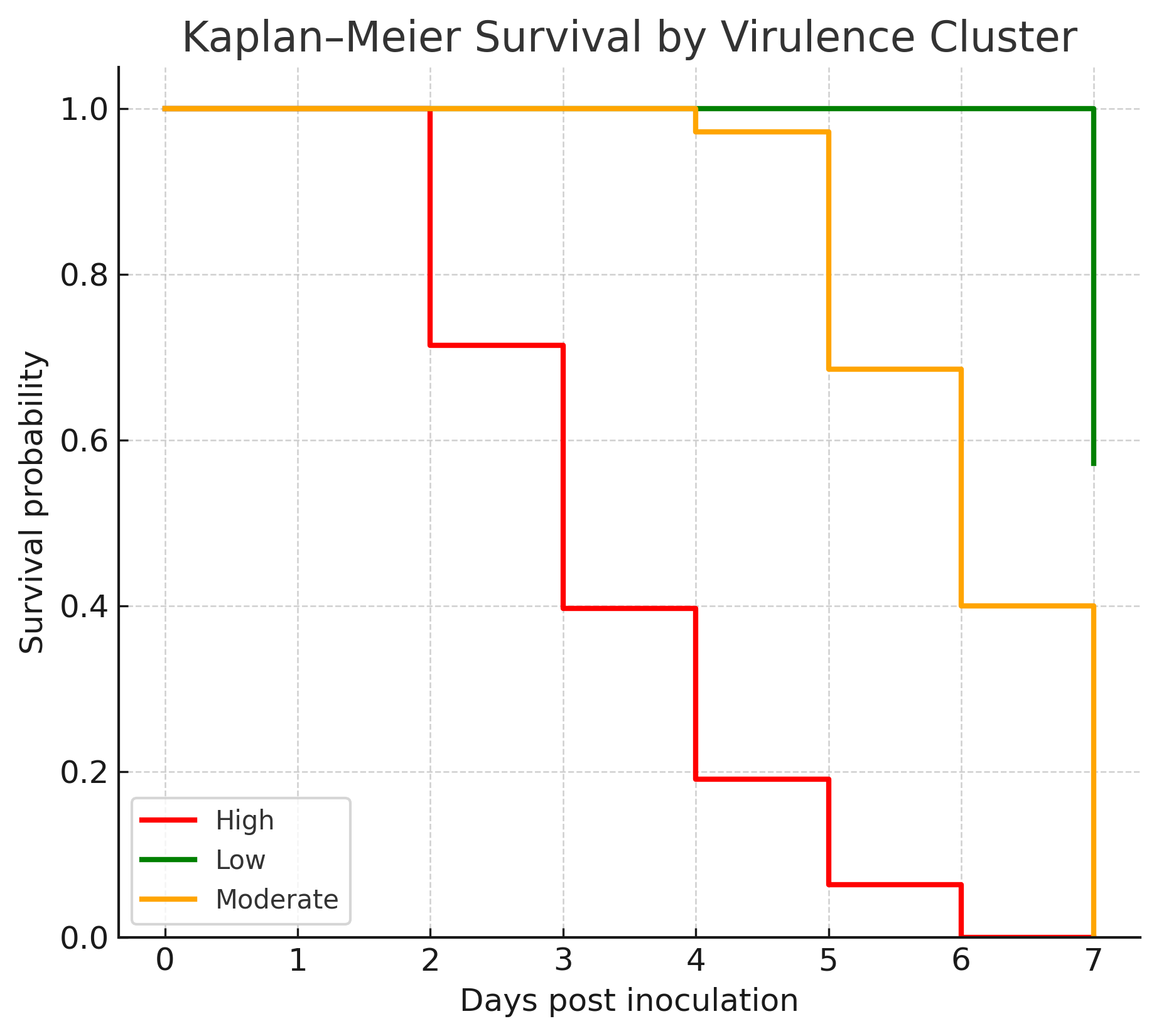


**Figure S2. Kaplan–Meier survival curves by virulence cluster.**

These clusters reflect a coherent gradient from rapid, early embryonic mortality (due to high virulence of specific avian reovirus strains, denoted by red line) to slower, late mortality (moderate virulence, orange line) to partial survival (low virulence, green line) derived from the embryo infection model.


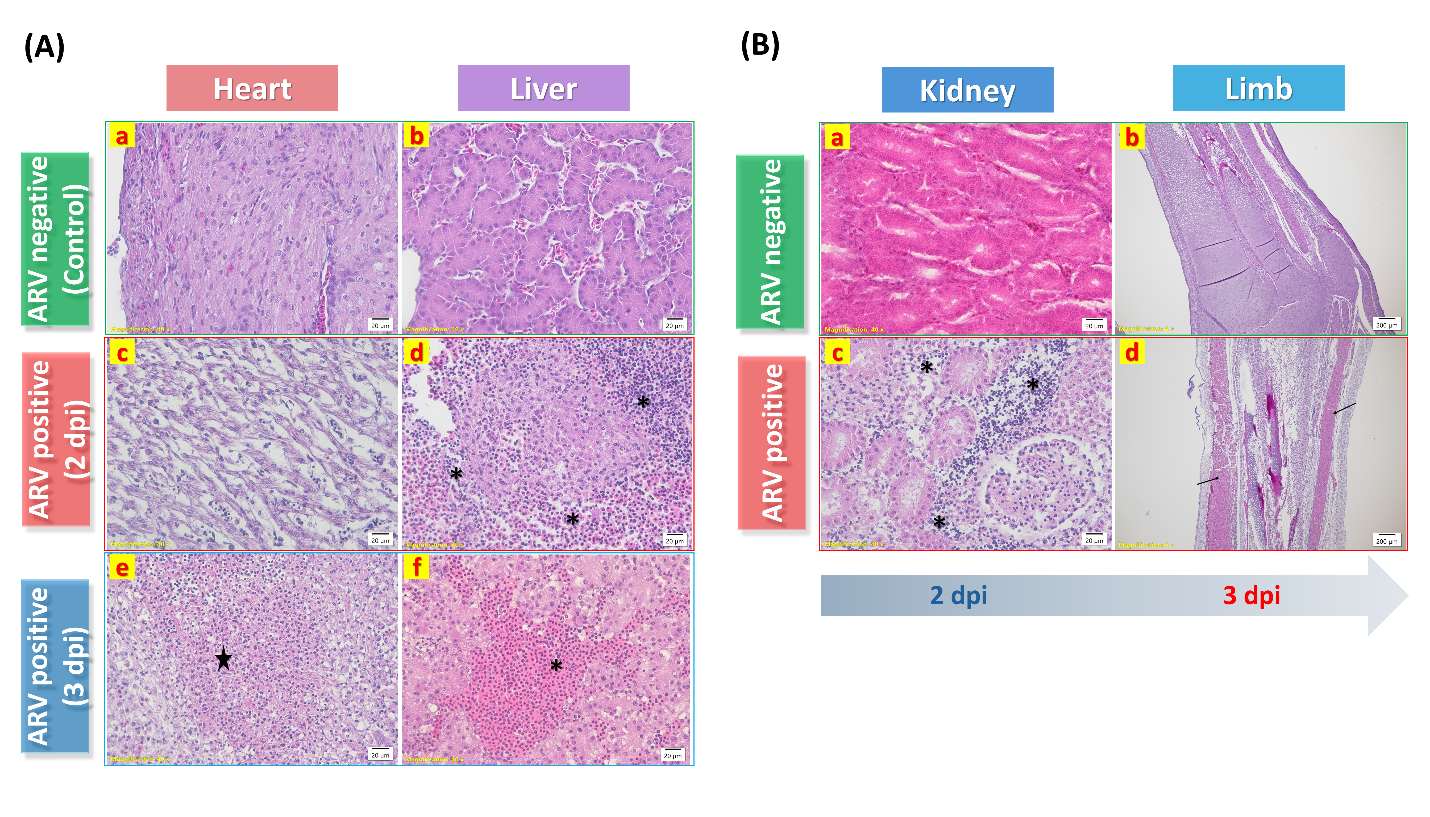


**Figure S3. Embryonic histopathology of ARV strains**: (A) Uninfected embryonic heart and liver tissues in (a, and b), 23-087 in (c) heart showing moderate multifocal edema of interstitium with thinning and fragmentation of cardiac myocytes, (d) liver demonstrating mild to moderate multifocal hemorrhage (asterisks) at 2dpi, and 22-835 strain in (e) heart showing mild to moderate multifocal degeneration and necrosis of cardiomyocytes with mononuclear inflammatory cell infiltration (star), and (f) liver with mild multifocal hemorrhage (asterisk) and vascular congestion at 3dpi. (B) Control embryonic kidney and limb in (a, and b), 23-087 in (c) Kidney showing moderate multifocal hemorrhage of interstitium (asterisks), and 22-835 strain in (d) limb section depicting subcutaneous moderate to severe multifocal hemorrhage (arrows).


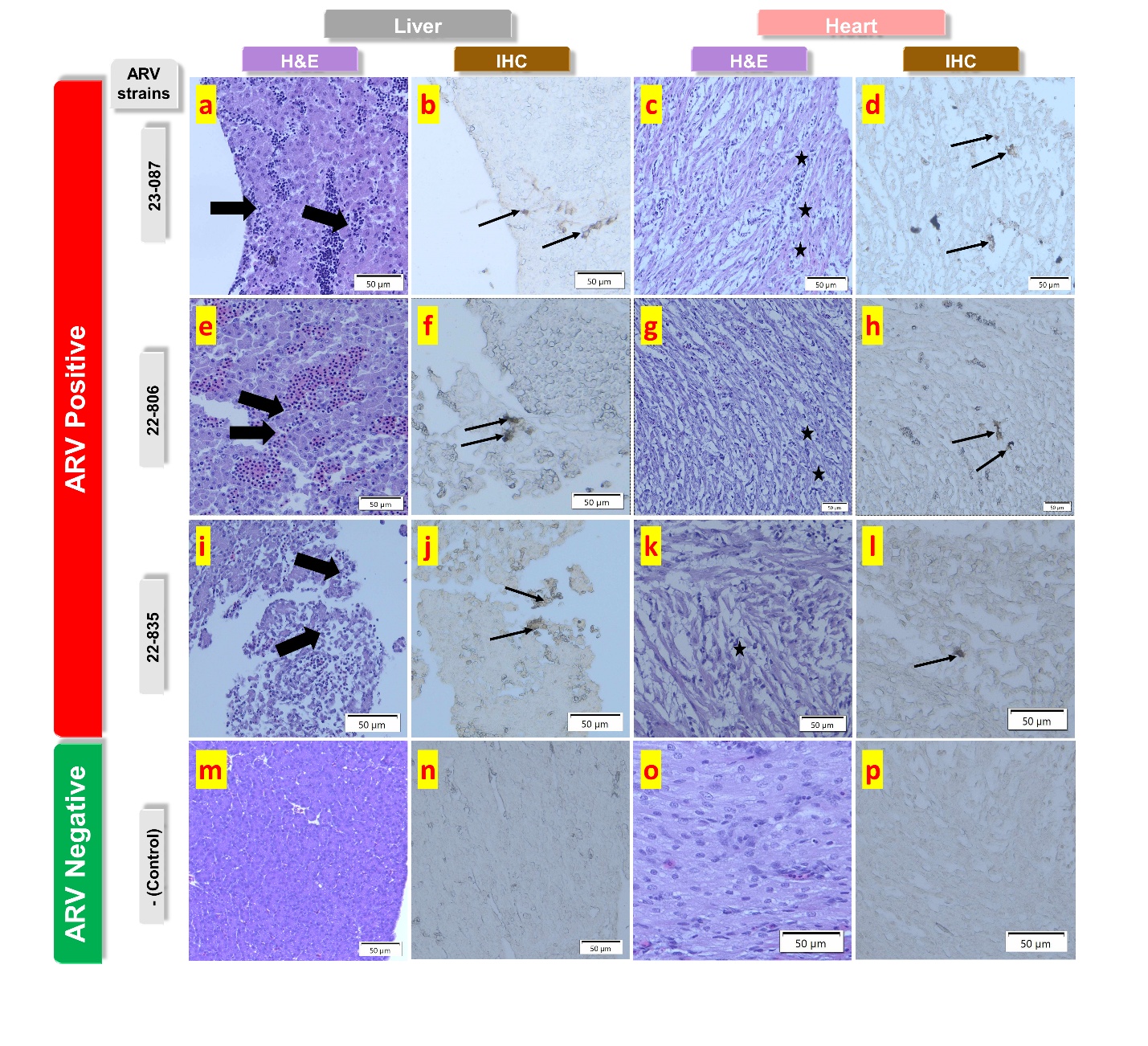


**Figure S4.** **Histopathology and detection of ARV isolates in chicken embryos at 2 and 3-days post infection by hematoxylin and eosin (H&E) and immunohistochemistry (IHC) in liver, and heart.** (a, e, and i) Multifocal hemorrhage of the liver parenchyma (arrows), H&E. (c, g, and k) Myocardial edema and fragmentation of cardiomyocytes in the heart (stars), H&E. (m and o) Liver and heart tissue from ARV uninfected embryos. (b, d, f, h, j, and l) Serial sections of (a, c, e, g, i, and k) showing positive staining for reovirus by IHC (brown, arrows). (n and p) Serial sections of (m, and o) showing no brown staining. Images shown represent typical staining patterns observed in multiple embryos and tissues examined for representative ARV strains from the high-virulence group (ARV strains are shown on the left side). Differences in cellular spacing relative to H&E are attributable to IHC processing steps, including antigen retrieval and permeabilization, which can accentuate intercellular boundaries without reflecting true tissue dissociation.


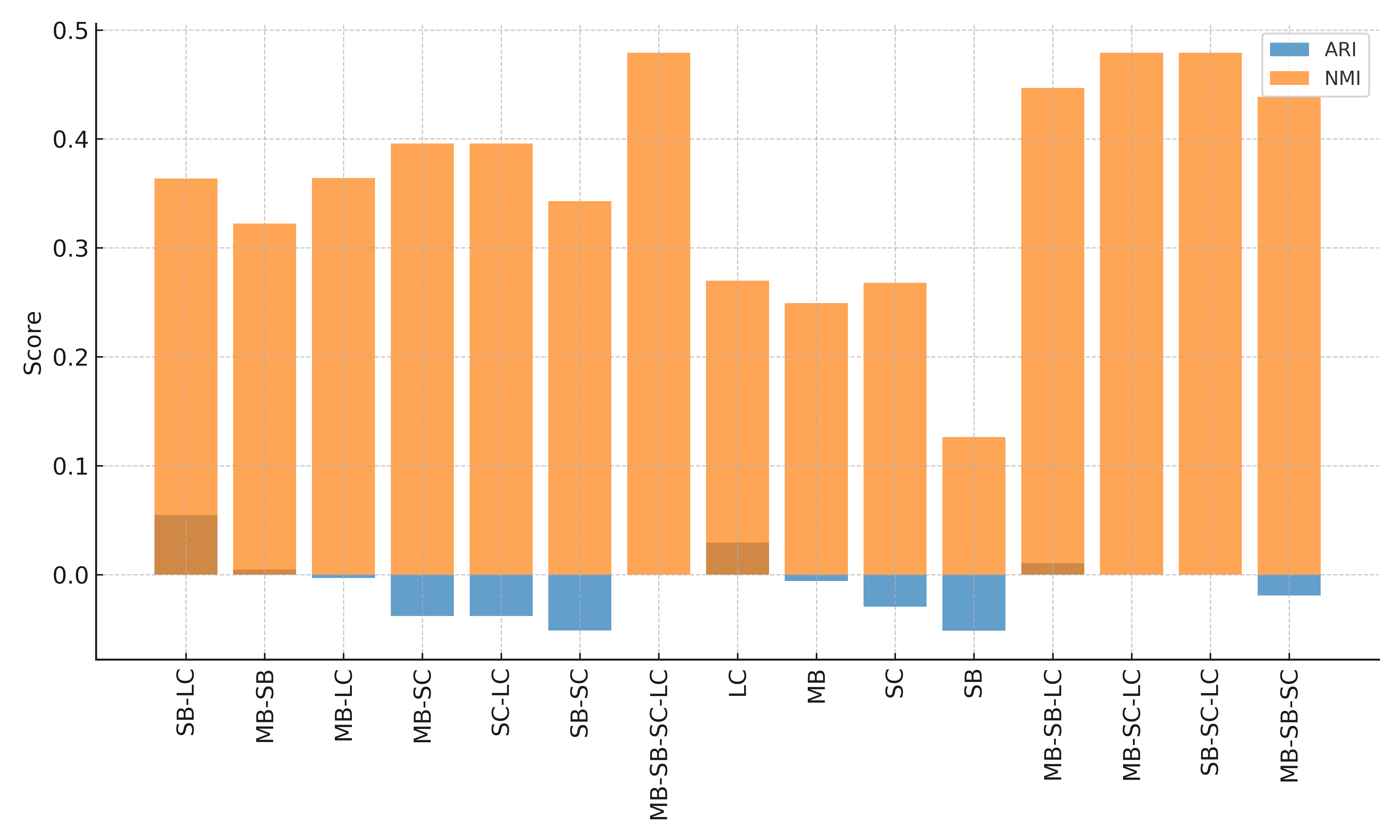


**Figure S5.** **Adjusted Rand Index (ARI) and Normalized Mutual Information (NMI) scores for each genotyping approach.**

The agreement metrics, ARI and NMI, provide useful measures of correspondence between genotype-based clustering and phenotypic virulence groups.


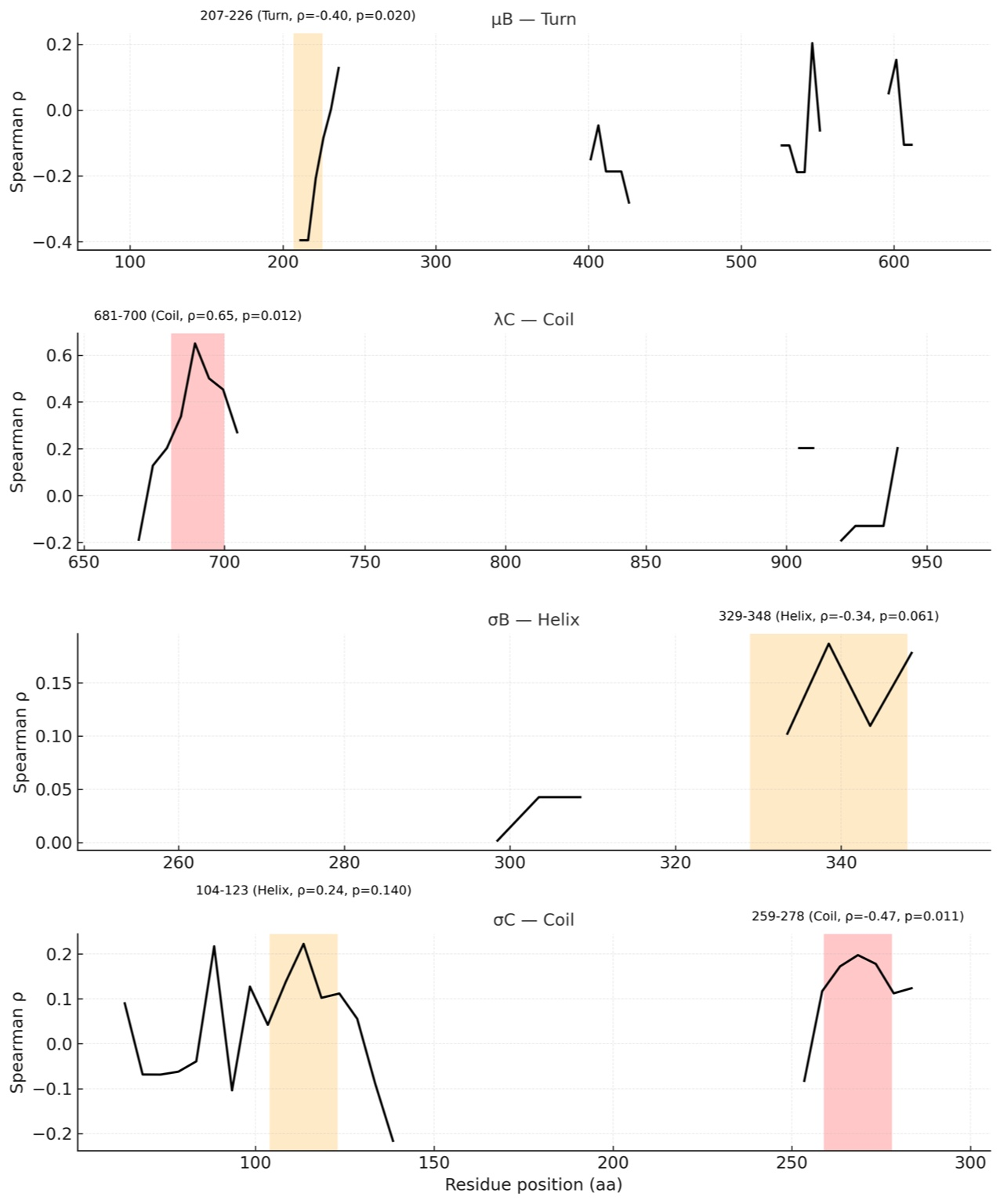


**Figure S6.** **Sliding-window correlations across μB, λC, σB, and σC proteins.**

Sliding-window correlation scan across was carried out for each protein, with windows of 10, 15, 20, and 25 amino acids and a stride of 5 residues between windows to warrant coverage. For each window, the mean fractional content of α-helix, β-strand, coil, and turn elements was calculated (defined as secondary-structure features) using established secondary-structure or predicted states where structural models were unavailable. The resulting window-specific structural profiles were then correlated with virulence clusters using Spearman’s rank correlation (ρ). Primary hotspots shaded red (annotated with position, feature, ρ, and p); secondary hotspots shaded orange.

(a)


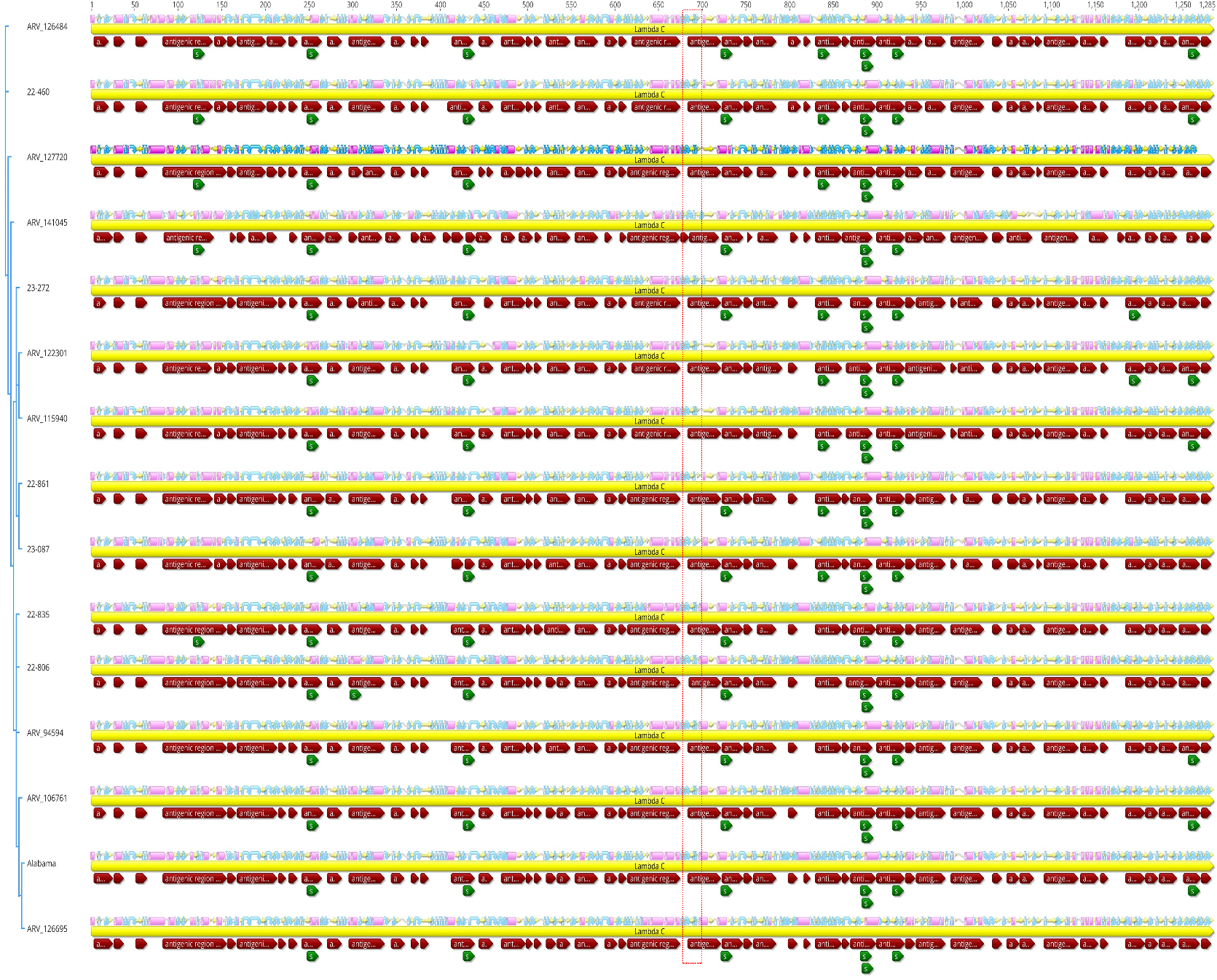


(b)


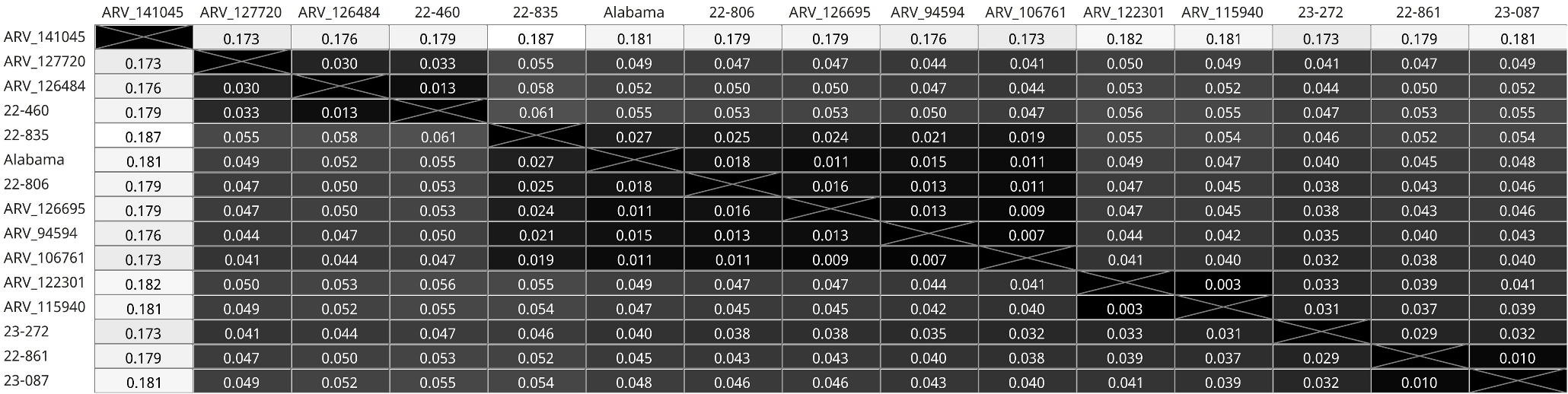


(c)


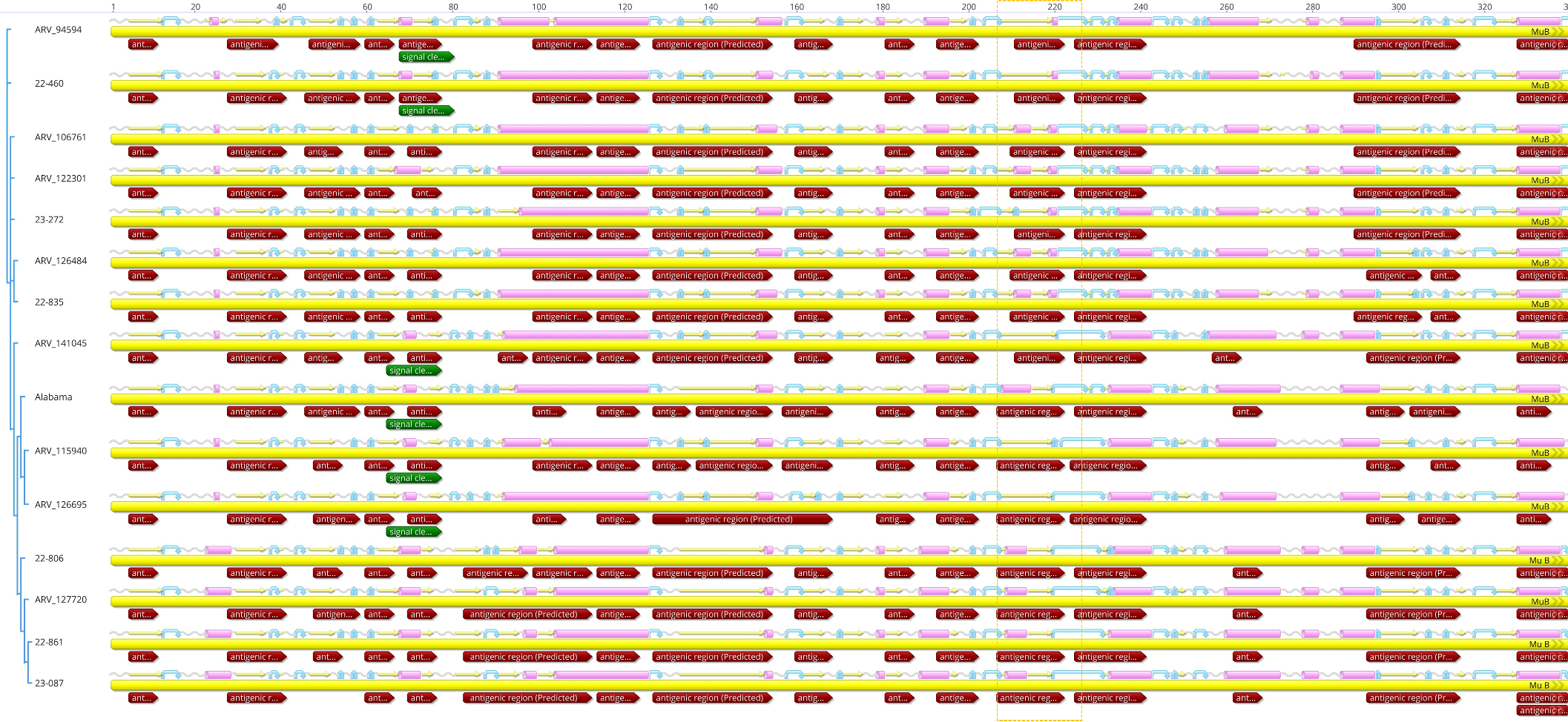


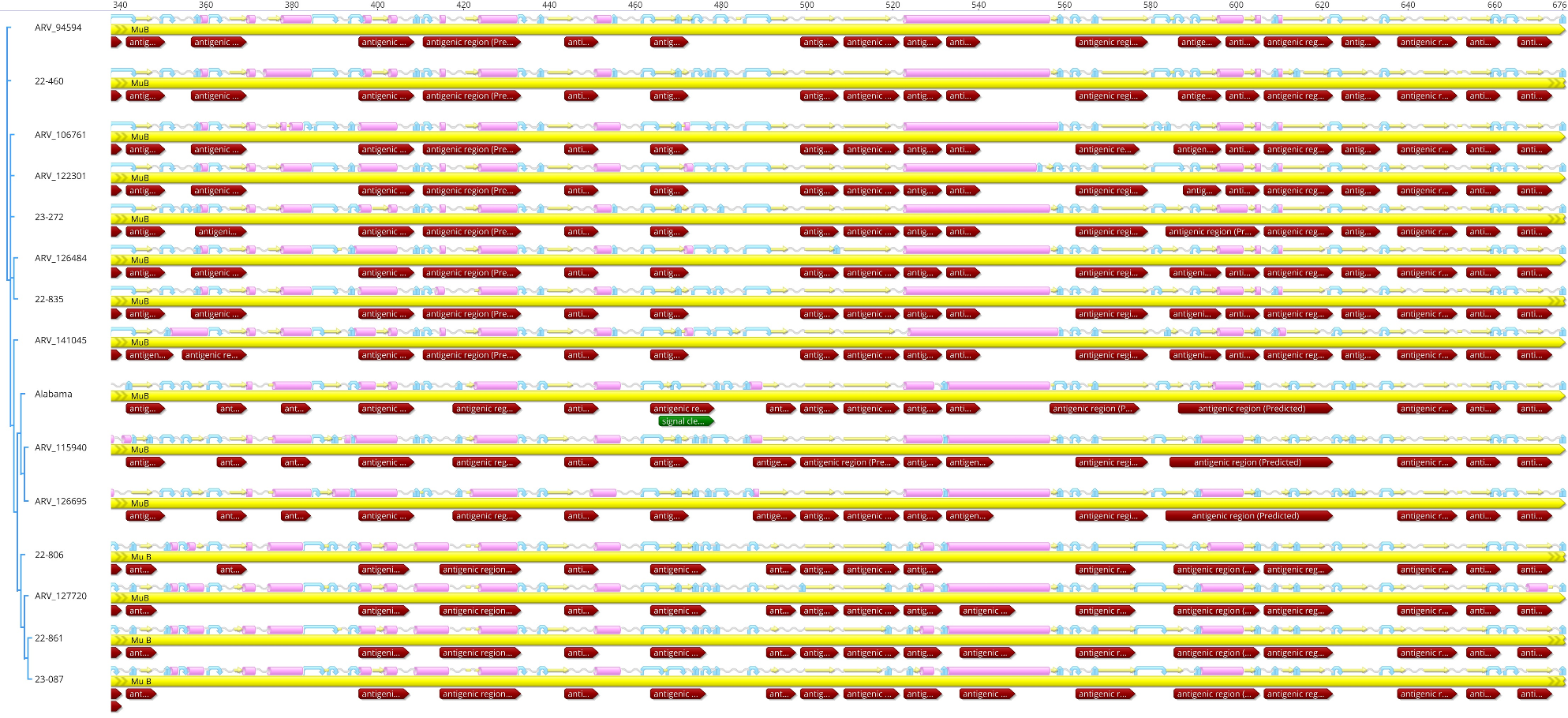


(d)


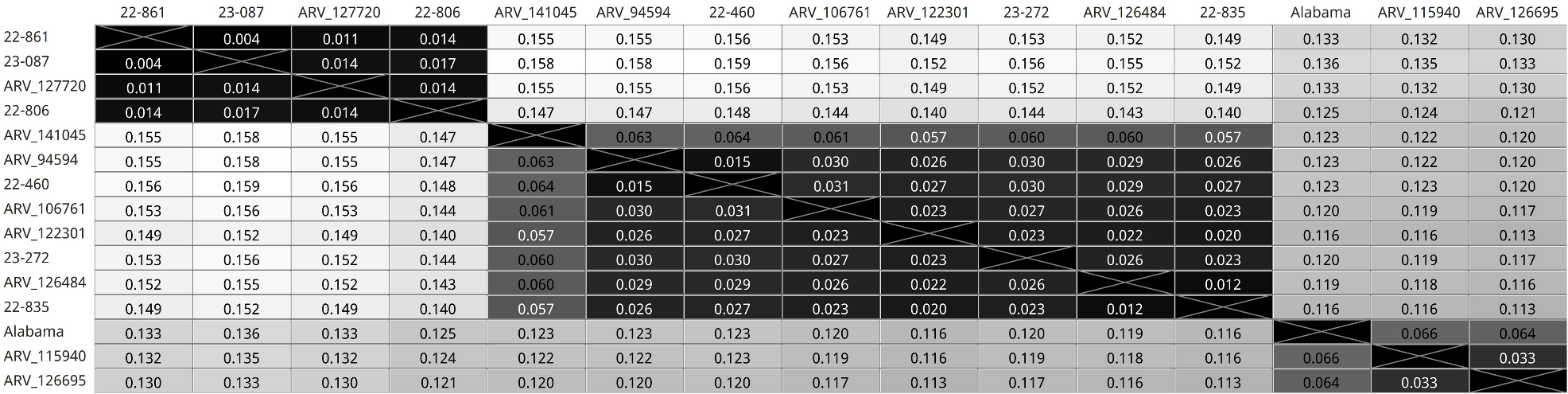


**Figure S7. Amino acid sequence analysis and secondary structure prediction of (a) λC, and (c) μB of our ARV strains and the corresponding heatmaps of (b) λC and (d) μB denoting the patristic distances.** The red dashed box in the λC refers to the potential candidate region in discrimination capacity, whereas the orange dashed box in μB refers to the secondary candidate region.


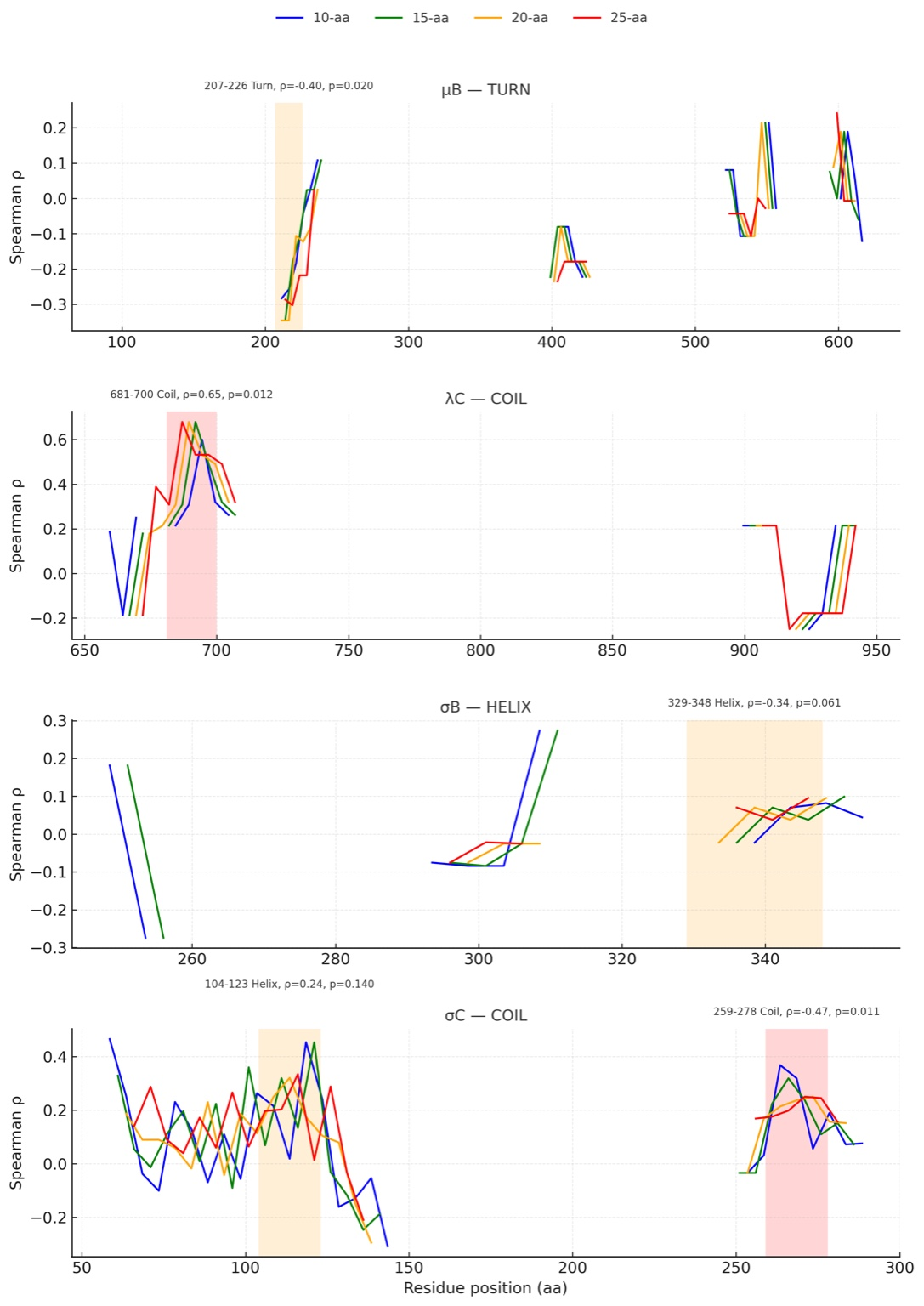


**Figure S8.** **Primary and Secondary Hotspots from multiple Sliding-Window analyses (windows 15-25, 5 aa stride).** Primary hotspots shaded red (annotated with position, feature, Spearman’s rank correlation (ρ), and p); secondary hotspots shaded orange.
