# Supplementary material for: Novel multigene molecular characterization of avian reovirus strains and associated embryonic pathogenicity
Source: J Virol. 2026 May 5;100(6):e01982-25. doi: 10.1128/jvi.01982-25 (PMC13288925; doi:10.1128/jvi.01982-25)
Supplement: Supplemental tables — Tables S1 to S8. [file jvi.01982-25-s0002.docx]

**Table S1. List of genomes used for phylogenetic analysis for defining ARV outer protein-based host tropism**

| Accession numbers | | | | Isolate name | Origin | Date | Host |
| --- | --- | --- | --- | --- | --- | --- | --- |
| L3 | M2 | S1 | S3 |  |  |  |  |
| KC865788 | KC865790 | KC865792 | KC865794 | T1781 | Hungary | 2012 | Chicken |
| MW394458 | MW394460 | MW394462 | MW394464 | SDYT2020 | China | 2020 | Chicken |
| KF741758 | KF741760 | KF741762 | KF741764 | S1133 | USA | 1973 | Chicken |
| KU169290 | KU169292 | KU169294 | KU169296 | Reo/PA/Layer/27614/13 | USA | 2013 | Chicken |
| KT428310 | KT428312 | KT428314 | KT428316 | Reo/PA/Layer/01224B/14 | USA | 2014 | Chicken |
| KT428300 | KT428302 | KT428304 | KT428306 | Reo/PA/Layer/01224A/14 | USA | 2014 | Chicken |
| MW174786 | MW174788 | MW174790 | MW174792 | PHC-2020-0545 | China | 2020 | Chicken |
| MN879656 | MN879658 | MN879660 | MN879662 | D7 | Canada | 2014 | Chicken |
| MN879636 | MN879638 | MN879640 | MN879642 | D5 | Canada | 2014 | Chicken |
| MN879696 | MN879698 | MN879700 | MN879702 | D11 | Canada | 2014 | Chicken |
| MN879686 | MN879688 | MN879690 | MN879692 | D10 | Canada | 2014 | Chicken |
| MN879596 | MN879598 | MN879600 | MN879602 | D1 | Canada | 2014 | Chicken |
| OK077995 | OK077997 | OK077999 | OK078003 | AHZJ19 | China | 2019 | Chicken |
| KX398264 | KX398266 | KX398268 | KX398270 | 924-Bi-05 | Hungary | 2005 | Chicken |
| KX398274 | KX398276 | KX398278 | KX398280 | 3211-V-02 | Hungary | 2002 | Chicken |
| MW244844 | MW244846 | MW244848 | MW244850 | V-ARV-SD26 | China | 2020 | Chicken |
| MK583333 | MK583335 | MK583337 | MK583339 | K1600657 | USA | 2016 | Chicken |
| KF741728 | KF741730 | KF741732 | KF741734 | GuangxiR2 | China | 2000 | Chicken |
| KP173685 | KP173687 | KP173689 | KP173691 | Reo/PA/Turkey/22342/13 | USA | 2013 | Turkey |
| KR997911 | KR997913 | KR997915 | KR997917 | D1246 | Hungary | 2009 | Turkey |
| KR997921 | KR997923 | KR997925 | KR997927 | D1104 | Hungary | 2009 | Turkey |
| KR997901 | KR997903 | KR997905 | KR997907 | 19831M09 | Hungary | 2009 | Turkey |
| OP598204 | OP598200 | OP598198 | OP598196 | JS2022 | China | 2003 | Goose |
| MK955820 | MK955822 | MK955827 | MK955825 | GRV-GD2020 | China | 2020 | Goose |
| MG869810 | MG869813 | MG869815 | MG869817 | Goose_ReoSDPY1116-17_China | China | 2017 | Goose |
| KF809664 | KF809666 | KF809668 | KF809670 | D20/99 | Hungary | 1999 | Goose |
| JX145330 | JX145332 | JX145334 | JX145336 | 03G | China | 2003 | Goose |
| KF306084 | KF306086 | KF306088 | KF306090 | ZJ2000M | China | 2011 | Duck |
| KF154112 | KF154114 | KF154116 | KF154118 | ZJ00M | China | 2000 | Duck |
| MZ733731 | MZ733727 | MZ733722 | MZ733724 | YL | China | 2020 | Duck |
| KC493574 | JX440514 | KC493571 | JX826588 | TH11 | China | 2011 | Duck |
| MK955820 | MK955822 | MK955827 | MK955825 | SY | China | 2018 | Duck |
| MH510247 | MH510249 | MH510251 | MH510253 | SH12 | China | 2012 | Duck |
| KJ879926 | KJ879928 | KJ879930 | KJ879932 | SD-12 | China | 2012 | Duck |
| MZ736883 | MZ736879 | MZ736874 | MZ736876 | QR | China | 2020 | Duck |
| MK749400 | MK749402 | MK749407 | MK749405 | N-DRV-XT18 | China | 2018 | Duck |
| MT829222 | MT829220 | MT829218 | MT829216 | NDRV-SD19/6202 | China | 2019 | Duck |
| MT829212 | MT829210 | MT829208 | MT829206 | NDRV-SD19/6201 | China | 2019 | Duck |
| ON040909 | ON040911 | ON040913 | ON040915 | N-DRV-LY20 | China | 2021 | Duck |
| JX478262 | JX478264 | JX478266 | JX478268 | J18 | China | 2008 | Duck |
| KT861589 | KT861591 | KT861593 | KT861595 | HN5d | China | 2013 | Duck |
| MN747006 | MN747008 | MN747010 | MN747012 | GX-Y7/DRV/CHN/2018 | China | 2018 | Duck |
| MW388676 | MW388678 | MW388680 | MW388682 | G018-19/DRV/AUS/2019 | Australia | 2019 | Duck |
| MH510257 | MH510259 | MH510261 | MH510263 | DH13 | China | 2013 | Duck |
| MT93953 | MT939505 | MT939507 | MT939509 | DE150 | China | 2020 | Duck |
| KJ871019 | KJ871021 | KJ871023 | KJ871025 | D1546 | France | 2010 | Duck |
| KC508649 | KC508651 | KC508653 | KC508655 | 815-12 | China | 2010 | Duck |
| JX478252 | JX478254 | JX478256 | JX478258 | 91 | China | 2009 | Duck |
| KJ865892 | KJ874301 | - | - | TERV-MN6 | USA | 2011 | Turkey |
| KJ865891 | KJ874297 | - | - | TERV-MN2 | USA | 2010 | Turkey |
| KJ865890 | KJ874296 | KJ700479 | - | TERV-MN1 | USA | 2011 | Turkey |
| KJ865889 | KJ874295 | - | - | TARV-MN13 | USA | 2014 | Turkey |
| KJ865888 | KJ874294 | - | - | TARV-MN12 | USA | 2013 | Turkey |
| KJ865887 | KJ874293 | - | - | TARV-MN11 | USA | 2013 | Turkey |
| KJ865886 | KJ874292 | KF872242 | KF872262 | TARV-MN10 | USA | 2013 | Turkey |
| KJ865885 | KJ874291 | KF872241 | - | TARV-MN9 | USA | 2013 | Turkey |
| KJ865884 | KJ874286 | KF872235 | - | TARV-MN4 | USA | 2011 | Turkey |
| KJ865883 | KJ874284 | KF872233 | - | TARV-MN2 | USA | 2011 | Turkey |
| - | KJ874303 | - | - | TERV-MN8 | USA | 2012 | Turkey |
| - | KJ874302 | - | - | TERV-MN7 | USA | 2012 | Turkey |
| - | KJ874300 | - | - | TERV-MN5 | USA | 2011 | Turkey |
| - | KJ874299 | - | - | TERV-MN4 | USA | 2011 | Turkey |
| - | KJ874298 | - | - | TERV-MN3 | USA | 2011 | Turkey |
| - | KJ874290 | KF872240 | - | TARV-MN8 | USA | 2013 | Turkey |
| - | KJ874289 | KF872239 | - | TARV-MN7 | USA | 2012 | Turkey |
| - | KJ874288 | KF872237 | - | TARV-MN6 | USA | 2011 | Turkey |
| - | KJ874287 | KF872236 | - | TARV-MN5 | USA | 2011 | Turkey |
| - | KJ874285 | KF872234 | - | TARV-MN3 | USA | 2011 | Turkey |
| - | KJ874283 | KF872232 | - | TARV-MN1 | USA | 2011 | Turkey |
| - | KJ874282 | KF872231 | - | TARV-O'Neil | USA | 2011 | Turkey |
| - | KJ874281 | KF872238 | - | TARV_Crestview | USA | 2011 | Turkey |
| - | - | DQ995806 | AF465799 | NC98 | USA | 1998 | Turkey |
| - | - | - | AY444910 | TX99 | USA | 1999 | Turkey |
| - | - | - | AY444911 | TX98 | USA | 1998 | Turkey |
| - | - | - | AY444912 | ATCC VR-818 | - | - | Turkey |
| - | - | - | AY444913 | PEMS85 | USA | 1985 | Turkey |
| - | - | - | KC683824 | TReoV1 | Brazil | 2012 | Turkey |
| - | - | - | KC683825 | TReoV2 | Brazil | 2012 | Turkey |
| - | - | - | KF183931 | D-AB15 | USA | 2011 | Turkey |
| - | - | - | KF183932 | D-AB14 | USA | 2011 | Turkey |
| - | - | DQ525419 | - | R44 | USA | 2003 | Turkey |
| PP657717 | PP657719 | PP657714 | PP657765 | TRV-S2 | Germany | 2004 | 2004 |
| PP657723 | - | - | PP657768 | TRV-S4 | Germany | 2005 | 2005 |
| PP657728 | PP657730 | PP657725 | PP657771 | TRV-S5 | Germany | 2005 | 2005 |
| - | PP657736 | PP657732 | PP657775 | TRV-S7 | Germany | 2007 | 2007 |
| PP657741 | - | PP657738 | PP657777 | TRV-S8 | Germany | 2007 | 2007 |
| PP657747 | PP657749 | PP657744 | PP657780 | TRV-S9 | Germany | 2007 | 2007 |
| PP657761 | - | PP657758 | PP657785 | TRV-S11 | Germany | 2007 | 2007 |
| PP657755 | - | PP657752 | PP657788 | TRV-S12 | Germany | 2007 | 2007 |
| PP657791 | PP657794 | - | PP657797 | TRV-S14 | Germany | 2008 | 2008 |
| PP657800 | PP657803 | PP657805 | PP657807 | TRV-S16 | Germany | 2008 | 2008 |
| PP657810 | PP657813 | PP657815 | PP657817 | TRV-S17 | Germany | 2008 | 2008 |
| PP657821 | PP657823 | PP657825 | PP657827 | TRV-S18 | Germany | 2008 | 2008 |
| PP657833 | - | - | PP657830 | TRV-S19 | Germany | 2008 | 2008 |
| PP657839 | - | PP657842 | PP657844 | TRV-S20 | Germany | 2008 | 2008 |
| PP657848 | PP657850 | - | PP657853 | TRV-S21 | Germany | 2008 | 2008 |

**Table S2. List of Chicken isolates used for phylogenetic analysis for multi-gene based genotyping comparison**

| Accession numbers | | | | Isolate name | Origin | Date |
| --- | --- | --- | --- | --- | --- | --- |
| L3 | M2 | S1 | S3 |  |  |  |
| MN879656 | MN879658 | MN87960 | MN87962 | D7 | Canada | 2014 |
| MW174786 | MW174788 | MW174790 | MW174792 | PHC-2020-0545 | China | 2020 |
| MN879636 | MN879638 | MN879640 | MN879642 | D5 | Canada | 2014 |
| MN879706 | MN879708 | MN879710 | MN879712 | D12 | Canada | 2014 |
| MN879676 | MN879678 | MN879680 | MN879682 | D9 | Canada | 2013 |
| MN879666 | MN879668 | MN879670 | MN879672 | D8 | Canada | 2014 |
| MN879646 | MN879648 | MN879650 | MN879652 | D6 | Canada | 2014 |
| MF183214 | MF183216 | MF183218 | MF183220 | LY383 | China | 2016 |
| MW244844 | MW244846 | MW244848 | MW244850 | V-ARV-SD26 | China | 2020 |
| MN879616 | MN879618 | MN879620 | MN879622 | D3 | Canada | 2014 |
| MN879596 | MN879598 | MN879600 | MN879602 | D1 | Canada | 2014 |
| KT428310 | KT428312 | KT428314 | KT428316 | Reo/PA/Layer/01224B/14 | USA | 2014 |
| MN879686 | MN879688 | MN879690 | MN879692 | D10 | Canada | 2014 |
| KX398274 | KX398276 | KX398278 | KX398280 | 3211-V-02 | Hungary | 2002 |
| KX398264 | KX398266 | KX398268 | KX398270 | 924-Bi-05 | Hungary | 2005 |
| NC_015128 | NC_015130 | NC_015132 | NC_015134 | AVS-B | USA | 2006 |
| KM877327 | KM877329 | KM877331 | KM877333 | Reo/PA/Broiler/05682/12 | USA | 2012 |
| MN879696 | MN879698 | MN879700 | MN879702 | D11 | Canada | 2014 |
| MN879626 | MN879628 | MN879630 | MN879632 | D4 | Canada | 2014 |
| KX398314 | KX398316 | KX398318 | KX398320 | 17203-M-06 | Hungary | 2006 |
| MK583333 | MK583335 | MK583337 | MK583339 | K1600657 | USA | 2016 |
| MW394458 | MW394460 | MW394462 | MW394464 | SDYT2020 | China | 2020 |
| OK077995 | OK077997 | OK077999 | OK078003 | AHZJ19 | China | 2019 |
| KX398304 | KX398306 | KX398308 | KX398310 | 16821-M-06 | Hungary | 2006 |
| KT428300 | KT428302 | KT428304 | KT428306 | Reo/PA/Layer/01224A/14 | USA | 2014 |
| MK416135 | MK416137 | MK416139 | MK416141 | K1600600 | USA | 2016 |
| MW002449 | MW002451 | MW002453 | MW002455 | Reo/Ck/TX/115940 | USA | 2016 |
| KY860635 | KY860637 | KY860639 | KY860641 | MS01 | China | 2017 |
| KF741698 | KF741700 | KF741702 | KF741704 | 526 | China | 2013 |
| KJ476701 | KJ476703 | KJ476705 | KJ476707 | GX/2010/1 | China | 2010 |
| KF741718 | KF741720 | KF741722 | KF741724 | C78 | China | 2013 |
| KF741708 | KF741710 | KF741712 | KF741714 | 1733 | USA | 1997 |
| MK616645 | MK616647 | MK616649 | MK616651 | T1502036 | USA | 2015 |
| KX398244 | KX398246 | KX398248 | KX3982450 | 875-Bi-05 | Hungary | 2005 |
| KU169290 | KU169292 | KU169294 | KU169296 | Reo/PA/Layer/27614/13 | USA | 2013 |
| KF741758 | KF741760 | KF741762 | KF741764 | S1133 | USA | 1973 |
| KF741748 | KF741750 | KF741752 | KF741754 | GX110116 | China | 2011 |
| KF741738 | KF741740 | KF741742 | KF741744 | GX110058 | China | 2011 |
| KF741728 | KF741730 | KF741732 | KF741734 | GuangxiR2 | China | 2000 |
| EU616738 | EU616742 | EF057398 | - | T-98 | China | 2006 |
| EU616737 | EU616741 | EF057397 | - | C-98 | China | 2006 |
| KX398294 | KX398296 | KX398298 | KX398300 | 4599-V-04 | Hungary | 2004 |
| KX398324 | KX398326 | KX398328 | KX398330 | 17227-M-10 | Hungary | 2010 |
| MN879606 | MN879608 | MN879610 | MN879612 | D2 | Canada | 2014 |
| KX398234 | KX398236 | KX398238 | KX398240 | 284-V-06 | Hungary | 2006 |
|  |  |  |  | ARV_127720 | USA |  |
| OR612107 | OR612109 | OR612111 | OR612113 | ARV_141045 | USA | 2021 |
| OR270112 | OR270114 | OR270116 | OR270118 | ARV_126484 | USA | 2018 |
| OR612135 | OR612137 | OR612139 | OR612141 | ARV_94594 | USA | 2012 |
| KP731613 | KP731615 | KP731617 | KP731619 | Reo/PA/Broiler/15511/13 | USA | 2014 |
| OR612125 | OR612127 | OR612129 | OR612131 | ARV_Alabama | USA | 2021 |

**Table S3. Pairwise log-rank tests with Benjamini-Hochberg p-value adjustment to maintain false discovery rate at 0.05.**

|  | **Control** | **22-861** | **ARV-94584** | **23-272** | **ARV-126695** | **Alabama** | **ARV-106761** | **ARV-126484** | **ARV-127720** | **ARV-115940** | **ARV-122301** | **22-835** | **S1133** | **ARV-141045** | **2177** | **23-087** | **22-460** |
| --- | --- | --- | --- | --- | --- | --- | --- | --- | --- | --- | --- | --- | --- | --- | --- | --- | --- |
| 22-861 | 0.081 |  |  |  |  |  |  |  |  |  |  |  |  |  |  |  |  |
| ARV-94584 | **0.001** | 0.029 |  |  |  |  |  |  |  |  |  |  |  |  |  |  |  |
| 23-272 | **0.001** | **0.010** | 0.161 |  |  |  |  |  |  |  |  |  |  |  |  |  |  |
| ARV-126695 | **0.001** | **0.006** | 0.089 | 0.863 |  |  |  |  |  |  |  |  |  |  |  |  |  |
| Alabama | **0.001** | **0.006** | 0.074 | 0.717 | 0.814 |  |  |  |  |  |  |  |  |  |  |  |  |
| ARV-106761 | **0.001** | **0.002** | **0.015** | 0.163 | 0.142 | 0.240 |  |  |  |  |  |  |  |  |  |  |  |
| ARV-126484 | **0.001** | **0.001** | **0.002** | **0.014** | **0.010** | **0.019** | 0.163 |  |  |  |  |  |  |  |  |  |  |
| ARV-127720 | **0.001** | **0.001** | **0.003** | **0.031** | **0.024** | **0.045** | 0.287 | 0.863 |  |  |  |  |  |  |  |  |  |
| ARV-115940 | **0.001** | **0.001** | **0.003** | **0.008** | **0.003** | **0.008** | 0.116 | 0.863 | 0.672 |  |  |  |  |  |  |  |  |
| ARV-122301 | **0.001** | **0.001** | **0.001** | **0.010** | **0.008** | **0.013** | 0.086 | 0.554 | 0.458 | 0.621 |  |  |  |  |  |  |  |
| 22-835 | **0.001** | **0.001** | **0.001** | **0.001** | **0.001** | **0.001** | **0.002** | **0.026** | 0.060 | **0.034** | 0.508 |  |  |  |  |  |  |
| S1133 | **0.001** | **0.001** | **0.001** | **0.001** | **0.001** | **0.001** | **0.002** | **0.032** | 0.061 | **0.036** | 0.330 | 0.660 |  |  |  |  |  |
| ARV-141045 | **0.001** | **0.001** | **0.001** | **0.001** | **0.001** | **0.001** | **0.001** | **0.004** | **0.012** | **0.012** | 0.280 | 0.118 | 0.621 |  |  |  |  |
| 2177 | **0.001** | **0.001** | **0.001** | **0.001** | **0.001** | **0.001** | **0.001** | **0.004** | **0.010** | **0.010** | 0.191 | 0.074 | 0.435 | 0.588 |  |  |  |
| 23-087 | **0.001** | **0.001** | **0.001** | **0.001** | **0.001** | **0.001** | **0.001** | **0.009** | **0.018** | **0.012** | 0.177 | 0.177 | 0.550 | 0.724 | 0.964 |  |  |
| 22-460 | **0.001** | **0.001** | **0.001** | **0.001** | **0.001** | **0.001** | **0.002** | **0.019** | **0.031** | **0.018** | 0.175 | 0.264 | 0.660 | 0.695 | 0.962 | 1.000 |  |
| 22-806 | **0.001** | **0.001** | **0.001** | **0.001** | **0.001** | **0.001** | **0.001** | **0.001** | **0.002** | **0.002** | **0.034** | **0.004** | 0.060 | **0.017** | 0.053 | 0.121 | 0.248 |

Significant values (p < 0.05) are shown in bold text

**Table S4: Evolutionary divergence estimates between σC sequences of ARV isolates.**

| **Species 1** | **Species 2** | **Distance (*d*)** |
| --- | --- | --- |
| **ARV 141045** | **C78** | **0.00000100** |
| T-98 | C-98 | 0.00000100 |
| T-98 | MS01 | 0.00000100 |
| C-98 | MS01 | 0.00000100 |
| K1502030 | T1502036 | 0.00000100 |
| 875-Bi-05 | 878-Bi-05 | 0.00000100 |
| V-ARV-SD26 | LY383 | 0.00000100 |
| D12 | D6 | 0.00000100 |
| ARV 141045 | GX/2010/1 | 0.00278951 |
| C78 | GX/2010/1 | 0.00278951 |
| **D12** | **ARV 94594** | **0.00285941** |
| **D6** | **ARV 94594** | **0.00285941** |
| ARV 94594 | **22-460** | **0.00286398** |
| GX110116 | ARV 141045 | 0.00559802 |
| GX110116 | C78 | 0.00559802 |
| **ARV_115940** | **117816** | **0.00561992** |
| D12 | Reo/PA/Layer/01224B/14 | 0.00572585 |
| D6 | Reo/PA/Layer/01224B/14 | 0.00572585 |
| D12 | 22-460 | 0.00573464 |
| D6 | 22-460 | 0.00573464 |
| Reo/PA/Broiler/15511/13 | D6 | 0.00573980 |
| D12 | Reo/PA/Broiler/15511/13 | 0.00573980 |
| T-98 | GuangxiR2 | 0.00839895 |
| C-98 | GuangxiR2 | 0.00839895 |
| MS01 | GuangxiR2 | 0.00839895 |
| GX110116 | GX/2010/1 | 0.00839916 |
| 1733 | GuangxiR2 | 0.00842499 |
| GX110058 | S1133 | 0.00843856 |
| ARV 94594 | Reo/PA/Layer/01224B/14 | 0.00859837 |
| D12 | D8 | 0.00860580 |
| D6 | D8 | 0.00860580 |
| Reo/PA/Broiler/15511/13 | ARV 94594 | 0.00861934 |
| T-98 | 1733 | 0.01125395 |
| C-98 | 1733 | 0.01125395 |
| MS01 | 1733 | 0.01125395 |
| ARV 94594 | D8 | 0.01148727 |
| Reo/PA/Layer/01224B/14 | 22-460 | 0.01149630 |
| Reo/PA/Broiler/15511/13 | Reo/PA/Layer/01224B/14 | 0.01150667 |
| Reo/PA/Broiler/15511/13 | 22-460 | 0.01152441 |
| ARV 141045 | T-98 | 0.01407948 |
| C78 | T-98 | 0.01407948 |
| ARV 141045 | C-98 | 0.01407948 |
| C78 | C-98 | 0.01407948 |
| ARV 141045 | MS01 | 0.01407948 |
| C78 | MS01 | 0.01407948 |
| Reo/PA/Layer/01224B/14 | D8 | 0.01437687 |
| D8 | 22-460 | 0.01439902 |
| Reo/PA/Broiler/15511/13 | D8 | 0.01441203 |
| GX/2010/1 | T-98 | 0.01689963 |
| GX/2010/1 | C-98 | 0.01689963 |
| GX/2010/1 | MS01 | 0.01689963 |
| ARV 141045 | GuangxiR2 | 0.01691532 |
| C78 | GuangxiR2 | 0.01691532 |
| S1133 | T-98 | 0.01692487 |
| S1133 | C-98 | 0.01692487 |
| S1133 | MS01 | 0.01692487 |
| GX/2010/1 | GuangxiR2 | 0.01973958 |
| S1133 | GuangxiR2 | 0.01976923 |
| GX110116 | T-98 | 0.01978444 |
| GX110116 | C-98 | 0.01978444 |
| GX110116 | MS01 | 0.01978444 |
| GX110058 | T-98 | 0.01979417 |
| GX110058 | C-98 | 0.01979417 |
| GX110058 | MS01 | 0.01979417 |
| ARV 141045 | 1733 | 0.01983291 |
| C78 | 1733 | 0.01983291 |
| D12 | D1 | 0.02021920 |
| D6 | D1 | 0.02021920 |
| GX110116 | GuangxiR2 | 0.02263785 |
| GX110058 | GuangxiR2 | 0.02264902 |
| GX/2010/1 | 1733 | 0.02267207 |
| S1133 | 1733 | 0.02270646 |
| ARV 94594 | D1 | 0.02313379 |
| S1133 | ARV 141045 | 0.02556617 |
| S1133 | C78 | 0.02556617 |
| GX110116 | 1733 | 0.02559531 |
| GX110058 | 1733 | 0.02560803 |
| Reo/PA/Layer/01224B/14 | D1 | 0.02605805 |
| D1 | 22-460 | 0.02609837 |
| Reo/PA/Broiler/15511/13 | D1 | 0.02612210 |
| S1133 | GX/2010/1 | 0.02841422 |
| GX110058 | ARV 141045 | 0.02847703 |
| GX110058 | C78 | 0.02847703 |
| 17227-M-10 | 4599-V-04 | 0.02847773 |
| D1 | D8 | 0.02901199 |
| GX110058 | GX/2010/1 | 0.03133287 |
| S1133 | GX110116 | 0.03136560 |
| **ARV 126695** | **ARV 127720** | **0.03240669** |
| **22-806** | **K1600600** | **0.03420361** |
| GX110058 | GX110116 | 0.03430204 |
| **22-861** | ARV 94594 | **0.03805098** |
| 22-861 | D12 | 0.03817892 |
| 22-861 | D6 | 0.03817892 |
| D4 | D11 | 0.03824956 |
| Reo/PA/Layer/27614/13 | 875-Bi-05 | 0.03999953 |
| Reo/PA/Layer/27614/13 | 878-Bi-05 | 0.03999953 |
| V-ARV-SD26 | D12 | 0.04082729 |
| LY383 | D12 | 0.04082729 |
| V-ARV-SD26 | D6 | 0.04082729 |
| LY383 | D6 | 0.04082729 |
| 22-861 | 22-460 | 0.04109280 |
| V-ARV-SD26 | ARV 94594 | 0.04129957 |
| LY383 | ARV 94594 | 0.04129957 |
| 22-861 | Reo/PA/Layer/01224B/14 | 0.04415593 |
| 22-861 | Reo/PA/Broiler/15511/13 | 0.04426587 |
| V-ARV-SD26 | Reo/PA/Broiler/15511/13 | 0.04436836 |
| LY383 | Reo/PA/Broiler/15511/13 | 0.04436836 |
| V-ARV-SD26 | 22-460 | 0.04436978 |
| LY383 | 22-460 | 0.04436978 |
| **Alabama** | **AVS-B** | **0.04475798** |
| K1600600 | 875-Bi-05 | 0.04574858 |
| K1600600 | 878-Bi-05 | 0.04574858 |
| V-ARV-SD26 | Reo/PA/Layer/01224B/14 | 0.04677130 |
| LY383 | Reo/PA/Layer/01224B/14 | 0.04677130 |
| 22-861 | D8 | 0.04719505 |
| K1600600 | Reo/PA/Layer/27614/13 | 0.04887763 |
| V-ARV-SD26 | D8 | 0.04979591 |
| LY383 | D8 | 0.04979591 |
| 22-861 | V-ARV-SD26 | 0.05305120 |
| 22-861 | LY383 | 0.05305120 |
| **22-835** | **K1600657** | **0.05665549** |
| K1502030 | 875-Bi-05 | 0.05812514 |
| T1502036 | 875-Bi-05 | 0.05812514 |
| K1502030 | 878-Bi-05 | 0.05812514 |
| T1502036 | 878-Bi-05 | 0.05812514 |
| 22-861 | D1 | 0.05940786 |
| V-ARV-SD26 | D1 | 0.06195020 |
| LY383 | D1 | 0.06195020 |
| 117816 | 875-Bi-05 | 0.06686462 |
| 117816 | 878-Bi-05 | 0.06686462 |
| ARV 115940 | 875-Bi-05 | 0.06693613 |
| Reo/Ck/TX/115940 | 875-Bi-05 | 0.06693613 |
| ARV 115940 | 878-Bi-05 | 0.06693613 |
| Reo/Ck/TX/115940 | 878-Bi-05 | 0.06693613 |
| 22-806 | 875-Bi-05 | 0.06724349 |
| 22-806 | 878-Bi-05 | 0.06724349 |
| 16821-M-06 | 3457-M-11 | 0.06856801 |
| Reo/PA/Broiler/05682/12 | AVS-B | 0.06929905 |
| 22-806 | Reo/PA/Layer/27614/13 | 0.07315094 |
| K1502030 | Reo/PA/Layer/27614/13 | 0.07949212 |
| T1502036 | Reo/PA/Layer/27614/13 | 0.07949212 |
| **ARV 122301** | **D10** | **0.08023499** |
| 117816 | K1502030 | 0.09095434 |
| 117816 | T1502036 | 0.09095434 |
| ARV 115940 | K1502030 | 0.09105140 |
| Reo/Ck/TX/115940 | K1502030 | 0.09105140 |
| ARV 115940 | T1502036 | 0.09105140 |
| Reo/Ck/TX/115940 | T1502036 | 0.09105140 |
| 117816 | Reo/PA/Layer/27614/13 | 0.09168514 |
| ARV 115940 | Reo/PA/Layer/27614/13 | 0.09178369 |
| Reo/Ck/TX/115940 | Reo/PA/Layer/27614/13 | 0.09178369 |
| **ARV 126484** | **526** | **0.09449747** |
| K1502030 | K1600600 | 0.09489290 |
| T1502036 | K1600600 | 0.09489290 |
| 117816 | K1600600 | 0.09489959 |
| ARV 115940 | K1600600 | 0.09500167 |
| Reo/Ck/TX/115940 | K1600600 | 0.09500167 |
| **23-272** | **Alabama** | **0.09587338** |
| Reo/PA/Broiler/05682/12 | Alabama | 0.10475995 |
| K1502030 | 22-806 | 0.10509969 |
| T1502036 | 22-806 | 0.10509969 |
| 23-272 | 22-835 | 0.10549434 |
| 17203-M-06 | D4 | 0.10586664 |
| 17203-M-06 | D11 | 0.10713135 |
| **23-087** | 22-835 | **0.10806116** |
| 117816 | 22-806 | 0.10811508 |
| ARV 115940 | 22-806 | 0.10823191 |
| Reo/Ck/TX/115940 | 22-806 | 0.10823191 |
| 23-087 | K1600657 | 0.11110260 |
| AVS-B | K1600657 | 0.11117127 |
| 875-Bi-05 | 526 | 0.11159963 |
| 878-Bi-05 | 526 | 0.11159963 |
| 23-272 | AVS-B | 0.11229238 |
| Reo/PA/Broiler/05682/12 | K1600657 | 0.11258083 |
| **ARV 106761** | **D7** | **0.11499071** |
| K1502030 | 526 | 0.11837055 |
| T1502036 | 526 | 0.11837055 |

The strains displaying the closest evolutionary distance to our isolates, are shown in bold red. The evolutionary distance is shown up to d = 0.11837055.

**Table S5: Evolutionary divergence estimates between λC sequences of ARV isolates.**

| **Species 1** | **Species 2** | **Distance (*d*)** |
| --- | --- | --- |
| D6 | D12 | 0.00000100 |
| D6 | D9 | 0.00000100 |
| D12 | D9 | 0.00000100 |
| 1733 | MS01 | 0.00076656 |
| T-98 | MS01 | 0.00076659 |
| K1502030 | T1502036 | 0.00153106 |
| C-98 | T-98 | 0.00153347 |
| 1733 | T-98 | 0.00153360 |
| GX110058 | 1733 | 0.00153400 |
| S1133 | MS01 | 0.00153426 |
| **ARV 115940** | **117816** | **0.00153545** |
| C-98 | MS01 | 0.00230112 |
| GX110058 | MS01 | 0.00230142 |
| S1133 | 1733 | 0.00230199 |
| S1133 | T-98 | 0.00230213 |
| **ARV 141045** | **GX/2010/1** | **0.00230291** |
| GuangxiR2 | 1733 | 0.00230336 |
| GuangxiR2 | GX110058 | 0.00230347 |
| V-ARV-SD26 | LY383 | 0.00230363 |
| C-98 | 1733 | 0.00306897 |
| GX110058 | T-98 | 0.00306954 |
| **ARV 122301** | **117816** | **0.00307123** |
| ARV 122301 | ARV 115940 | 0.00307125 |
| GuangxiR2 | MS01 | 0.00307170 |
| **ARV 106761** | **D5** | **0.00383718** |
| ARV 141045 | GX110116 | 0.00383852 |
| S1133 | C-98 | 0.00383909 |
| S1133 | GX110058 | 0.00383956 |
| GuangxiR2 | T-98 | 0.00384086 |
| GX/2010/1 | GX110116 | 0.00384174 |
| **ARV 126484** | **K1600600** | **0.00384204** |
| D8 | ARV 106761 | 0.00460275 |
| C78 | ARV 141045 | 0.00460441 |
| C-98 | GX110058 | 0.00460694 |
| GuangxiR2 | S1133 | 0.00461221 |
| D4 | D10 | 0.00536558 |
| D11 | D8 | 0.00536911 |
| D11 | ARV 106761 | 0.00536953 |
| D8 | D5 | 0.00537592 |
| C78 | GX110116 | 0.00537764 |
| GuangxiR2 | C-98 | 0.00538029 |
| C78 | GX/2010/1 | 0.00538145 |
| D11 | D5 | 0.00614350 |
| MS01 | C78 | 0.00614783 |
| D1 | D3 | 0.00616987 |
| 3457-M-11 | 17227-M-10 | 0.00691021 |
| 1733 | C78 | 0.00691811 |
| T-98 | C78 | 0.00691854 |
| MS01 | GX110116 | 0.00692388 |
| **ARV 94594** | **D5** | **0.00768695** |
| MS01 | ARV 141045 | 0.00768869 |
| S1133 | C78 | 0.00769256 |
| 1733 | GX110116 | 0.00769520 |
| T-98 | GX110116 | 0.00769568 |
| D7 | Reo/PA/Broiler/15511/13 | 0.00844505 |
| ARV 94594 | ARV 106761 | 0.00845398 |
| 1733 | ARV 141045 | 0.00845976 |
| T-98 | ARV 141045 | 0.00846029 |
| C-98 | C78 | 0.00846084 |
| GX110058 | C78 | 0.00846187 |
| K1600600 | Reo/PA/Broiler/15511/13 | 0.00846499 |
| S1133 | GX110116 | 0.00847108 |
| MS01 | GX/2010/1 | 0.00847277 |
| ARV 126484 | Reo/PA/Broiler/15511/13 | 0.00921656 |
| **ARV 126695** | **D8** | **0.00921688** |
| ARV 126695 | ARV 106761 | 0.00921758 |
| **23-087** | **22-861** | **0.00923292** |
| S1133 | ARV 141045 | 0.00923576 |
| C-98 | GX110116 | 0.00924012 |
| GuangxiR2 | C78 | 0.00924062 |
| GX110058 | GX110116 | 0.00924124 |
| 1733 | GX/2010/1 | 0.00924543 |
| T-98 | GX/2010/1 | 0.00924601 |
| ARV 126695 | D11 | 0.00998429 |
| **22-460** | **Reo/PA/Broiler/15511/13** | **0.00999172** |
| ARV 126695 | D5 | 0.00999696 |
| ARV 94594 | D8 | 0.00999829 |
| C-98 | ARV 141045 | 0.01000427 |
| GX110058 | ARV 141045 | 0.01000548 |
| Reo/PA/Layer/01224B/14 | ARV 106761 | 0.01000878 |
| GuangxiR2 | GX110116 | 0.01002165 |
| S1133 | GX/2010/1 | 0.01002341 |
| ARV 94594 | D11 | 0.01076666 |
| **Alabama** | ARV 126695 | **0.01076792** |
| Alabama | D8 | 0.01076900 |
| Alabama | ARV 106761 | 0.01076982 |
| D6 | D4 | 0.01077817 |
| D12 | D4 | 0.01077817 |
| D9 | D4 | 0.01077817 |
| **22-806** | ARV 106761 | **0.01078022** |
| GuangxiR2 | ARV 141045 | 0.01078621 |
| Reo/PA/Layer/01224B/14 | ARV 94594 | 0.01078878 |
| K1600600 | D7 | 0.01078979 |
| Reo/PA/Layer/01224B/14 | D5 | 0.01079083 |
| C-98 | GX/2010/1 | 0.01079321 |
| GX110058 | GX/2010/1 | 0.01079452 |
| Alabama | D11 | 0.01153744 |
| ARV 126484 | D7 | 0.01153796 |
| Alabama | D5 | 0.01155211 |
| 22-806 | D11 | 0.01155307 |
| Reo/PA/Layer/01224B/14 | D8 | 0.01155691 |
| 22-806 | D5 | 0.01156327 |
| GuangxiR2 | GX/2010/1 | 0.01157747 |
| 22-460 | D7 | 0.01231595 |
| Reo/PA/Layer/01224B/14 | D11 | 0.01232652 |
| 22-806 | D8 | 0.01232917 |
| 22-460 | K1600600 | 0.01234511 |
| T1502036 | ARV 94594 | 0.01237458 |
| 22-460 | ARV 126484 | 0.01309110 |
| D6 | D10 | 0.01309853 |
| D12 | D10 | 0.01309853 |
| D9 | D10 | 0.01309853 |
| T1502036 | ARV 106761 | 0.01389105 |
| K1502030 | ARV 94594 | 0.01393482 |
| ARV 126695 | ARV 94594 | 0.01463205 |
| AHZJ19 | V-ARV-SD26 | 0.01465961 |
| 878-Bi-05 | 17227-M-10 | 0.01541654 |
| 22-806 | ARV 126695 | 0.01542537 |
| T1502036 | D5 | 0.01542967 |
| AHZJ19 | LY383 | 0.01543813 |
| K1502030 | ARV 106761 | 0.01544962 |
| Reo/PA/Broiler/15511/13 | 878-Bi-05 | 0.01617956 |
| 878-Bi-05 | 3457-M-11 | 0.01618352 |
| T1502036 | D8 | 0.01619461 |
| Alabama | ARV 94594 | 0.01619637 |
| Reo/PA/Layer/01224B/14 | ARV 126695 | 0.01620074 |
| 22-806 | ARV 94594 | 0.01621194 |
| T1502036 | D11 | 0.01696465 |
| K1502030 | D5 | 0.01698934 |
| T1502036 | Reo/PA/Layer/01224B/14 | 0.01699928 |
| Reo/PA/Broiler/15511/13 | 17227-M-10 | 0.01773297 |
| K1502030 | D8 | 0.01775442 |
| D1 | 878-Bi-05 | 0.01776316 |
| Alabama | Reo/PA/Layer/01224B/14 | 0.01777011 |
| 22-806 | Reo/PA/Layer/01224B/14 | 0.01778718 |
| Reo/PA/Broiler/15511/13 | 3457-M-11 | 0.01849955 |
| D7 | 878-Bi-05 | 0.01851870 |
| K1502030 | D11 | 0.01852511 |
| D1 | 17227-M-10 | 0.01854295 |
| 22-806 | Alabama | 0.01854571 |
| T1781 | 3211-V-02 | 0.01855212 |
| K1502030 | Reo/PA/Layer/01224B/14 | 0.01856288 |
| T1502036 | ARV 126695 | 0.01929675 |
| Reo/PA/Layer/27614/13 | 878-Bi-05 | 0.01930035 |
| D1 | 3457-M-11 | 0.01931093 |
| Reo/PA/Layer/01224A/14 | 17227-M-10 | 0.01931437 |
| T1502036 | 22-806 | 0.01933023 |
| D1 | Reo/PA/Broiler/15511/13 | 0.01933447 |
| Reo/PA/Layer/01224A/14 | 3457-M-11 | 0.01936308 |
| 22-460 | 878-Bi-05 | 0.02007458 |
| D7 | 17227-M-10 | 0.02007607 |
| K1600600 | 878-Bi-05 | 0.02011776 |
| AVS-B | ARV 94594 | 0.02012062 |
| D7 | 3457-M-11 | 0.02084321 |
| ARV 126484 | 878-Bi-05 | 0.02085070 |
| K1502030 | ARV 126695 | 0.02086103 |
| Reo/PA/Layer/01224A/14 | 878-Bi-05 | 0.02086571 |
| T1502036 | Alabama | 0.02087169 |
| AVS-B | ARV 106761 | 0.02088543 |
| D1 | D7 | 0.02088567 |
| K1502030 | 22-806 | 0.02089721 |
| **ARV 127720** | **D1** | **0.02094412** |
| 22-460 | 17227-M-10 | 0.02164106 |
| **22-835** | ARV 106761 | **0.02165191** |
| PHC-2020-0545 | 3457-M-11 | 0.02167748 |
| K1600600 | 17227-M-10 | 0.02168059 |
| AVS-B | D5 | 0.02168356 |
| ARV 127720 | 878-Bi-05 | 0.02170947 |
| ARV 127720 | 17227-M-10 | 0.02171042 |
| 22-835 | Reo/PA/Layer/01224B/14 | 0.02239960 |
| 22-460 | 3457-M-11 | 0.02240854 |
| ARV 126484 | 17227-M-10 | 0.02241099 |
| PHC-2020-0545 | 878-Bi-05 | 0.02241435 |
| PHC-2020-0545 | 17227-M-10 | 0.02241689 |
| K1502030 | Alabama | 0.02243988 |
| AVS-B | D8 | 0.02244890 |
| K1600600 | 3457-M-11 | 0.02244945 |
| 22-835 | D5 | 0.02245060 |
| 22-835 | ARV 94594 | 0.02245399 |
| ARV 127720 | 3457-M-11 | 0.02248035 |
| D3 | 878-Bi-05 | 0.02254309 |
| Reo/PA/Broiler/15511/13 | Reo/PA/Layer/27614/13 | 0.02317214 |
| ARV 126484 | 3457-M-11 | 0.02317817 |
| PHC-2020-0545 | Reo/PA/Broiler/15511/13 | 0.02318296 |
| Reo/PA/Broiler/15511/13 | Reo/PA/Layer/01224A/14 | 0.02318935 |
| 22-861 | D4 | 0.02320171 |
| Reo/PA/Layer/27614/13 | 17227-M-10 | 0.02320961 |
| 22-835 | D8 | 0.02321532 |
| AVS-B | D11 | 0.02322147 |
| D1 | 22-460 | 0.02325614 |
| AVS-B | Reo/PA/Layer/01224B/14 | 0.02326863 |
| D1 | K1600600 | 0.02330095 |
| D3 | 17227-M-10 | 0.02332981 |
| Reo/PA/Layer/27614/13 | 3457-M-11 | 0.02397741 |
| 23-087 | D10 | 0.02398088 |
| 22-835 | D11 | 0.02398752 |
| D1 | ARV 126484 | 0.02403039 |
| AVS-B | T1502036 | 0.02404871 |
| D3 | 3457-M-11 | 0.02410149 |
| D3 | Reo/PA/Broiler/15511/13 | 0.02413087 |
| ARV 127720 | D3 | 0.02418093 |
| D7 | Reo/PA/Layer/27614/13 | 0.02474364 |
| 23-087 | D4 | 0.02476293 |
| 22-861 | D6 | 0.02477333 |
| 22-861 | D12 | 0.02477333 |
| 22-861 | D9 | 0.02477333 |
| D1 | Reo/PA/Layer/01224A/14 | 0.02479499 |
| AHZJ19 | 3211-V-02 | 0.02482433 |
| AVS-B | 22-806 | 0.02482848 |
| PHC-2020-0545 | D7 | 0.02553968 |
| 22-861 | D10 | 0.02554295 |
| D7 | Reo/PA/Layer/01224A/14 | 0.02554674 |
| AVS-B | ARV 126695 | 0.02555844 |
| PHC-2020-0545 | D1 | 0.02556346 |
| K1600600 | Reo/PA/Layer/27614/13 | 0.02556397 |
| V-ARV-SD26 | 3211-V-02 | 0.02559085 |
| D1 | Reo/PA/Layer/27614/13 | 0.02559389 |
| ARV 127720 | Reo/PA/Broiler/15511/13 | 0.02560246 |
| AVS-B | K1502030 | 0.02562553 |
| D3 | D7 | 0.02569313 |
| PHC-2020-0545 | 22-460 | 0.02632884 |
| 22-835 | ARV 126695 | 0.02633440 |
| 23-087 | D6 | 0.02633704 |
| 23-087 | D12 | 0.02633704 |
| 23-087 | D9 | 0.02633704 |
| LY383 | 3211-V-02 | 0.02637824 |
| **23-272** | **K1600657** | **0.02638676** |
| ARV 127720 | Reo/PA/Layer/01224A/14 | 0.02639234 |

The strains displaying the closest evolutionary distance to our isolates, are shown in bold red. The evolutionary distance is shown up to d = 0.02639234.

**Table S6: Evolutionary divergence estimates between μB sequences of ARV isolates.**

| **Species 1** | **Species 2** | **Distance (*d*)** |
| --- | --- | --- |
| Reo/PA/Layer/01224B/14 | Reo/PA/Layer/01224A/14 | 0.00000100 |
| D6 | D9 | 0.00000100 |
| D6 | D12 | 0.00000100 |
| D9 | D12 | 0.00000100 |
| D8 | D11 | 0.00000100 |
| LY383 | V-ARV-SD26 | 0.00140855 |
| D8 | T1502036 | 0.00141118 |
| D11 | T1502036 | 0.00141118 |
| K1600657 | T1502036 | 0.00141118 |
| GX110058 | MS01 | 0.00281498 |
| 1733 | MS01 | 0.00281548 |
| 878-Bi-05 | 875-Bi-05 | 0.00281737 |
| C78 | GX110116 | 0.00281757 |
| GuangxiR2 | MS01 | 0.00281776 |
| D1 | D3 | 0.00281829 |
| D8 | K1600657 | 0.00282122 |
| D11 | K1600657 | 0.00282122 |
| T1502036 | Reo/PA/Broiler/05682/12 | 0.00282331 |
| 3457-M-11 | 924-Bi-05 | 0.00288163 |
| S1133 | MS01 | 0.00422587 |
| Reo/PA/Broiler/15511/13 | Reo/PA/Broiler/05682/12 | 0.00423241 |
| D8 | Reo/PA/Broiler/05682/12 | 0.00423323 |
| D11 | Reo/PA/Broiler/05682/12 | 0.00423323 |
| K1600657 | Reo/PA/Broiler/05682/12 | 0.00423325 |
| T1502036 | Reo/PA/Broiler/15511/13 | 0.00423901 |
| **22-861** | **23-087** | **0.00425794** |
| GX110116 | MS01 | 0.00563723 |
| GX/2010/1 | GX110116 | 0.00563827 |
| 1733 | GX110058 | 0.00563900 |
| T-98 | MS01 | 0.00563951 |
| GuangxiR2 | GX110058 | 0.00564362 |
| D6 | Reo/PA/Broiler/15511/13 | 0.00564435 |
| D9 | Reo/PA/Broiler/15511/13 | 0.00564435 |
| D12 | Reo/PA/Broiler/15511/13 | 0.00564435 |
| **ARV 141045** | **GX110116** | **0.00564448** |
| GuangxiR2 | 1733 | 0.00564459 |
| D2 | Reo/PA/Broiler/05682/12 | 0.00564691 |
| D8 | Reo/PA/Broiler/15511/13 | 0.00564970 |
| D11 | Reo/PA/Broiler/15511/13 | 0.00564970 |
| K1600657 | Reo/PA/Broiler/15511/13 | 0.00564973 |
| D2 | T1502036 | 0.00565573 |
| 16821-M-06 | 3457-M-11 | 0.00577770 |
| C-98 | T-98 | 0.00705132 |
| S1133 | GX110058 | 0.00705322 |
| S1133 | 1733 | 0.00705442 |
| C-98 | MS01 | 0.00705743 |
| D6 | Reo/PA/Broiler/05682/12 | 0.00705913 |
| D9 | Reo/PA/Broiler/05682/12 | 0.00705913 |
| D12 | Reo/PA/Broiler/05682/12 | 0.00705913 |
| S1133 | GuangxiR2 | 0.00706021 |
| D2 | Reo/PA/Broiler/15511/13 | 0.00706540 |
| 16821-M-06 | 924-Bi-05 | 0.00706571 |
| D2 | D8 | 0.00706677 |
| D2 | D11 | 0.00706677 |
| D2 | K1600657 | 0.00706681 |
| D6 | T1502036 | 0.00707016 |
| D9 | T1502036 | 0.00707016 |
| D12 | T1502036 | 0.00707016 |
| **ARV 94594** | **Reo/PA/Broiler/05682/12** | **0.00707824** |
| ARV 94594 | T1502036 | 0.00708933 |
| **Alabama** | **PHC-2020-0545** | **0.00844954** |
| GX/2010/1 | MS01 | 0.00846427 |
| C78 | MS01 | 0.00846757 |
| GX110116 | GX110058 | 0.00846801 |
| GX/2010/1 | C78 | 0.00846915 |
| GX110116 | 1733 | 0.00846945 |
| T-98 | GX110058 | 0.00847142 |
| T-98 | 1733 | 0.00847287 |
| ARV 141045 | MS01 | 0.00847358 |
| GX/2010/1 | ARV 141045 | 0.00847517 |
| GX110116 | GuangxiR2 | 0.00847641 |
| ARV 141045 | C78 | 0.00847848 |
| T-98 | GuangxiR2 | 0.00847983 |
| D6 | D8 | 0.00848071 |
| D9 | D8 | 0.00848071 |
| D12 | D8 | 0.00848071 |
| D6 | D11 | 0.00848071 |
| D9 | D11 | 0.00848071 |
| D12 | D11 | 0.00848071 |
| D6 | K1600657 | 0.00848076 |
| D9 | K1600657 | 0.00848076 |
| D12 | K1600657 | 0.00848076 |
| ARV 94594 | Reo/PA/Broiler/15511/13 | 0.00850205 |
| ARV 94594 | D8 | 0.00850370 |
| ARV 94594 | D11 | 0.00850370 |
| ARV 94594 | K1600657 | 0.00850375 |
| PHC-2020-0545 | SDYT2020 | 0.00986258 |
| Alabama | SDYT2020 | 0.00986428 |
| GX110116 | S1133 | 0.00988731 |
| T-98 | S1133 | 0.00989129 |
| C-98 | GX110058 | 0.00989462 |
| C-98 | 1733 | 0.00989630 |
| D6 | D2 | 0.00989877 |
| D9 | D2 | 0.00989877 |
| D12 | D2 | 0.00989877 |
| C-98 | GuangxiR2 | 0.00990445 |
| **22-460** | **Reo/PA/Broiler/05682/12** | **0.00990647** |
| 22-460 | D2 | 0.00991357 |
| 22-460 | T1502036 | 0.00992199 |
| ARV 94594 | D2 | 0.00992561 |
| GX/2010/1 | GX110058 | 0.01130196 |
| GX/2010/1 | 1733 | 0.01130388 |
| C78 | GX110058 | 0.01130637 |
| C78 | 1733 | 0.01130829 |
| GX110116 | T-98 | 0.01130985 |
| GX/2010/1 | GuangxiR2 | 0.01131318 |
| ARV 141045 | GX110058 | 0.01131439 |
| ARV 141045 | 1733 | 0.01131632 |
| C-98 | S1133 | 0.01131725 |
| C78 | GuangxiR2 | 0.01131760 |
| ARV 141045 | GuangxiR2 | 0.01132564 |
| **V-ARV-SD26** | **22-835** | **0.01133020** |
| 22-460 | Reo/PA/Broiler/15511/13 | 0.01133260 |
| 22-460 | D8 | 0.01133477 |
| 22-460 | D11 | 0.01133477 |
| 22-460 | K1600657 | 0.01133484 |
| **ARV 126484** | 22-835 | **0.01133741** |
| ARV 94594 | D6 | 0.01134433 |
| ARV 94594 | D9 | 0.01134433 |
| ARV 94594 | D12 | 0.01134433 |
| 22-861 | **ARV 127720** | **0.01139474** |
| ARV 127720 | **22-806** | **0.01139972** |
| GX/2010/1 | S1133 | 0.01272498 |
| C78 | S1133 | 0.01272995 |
| GX110116 | C-98 | 0.01273812 |
| ARV 141045 | S1133 | 0.01273898 |
| 875-Bi-05 | D3 | 0.01274532 |
| Reo/PA/Layer/01224B/14 | Reo/PA/Broiler/05682/12 | 0.01274663 |
| Reo/PA/Layer/01224A/14 | Reo/PA/Broiler/05682/12 | 0.01274663 |
| 875-Bi-05 | D1 | 0.01274972 |
| LY383 | 22-835 | 0.01275534 |
| Reo/PA/Layer/01224B/14 | D2 | 0.01275580 |
| Reo/PA/Layer/01224A/14 | D2 | 0.01275580 |
| Reo/PA/Layer/01224B/14 | T1502036 | 0.01276663 |
| Reo/PA/Layer/01224A/14 | T1502036 | 0.01276663 |
| 23-087 | ARV 127720 | 0.01281805 |
| T1781 | 284-V-06 | 0.01296184 |
| GX/2010/1 | T-98 | 0.01415143 |
| C78 | T-98 | 0.01415696 |
| ARV 141045 | T-98 | 0.01416700 |
| 22-460 | D6 | 0.01417609 |
| 22-460 | D9 | 0.01417609 |
| 22-460 | D12 | 0.01417609 |
| Reo/PA/Layer/01224B/14 | Reo/PA/Broiler/15511/13 | 0.01417661 |
| Reo/PA/Layer/01224A/14 | Reo/PA/Broiler/15511/13 | 0.01417661 |
| Reo/PA/Layer/01224B/14 | D8 | 0.01417931 |
| Reo/PA/Layer/01224A/14 | D8 | 0.01417931 |
| Reo/PA/Layer/01224B/14 | D11 | 0.01417931 |
| Reo/PA/Layer/01224A/14 | D11 | 0.01417931 |
| Reo/PA/Layer/01224B/14 | K1600657 | 0.01417940 |
| Reo/PA/Layer/01224A/14 | K1600657 | 0.01417940 |
| 22-460 | ARV 94594 | 0.01421461 |
| 22-861 | 22-806 | 0.01425726 |
| 4599-V-04 | 17227-M-10 | 0.01555547 |
| D7 | AVS-B | 0.01557713 |
| GX/2010/1 | C-98 | 0.01558437 |
| C78 | C-98 | 0.01559047 |
| 878-Bi-05 | D3 | 0.01559793 |
| ARV 141045 | C-98 | 0.01560153 |
| 878-Bi-05 | D1 | 0.01560331 |
| V-ARV-SD26 | ARV 126484 | 0.01563767 |
| 23-087 | 22-806 | 0.01568174 |
| 22-835 | 875-Bi-05 | 0.01700626 |
| 22-835 | 878-Bi-05 | 0.01700667 |
| V-ARV-SD26 | 875-Bi-05 | 0.01702317 |
| V-ARV-SD26 | 878-Bi-05 | 0.01702358 |
| Reo/PA/Layer/01224B/14 | D6 | 0.01702433 |
| Reo/PA/Layer/01224A/14 | D6 | 0.01702433 |
| Reo/PA/Layer/01224B/14 | D9 | 0.01702433 |
| Reo/PA/Layer/01224A/14 | D9 | 0.01702433 |
| Reo/PA/Layer/01224B/14 | D12 | 0.01702433 |
| Reo/PA/Layer/01224A/14 | D12 | 0.01702433 |
| Reo/PA/Layer/01224B/14 | 22-460 | 0.01704995 |
| Reo/PA/Layer/01224A/14 | 22-460 | 0.01704995 |
| Reo/PA/Layer/01224B/14 | ARV 94594 | 0.01707062 |
| Reo/PA/Layer/01224A/14 | ARV 94594 | 0.01707062 |
| LY383 | ARV 126484 | 0.01707114 |
| V-ARV-SD26 | D3 | 0.01708076 |
| V-ARV-SD26 | D1 | 0.01708667 |
| LY383 | 875-Bi-05 | 0.01845464 |
| LY383 | 878-Bi-05 | 0.01845508 |
| **23-272** | **V-ARV-SD26** | **0.01846578** |
| Reo/PA/Broiler/05682/12 | V-ARV-SD26 | 0.01847314 |
| T1502036 | V-ARV-SD26 | 0.01850226 |
| **ARV 122301** | **V-ARV-SD26** | **0.01851182** |
| LY383 | D3 | 0.01851709 |
| LY383 | D1 | 0.01852349 |
| 526 | LY383 | 0.01864412 |
| 17203-M-06 | T1781 | 0.01873372 |
| Reo/PA/Broiler/15511/13 | 875-Bi-05 | 0.01988721 |
| Reo/PA/Broiler/15511/13 | 878-Bi-05 | 0.01988769 |
| 23-272 | LY383 | 0.01990006 |
| Reo/PA/Broiler/05682/12 | LY383 | 0.01990804 |
| Reo/PA/Broiler/15511/13 | V-ARV-SD26 | 0.01991352 |
| D8 | V-ARV-SD26 | 0.01991728 |
| D11 | V-ARV-SD26 | 0.01991728 |
| K1600657 | V-ARV-SD26 | 0.01991742 |
| ARV 122301 | D3 | 0.01992520 |
| ARV 122301 | D1 | 0.01993207 |
| Reo/PA/Layer/27614/13 | V-ARV-SD26 | 0.01993235 |
| T1502036 | LY383 | 0.01993946 |
| ARV 122301 | LY383 | 0.01994976 |
| D2 | ARV 122301 | 0.02000254 |
| 526 | V-ARV-SD26 | 0.02008942 |
| Reo/PA/Broiler/05682/12 | 875-Bi-05 | 0.02131892 |
| Reo/PA/Broiler/05682/12 | 878-Bi-05 | 0.02131944 |
| ARV 122301 | 875-Bi-05 | 0.02132245 |
| D2 | 875-Bi-05 | 0.02132644 |
| ARV 126484 | 875-Bi-05 | 0.02134863 |
| ARV 126484 | 878-Bi-05 | 0.02134915 |
| 22-835 | D3 | 0.02134918 |
| D2 | V-ARV-SD26 | 0.02135008 |
| Reo/PA/Broiler/15511/13 | LY383 | 0.02135085 |
| T1502036 | 875-Bi-05 | 0.02135256 |
| T1502036 | 878-Bi-05 | 0.02135309 |
| D8 | LY383 | 0.02135488 |
| D11 | LY383 | 0.02135488 |
| K1600657 | LY383 | 0.02135502 |
| 22-835 | D1 | 0.02135653 |
| Reo/PA/Layer/27614/13 | LY383 | 0.02137104 |
| Reo/PA/Broiler/05682/12 | D3 | 0.02139109 |
| Reo/PA/Broiler/05682/12 | D1 | 0.02139848 |
| D2 | D3 | 0.02139866 |
| D2 | D1 | 0.02140605 |
| T1502036 | D3 | 0.02142496 |
| T1502036 | D1 | 0.02143237 |
| D4 | AVS-B | 0.02270335 |
| 23-272 | 22-835 | 0.02272672 |
| D6 | 875-Bi-05 | 0.02274477 |
| D9 | 875-Bi-05 | 0.02274477 |
| D12 | 875-Bi-05 | 0.02274477 |
| D6 | 878-Bi-05 | 0.02274533 |
| D9 | 878-Bi-05 | 0.02274533 |
| D12 | 878-Bi-05 | 0.02274533 |
| D8 | 875-Bi-05 | 0.02276664 |
| D11 | 875-Bi-05 | 0.02276664 |
| K1600657 | 875-Bi-05 | 0.02276679 |
| D8 | 878-Bi-05 | 0.02276720 |
| D11 | 878-Bi-05 | 0.02276720 |
| K1600657 | 878-Bi-05 | 0.02276735 |
| Reo/PA/Broiler/05682/12 | Reo/PA/Layer/27614/13 | 0.02276971 |
| D6 | V-ARV-SD26 | 0.02277494 |
| D9 | V-ARV-SD26 | 0.02277494 |
| D12 | V-ARV-SD26 | 0.02277494 |
| Reo/PA/Broiler/15511/13 | 22-835 | 0.02278430 |
| **ARV 106761** | **D3** | **0.02278719** |
| D2 | LY383 | 0.02278938 |
| ARV 106761 | ARV 122301 | 0.02279070 |
| ARV 106761 | D1 | 0.02279503 |
| T1502036 | Reo/PA/Layer/27614/13 | 0.02280570 |
| ARV 126484 | D3 | 0.02283392 |
| ARV 94594 | V-ARV-SD26 | 0.02283706 |
| Reo/PA/Broiler/15511/13 | D3 | 0.02283946 |
| ARV 126484 | D1 | 0.02284179 |
| D8 | D3 | 0.02284377 |
| D11 | D3 | 0.02284377 |
| K1600657 | D3 | 0.02284392 |
| ARV 106761 | V-ARV-SD26 | 0.02284403 |
| Reo/PA/Broiler/15511/13 | D1 | 0.02284734 |
| D8 | D1 | 0.02285165 |
| D11 | D1 | 0.02285165 |
| K1600657 | D1 | 0.02285181 |
| Reo/PA/Broiler/05682/12 | ARV 122301 | 0.02287272 |
| T1502036 | ARV 122301 | 0.02290903 |
| 23-272 | 875-Bi-05 | 0.02414014 |
| 23-272 | 878-Bi-05 | 0.02414074 |
| Reo/PA/Broiler/15511/13 | 23-272 | 0.02417670 |
| D4 | D7 | 0.02418368 |
| Reo/PA/Layer/27614/13 | 875-Bi-05 | 0.02419067 |
| Reo/PA/Layer/27614/13 | 878-Bi-05 | 0.02419127 |
| ARV 122301 | 878-Bi-05 | 0.02419718 |
| D2 | 878-Bi-05 | 0.02420167 |
| D6 | LY383 | 0.02421532 |
| D9 | LY383 | 0.02421532 |
| D12 | LY383 | 0.02421532 |
| Reo/PA/Broiler/15511/13 | Reo/PA/Layer/27614/13 | 0.02421643 |
| Reo/PA/Broiler/05682/12 | 23-272 | 0.02421717 |
| D8 | Reo/PA/Layer/27614/13 | 0.02422097 |
| D11 | Reo/PA/Layer/27614/13 | 0.02422097 |
| Reo/PA/Broiler/05682/12 | 22-835 | 0.02422107 |
| K1600657 | Reo/PA/Layer/27614/13 | 0.02422114 |
| ARV 122301 | 22-835 | 0.02422324 |
| Reo/PA/Layer/27614/13 | 22-835 | 0.02422352 |
| 17203-M-06 | 284-V-06 | 0.02423906 |
| ARV 106761 | 22-835 | 0.02424715 |
| T1502036 | 23-272 | 0.02425550 |
| T1502036 | 22-835 | 0.02425941 |
| ARV 94594 | LY383 | 0.02428142 |
| ARV 106761 | LY383 | 0.02428871 |
| Reo/PA/Broiler/15511/13 | ARV 122301 | 0.02432605 |
| D8 | ARV 122301 | 0.02433063 |
| D11 | ARV 122301 | 0.02433063 |
| K1600657 | ARV 122301 | 0.02433080 |
| 526 | 22-835 | 0.02438976 |
| D6 | 22-835 | 0.02565102 |
| D9 | 22-835 | 0.02565102 |
| D12 | 22-835 | 0.02565102 |
| Reo/PA/Layer/01224B/14 | V-ARV-SD26 | 0.02565211 |
| Reo/PA/Layer/01224A/14 | V-ARV-SD26 | 0.02565211 |
| D2 | Reo/PA/Layer/27614/13 | 0.02565794 |
| D8 | 23-272 | 0.02567156 |
| D11 | 23-272 | 0.02567156 |
| K1600657 | 23-272 | 0.02567174 |
| D8 | 22-835 | 0.02567576 |
| D11 | 22-835 | 0.02567576 |
| K1600657 | 22-835 | 0.02567595 |
| 22-460 | V-ARV-SD26 | 0.02568411 |
| Reo/PA/Layer/27614/13 | D3 | 0.02568558 |
| Reo/PA/Layer/27614/13 | D1 | 0.02569442 |
| ARV 94594 | 875-Bi-05 | 0.02569623 |
| ARV 94594 | 878-Bi-05 | 0.02569688 |
| D6 | D3 | 0.02571312 |
| D9 | D3 | 0.02571312 |
| D12 | D3 | 0.02571312 |
| D5 | D4 | 0.02571322 |
| 23-272 | D3 | 0.02571464 |
| ARV 122301 | ARV 126484 | 0.02571726 |
| D6 | D1 | 0.02572198 |
| D9 | D1 | 0.02572198 |
| D12 | D1 | 0.02572198 |
| 23-272 | D1 | 0.02572349 |
| D2 | 23-272 | 0.02573804 |
| Reo/PA/Broiler/15511/13 | ARV 126484 | 0.02575702 |
| Reo/PA/Broiler/05682/12 | 526 | 0.02576738 |
| ARV 122301 | 526 | 0.02577058 |
| 23-272 | ARV 122301 | 0.02577605 |
| ARV 94594 | D3 | 0.02578350 |
| ARV 94594 | D1 | 0.02579241 |
| T1502036 | 526 | 0.02580838 |
| 526 | D3 | 0.02586958 |
| 526 | D1 | 0.02587854 |
| 526 | ARV 126484 | 0.02589356 |
| **ARV 126695** | **D5** | **0.02699561** |
| D6 | 23-272 | 0.02704069 |
| D9 | 23-272 | 0.02704069 |
| D12 | 23-272 | 0.02704069 |
| 4599-V-04 | V-ARV-SD26 | 0.02704931 |
| D6 | Reo/PA/Layer/27614/13 | 0.02708510 |
| D9 | Reo/PA/Layer/27614/13 | 0.02708510 |
| D12 | Reo/PA/Layer/27614/13 | 0.02708510 |
| 23-272 | ARV 126484 | 0.02708940 |
| ARV 106761 | 875-Bi-05 | 0.02709495 |
| ARV 106761 | 878-Bi-05 | 0.02709563 |
| Reo/PA/Layer/01224B/14 | LY383 | 0.02709623 |
| Reo/PA/Layer/01224A/14 | LY383 | 0.02709623 |
| Reo/PA/Layer/01224B/14 | Reo/PA/Layer/27614/13 | 0.02709625 |
| Reo/PA/Layer/01224A/14 | Reo/PA/Layer/27614/13 | 0.02709625 |
| D2 | 22-835 | 0.02711518 |
| Reo/PA/Layer/27614/13 | 23-272 | 0.02712709 |
| 22-460 | LY383 | 0.02713007 |
| Reo/PA/Layer/27614/13 | ARV 122301 | 0.02714232 |
| ARV 94594 | Reo/PA/Layer/27614/13 | 0.02715908 |
| Reo/PA/Broiler/05682/12 | ARV 126484 | 0.02720219 |
| D6 | ARV 122301 | 0.02720771 |
| D9 | ARV 122301 | 0.02720771 |
| D12 | ARV 122301 | 0.02720771 |
| Reo/PA/Layer/01224B/14 | ARV 122301 | 0.02721509 |
| Reo/PA/Layer/01224A/14 | ARV 122301 | 0.02721509 |
| Reo/PA/Broiler/15511/13 | 526 | 0.02722568 |
| D8 | 526 | 0.02723078 |
| D11 | 526 | 0.02723078 |
| K1600657 | 526 | 0.02723098 |
| T1502036 | ARV 126484 | 0.02724550 |
| 22-460 | ARV 122301 | 0.02724922 |
| 526 | 875-Bi-05 | 0.02727231 |
| 526 | 878-Bi-05 | 0.02727301 |
| ARV 94594 | ARV 122301 | 0.02728235 |
| 4599-V-04 | LY383 | 0.02849291 |
| Reo/PA/Layer/01224B/14 | 875-Bi-05 | 0.02850740 |
| Reo/PA/Layer/01224A/14 | 875-Bi-05 | 0.02850740 |
| Reo/PA/Layer/01224B/14 | 878-Bi-05 | 0.02850812 |
| Reo/PA/Layer/01224A/14 | 878-Bi-05 | 0.02850812 |
| **ARV 115940** | **D5** | **0.02853654** |
| 22-460 | 875-Bi-05 | 0.02854298 |

The strains displaying the closest evolutionary distance to our isolates, are shown in bold red. The evolutionary distance is shown up to d = 0.02854298.

**Table S7: Evolutionary divergence estimates between σB sequences of ARV isolates.**

| **Species 1** | **Species 2** | **Distance (*d*)** |
| --- | --- | --- |
| LY383 | V-ARV-SD26 | 0.00000100 |
| D1 | D3 | 0.00000100 |
| **22-460** | **22-835** | **0.00000100** |
| **117816** | **ARV 115940** | **0.00000100** |
| T1502036 | K1502030 | 0.00000100 |
| D12 | D9 | 0.00000100 |
| D12 | D6 | 0.00000100 |
| D9 | D6 | 0.00000100 |
| 1733 | T98 | 0.00000100 |
| K1600600 | 117816 | 0.00267215 |
| K1600600 | ARV 115940 | 0.00267215 |
| C98 | 1733 | 0.00268270 |
| C98 | T98 | 0.00268270 |
| S1133 | 1733 | 0.00268329 |
| S1133 | T98 | 0.00268329 |
| GX110058 | 1733 | 0.00268332 |
| GX110058 | T98 | 0.00268332 |
| GuangxiR2 | 1733 | 0.00268501 |
| GuangxiR2 | T98 | 0.00268501 |
| LY383 | K1600657 | 0.00268609 |
| V-ARV-SD26 | K1600657 | 0.00268609 |
| 924-Bi-05 | 16821-M-06 | 0.00269173 |
| **ARV 127720** | **D1** | **0.00535169** |
| **ARV 127720** | **D3** | **0.00535169** |
| **ARV 126695** | **AVS-B** | **0.00535940** |
| **GX/2010/1** | **ARV 141045** | **0.00536213** |
| S1133 | C98 | 0.00537083 |
| GX110058 | C98 | 0.00537084 |
| S1133 | GX110058 | 0.00537205 |
| GuangxiR2 | C98 | 0.00537428 |
| S1133 | GuangxiR2 | 0.00537549 |
| GuangxiR2 | GX110058 | 0.00537551 |
| 3457-M-11 | 16821-M-06 | 0.00538094 |
| MS01 | ARV 141045 | 0.00538519 |
| SDYT2020 | LY383 | 0.00538551 |
| SDYT2020 | V-ARV-SD26 | 0.00538551 |
| **22-861** | ARV 127720 | **0.00804573** |
| D7 | AVS-B | 0.00806156 |
| 17227-M-10 | 3457-M-11 | 0.00806527 |
| 17227-M-10 | 16821-M-06 | 0.00806710 |
| 1733 | MS01 | 0.00807595 |
| T98 | MS01 | 0.00807595 |
| SDYT2020 | K1600657 | 0.00808360 |
| PHC-2020-0545 | LY383 | 0.00808379 |
| PHC-2020-0545 | V-ARV-SD26 | 0.00808379 |
| AHZJ19 | LY383 | 0.00808677 |
| AHZJ19 | V-ARV-SD26 | 0.00808677 |
| 3457-M-11 | 924-Bi-05 | 0.00809919 |
| 17203-M-06 | 16821-M-06 | 0.00812605 |
| T1781 | 3457-M-11 | 0.00812611 |
| T1781 | 16821-M-06 | 0.00812797 |
| C98 | MS01 | 0.01077642 |
| S1133 | MS01 | 0.01077888 |
| GX110058 | MS01 | 0.01077891 |
| GX110116 | MS01 | 0.01078467 |
| PHC-2020-0545 | K1600657 | 0.01078552 |
| GuangxiR2 | MS01 | 0.01078586 |
| T1502036 | AVS-B | 0.01078595 |
| AHZJ19 | K1600657 | 0.01078951 |
| GX/2010/1 | MS01 | 0.01079116 |
| 17227-M-10 | 924-Bi-05 | 0.01079319 |
| GX110116 | ARV 141045 | 0.01080433 |
| 1733 | ARV 141045 | 0.01080643 |
| T98 | ARV 141045 | 0.01080643 |
| C78 | MS01 | 0.01081970 |
| K1502030 | AVS-B | 0.01082802 |
| T1781 | 17227-M-10 | 0.01082903 |
| C78 | ARV 141045 | 0.01083949 |
| 17203-M-06 | 924-Bi-05 | 0.01087218 |
| T1781 | 924-Bi-05 | 0.01087490 |
| D4 | 117816 | 0.01340494 |
| D4 | ARV 115940 | 0.01340494 |
| 22-861 | D1 | 0.01344107 |
| 22-861 | D3 | 0.01344107 |
| D5 | 117816 | 0.01344584 |
| D5 | ARV 115940 | 0.01344584 |
| D7 | ARV 126695 | 0.01346117 |
| **ARV 94594** | **AVS-B** | **0.01346955** |
| PHC-2020-0545 | SDYT2020 | 0.01351539 |
| C98 | ARV 141045 | 0.01351881 |
| AHZJ19 | SDYT2020 | 0.01352041 |
| S1133 | ARV 141045 | 0.01352192 |
| GX110058 | ARV 141045 | 0.01352196 |
| GuangxiR2 | ARV 141045 | 0.01353072 |
| 17203-M-06 | 3457-M-11 | 0.01358375 |
| GX110116 | 1733 | 0.01359874 |
| GX110116 | T98 | 0.01359874 |
| D4 | K1600600 | 0.01609559 |
| D5 | K1600600 | 0.01614478 |
| **ARV 126484** | **AVS-B** | **0.01615251** |
| D5 | AVS-B | 0.01616611 |
| T1502036 | D7 | 0.01617225 |
| **22-806** | **MS01** | **0.01618449** |
| 526 | D2 | 0.01618603 |
| 22-806 | 1733 | 0.01619142 |
| 22-806 | T98 | 0.01619142 |
| T1502036 | ARV 126695 | 0.01620914 |
| 22-806 | ARV 141045 | 0.01621410 |
| 22-460 | Reo/PA/Broiler/05682/12 | 0.01623131 |
| 22-835 | Reo/PA/Broiler/05682/12 | 0.01623131 |
| K1502030 | D7 | 0.01623527 |
| PHC-2020-0545 | AHZJ19 | 0.01623574 |
| GX110116 | GX/2010/1 | 0.01623787 |
| 1733 | GX/2010/1 | 0.01624109 |
| T98 | GX/2010/1 | 0.01624109 |
| K1502030 | ARV 126695 | 0.01627245 |
| C78 | GX110116 | 0.01628098 |
| C78 | 1733 | 0.01628422 |
| C78 | T98 | 0.01628422 |
| C78 | GX/2010/1 | 0.01629093 |
| 17203-M-06 | 17227-M-10 | 0.01629170 |
| GX110116 | C98 | 0.01633092 |
| GX110116 | S1133 | 0.01633472 |
| GX110116 | GX110058 | 0.01633477 |
| GX110116 | GuangxiR2 | 0.01634542 |
| T1781 | 17203-M-06 | 0.01641573 |
| 117816 | AVS-B | 0.01887660 |
| ARV 115940 | AVS-B | 0.01887660 |
| ARV 94594 | ARV 126695 | 0.01889308 |
| 22-806 | C98 | 0.01890490 |
| ARV 126484 | D7 | 0.01890666 |
| 22-806 | S1133 | 0.01890928 |
| 22-806 | GX110058 | 0.01890935 |
| 22-806 | GuangxiR2 | 0.01892159 |
| **ARV 122301** | **AVS-B** | **0.01894594** |
| C98 | GX/2010/1 | 0.01896304 |
| S1133 | GX/2010/1 | 0.01896745 |
| GX110058 | GX/2010/1 | 0.01896752 |
| GuangxiR2 | GX/2010/1 | 0.01897984 |
| C78 | C98 | 0.01901346 |
| C78 | S1133 | 0.01901788 |
| C78 | GX110058 | 0.01901796 |
| C78 | GuangxiR2 | 0.01903034 |
| 22-806 | GX110116 | 0.01904195 |
| D4 | D5 | 0.02155410 |
| ARV 126484 | ARV 126695 | 0.02157730 |
| K1600600 | AVS-B | 0.02158626 |
| Reo/PA/Layer/01224A/14 | D2 | 0.02159023 |
| D5 | ARV 126695 | 0.02159562 |
| 117816 | ARV 126484 | 0.02162923 |
| ARV 115940 | ARV 126484 | 0.02162923 |
| 22-460 | AVS-B | 0.02163262 |
| 22-835 | AVS-B | 0.02163262 |
| D5 | ARV 94594 | 0.02164255 |
| ARV 94594 | D7 | 0.02165252 |
| 22-806 | GX/2010/1 | 0.02166065 |
| ARV 126484 | T1502036 | 0.02167218 |
| D5 | T1502036 | 0.02169065 |
| 22-806 | C78 | 0.02171821 |
| ARV 126484 | K1502030 | 0.02175701 |
| D5 | K1502030 | 0.02177564 |
| 3211-V-02 | 1733 | 0.02199080 |
| 3211-V-02 | T98 | 0.02199080 |
| 117816 | ARV 126695 | 0.02431586 |
| ARV 115940 | ARV 126695 | 0.02431586 |
| Reo/PA/Layer/27614/13 | Reo/PA/Layer/01224A/14 | 0.02432405 |
| K1600600 | ARV 126484 | 0.02434743 |
| 22-460 | 117816 | 0.02435071 |
| 22-835 | 117816 | 0.02435071 |
| 22-460 | ARV 115940 | 0.02435071 |
| 22-835 | ARV 115940 | 0.02435071 |
| D5 | D7 | 0.02436298 |
| ARV 122301 | ARV 126695 | 0.02440534 |
| ARV 127720 | 22-460 | 0.02441070 |
| ARV 127720 | 22-835 | 0.02441070 |
| Reo/PA/Broiler/05682/12 | AVS-B | 0.02441420 |
| 117816 | T1502036 | 0.02442267 |
| ARV 115940 | T1502036 | 0.02442267 |
| ARV 94594 | T1502036 | 0.02444312 |
| ARV 127720 | Reo/PA/Broiler/05682/12 | 0.02445259 |
| Reo/PA/Broiler/05682/12 | 117816 | 0.02448787 |
| Reo/PA/Broiler/05682/12 | ARV 115940 | 0.02448787 |
| 117816 | K1502030 | 0.02451841 |
| ARV 115940 | K1502030 | 0.02451841 |
| ARV 94594 | K1502030 | 0.02453902 |
| 3211-V-02 | C98 | 0.02475263 |
| 3211-V-02 | S1133 | 0.02475850 |
| 3211-V-02 | GX110058 | 0.02475864 |
| 3211-V-02 | GuangxiR2 | 0.02477491 |
| D4 | AVS-B | 0.02701753 |
| K1600600 | ARV 126695 | 0.02703384 |
| D2 | 3211-V-02 | 0.02704625 |
| ARV 127720 | T1502036 | 0.02706329 |
| D5 | ARV 126484 | 0.02707020 |
| 22-460 | K1600600 | 0.02707262 |
| 22-835 | K1600600 | 0.02707262 |
| 22-460 | ARV 126484 | 0.02708518 |
| 22-835 | ARV 126484 | 0.02708518 |
| 22-460 | ARV 126695 | 0.02709203 |
| 22-835 | ARV 126695 | 0.02709203 |
| 117816 | D7 | 0.02709312 |
| ARV 115940 | D7 | 0.02709312 |
| ARV 127720 | AVS-B | 0.02712634 |
| K1600600 | T1502036 | 0.02715233 |
| Reo/PA/Layer/01224A/14 | 526 | 0.02715302 |
| ARV 127720 | K1502030 | 0.02716908 |
| ARV 122301 | D7 | 0.02719308 |
| 22-460 | T1502036 | 0.02721102 |
| 22-835 | T1502036 | 0.02721102 |
| Reo/PA/Broiler/05682/12 | K1600600 | 0.02722502 |
| K1600600 | K1502030 | 0.02725880 |
| Reo/PA/Broiler/05682/12 | ARV 126484 | 0.02728170 |
| 22-460 | K1502030 | 0.02731796 |
| 22-835 | K1502030 | 0.02731796 |
| 3211-V-02 | 22-806 | 0.02749503 |
| 3211-V-02 | MS01 | 0.02751825 |
| 3211-V-02 | ARV 141045 | 0.02756973 |
| LY383 | AVS-B | 0.02762190 |
| V-ARV-SD26 | AVS-B | 0.02762190 |
| **ARV 106761** | **117816** | **0.02979104** |
| **ARV 106761** | ARV 115940 | **0.02979104** |
| Reo/PA/Layer/27614/13 | D2 | 0.02980930 |
| K1600600 | D7 | 0.02982022 |
| ARV 126484 | ARV 94594 | 0.02982775 |
| 117816 | ARV 94594 | 0.02983974 |
| ARV 115940 | ARV 94594 | 0.02983974 |
| ARV 127720 | 117816 | 0.02985590 |
| ARV 127720 | ARV 115940 | 0.02985590 |
| 22-460 | D7 | 0.02988460 |
| 22-835 | D7 | 0.02988460 |
| Reo/PA/Broiler/05682/12 | ARV 126695 | 0.02989639 |
| D1 | 22-460 | 0.02990563 |
| D3 | 22-460 | 0.02990563 |
| D1 | 22-835 | 0.02990563 |
| D3 | 22-835 | 0.02990563 |
| 3457-M-11 | AVS-B | 0.02991117 |
| 16821-M-06 | AVS-B | 0.02991826 |
| 3457-M-11 | D5 | 0.02992606 |
| 16821-M-06 | D5 | 0.02993316 |
| D1 | Reo/PA/Broiler/05682/12 | 0.02995706 |
| D3 | Reo/PA/Broiler/05682/12 | 0.02995706 |
| Reo/PA/Broiler/05682/12 | ARV 106761 | 0.02999474 |
| ARV 122301 | T1502036 | 0.03001562 |
| Reo/PA/Broiler/05682/12 | T1502036 | 0.03002776 |
| 924-Bi-05 | AVS-B | 0.03005762 |
| ARV 122301 | K1502030 | 0.03013388 |
| Reo/PA/Broiler/05682/12 | K1502030 | 0.03014611 |
| K1600657 | AVS-B | 0.03039677 |
| LY383 | T1502036 | 0.03046540 |
| V-ARV-SD26 | T1502036 | 0.03046540 |
| 3211-V-02 | GX110116 | 0.03051876 |
| LY383 | K1502030 | 0.03058688 |
| V-ARV-SD26 | K1502030 | 0.03058688 |
| D4 | ARV 126484 | 0.03247396 |
| D4 | ARV 126695 | 0.03248227 |
| **23-272** | **D12** | **0.03250398** |
| **23-272** | **D9** | **0.03250398** |
| **23-272** | **D6** | **0.03250398** |
| ARV 106761 | K1600600 | 0.03251886 |
| 22-460 | D4 | 0.03252896 |
| 22-835 | D4 | 0.03252896 |
| ARV 127720 | D7 | 0.03254008 |
| D1 | T1502036 | 0.03255292 |
| D3 | T1502036 | 0.03255292 |
| ARV 127720 | D4 | 0.03255394 |
| K1600600 | ARV 94594 | 0.03257209 |
| 22-460 | ARV 106761 | 0.03258643 |
| 22-835 | ARV 106761 | 0.03258643 |
| ARV 127720 | K1600600 | 0.03258930 |
| D1 | 117816 | 0.03260419 |
| D3 | 117816 | 0.03260419 |
| D1 | ARV 115940 | 0.03260419 |
| D3 | ARV 115940 | 0.03260419 |
| ARV 127720 | ARV 126484 | 0.03260435 |
| ARV 127720 | ARV 126695 | 0.03261272 |
| 17227-M-10 | AVS-B | 0.03261367 |
| D4 | T1502036 | 0.03262404 |
| D1 | AVS-B | 0.03262841 |
| D3 | AVS-B | 0.03262841 |
| **23-087** | 22-806 | **0.03262936** |
| 17227-M-10 | D5 | 0.03262985 |
| ARV 106761 | ARV 126484 | 0.03262987 |
| 22-460 | D5 | 0.03263009 |
| 22-835 | D5 | 0.03263009 |
| 117816 | ARV 122301 | 0.03265835 |
| ARV 115940 | ARV 122301 | 0.03265835 |
| D1 | K1502030 | 0.03268041 |
| D3 | K1502030 | 0.03268041 |
| 23-272 | 22-806 | 0.03268394 |
| 22-861 | 22-460 | 0.03269923 |
| 22-861 | 22-835 | 0.03269923 |
| Reo/PA/Broiler/05682/12 | D7 | 0.03270570 |
| ARV 122301 | ARV 94594 | 0.03271611 |
| 3457-M-11 | T1502036 | 0.03272479 |
| 16821-M-06 | T1502036 | 0.03273261 |
| D4 | K1502030 | 0.03275206 |
| 22-861 | Reo/PA/Broiler/05682/12 | 0.03275558 |
| 924-Bi-05 | D5 | 0.03276863 |
| 3457-M-11 | K1502030 | 0.03285361 |
| 16821-M-06 | K1502030 | 0.03286149 |
| T1781 | AVS-B | 0.03286171 |
| T1781 | D5 | 0.03287814 |
| 924-Bi-05 | T1502036 | 0.03288519 |
| 924-Bi-05 | K1502030 | 0.03301526 |
| 3211-V-02 | GX/2010/1 | 0.03313228 |
| SDYT2020 | T1502036 | 0.03316310 |
| SDYT2020 | D7 | 0.03318434 |
| LY383 | ARV 126695 | 0.03319293 |
| V-ARV-SD26 | ARV 126695 | 0.03319293 |
| AHZJ19 | AVS-B | 0.03321275 |
| 3211-V-02 | C78 | 0.03322191 |
| 16821-M-06 | K1600657 | 0.03323449 |
| SDYT2020 | AVS-B | 0.03323523 |
| LY383 | D7 | 0.03323765 |
| V-ARV-SD26 | D7 | 0.03323765 |
| K1600657 | T1502036 | 0.03324882 |
| SDYT2020 | K1502030 | 0.03329501 |
| K1600657 | K1502030 | 0.03338140 |
| 924-Bi-05 | K1600657 | 0.03339128 |
| D2 | 22-806 | 0.03525053 |
| D4 | D7 | 0.03528715 |
| D1 | D4 | 0.03530362 |
| D3 | D4 | 0.03530362 |
| ARV 106761 | AVS-B | 0.03530691 |
| D2 | 1733 | 0.03530708 |
| D2 | T98 | 0.03530708 |
| D1 | K1600600 | 0.03534201 |
| D3 | K1600600 | 0.03534201 |
| 22-861 | T1502036 | 0.03534678 |
| Reo/PA/Broiler/05682/12 | D4 | 0.03535239 |
| ARV 127720 | ARV 106761 | 0.03536057 |
| D1 | ARV 106761 | 0.03536057 |
| D3 | ARV 106761 | 0.03536057 |
| K1600600 | ARV 122301 | 0.03540125 |
| 22-460 | ARV 94594 | 0.03540963 |
| 22-835 | ARV 94594 | 0.03540963 |
| 3457-M-11 | ARV 126695 | 0.03541666 |
| ARV 122301 | ARV 126484 | 0.03541759 |
| 16821-M-06 | ARV 126695 | 0.03542515 |
| 22-861 | AVS-B | 0.03542865 |
| 17227-M-10 | T1502036 | 0.03543344 |
| D5 | ARV 122301 | 0.03544842 |
| Reo/PA/Broiler/05682/12 | D5 | 0.03546278 |
| ARV 127720 | ARV 122301 | 0.03547648 |
| 22-460 | ARV 122301 | 0.03547806 |
| 22-835 | ARV 122301 | 0.03547806 |
| 22-861 | K1502030 | 0.03548549 |
| 23-087 | 1733 | 0.03549224 |
| 23-087 | T98 | 0.03549224 |
| LY383 | 117816 | 0.03549711 |
| V-ARV-SD26 | 117816 | 0.03549711 |
| LY383 | ARV 115940 | 0.03549711 |
| V-ARV-SD26 | ARV 115940 | 0.03549711 |
| 16821-M-06 | ARV 127720 | 0.03550713 |
| D2 | 23-087 | 0.03553228 |
| 17227-M-10 | K1502030 | 0.03557282 |
| 23-087 | 3211-V-02 | 0.03557510 |
| K1600657 | Reo/PA/Broiler/05682/12 | 0.03558696 |
| 924-Bi-05 | ARV 126695 | 0.03558981 |
| LY383 | ARV 126484 | 0.03566719 |
| V-ARV-SD26 | ARV 126484 | 0.03566719 |
| 924-Bi-05 | ARV 127720 | 0.03567252 |
| T1781 | T1502036 | 0.03570359 |
| 17203-M-06 | AVS-B | 0.03575443 |
| 17203-M-06 | D5 | 0.03577233 |
| T1781 | K1502030 | 0.03584510 |
| K1600657 | ARV 126695 | 0.03597452 |
| PHC-2020-0545 | AVS-B | 0.03602236 |
| K1600657 | D7 | 0.03602287 |
| 16821-M-06 | LY383 | 0.03603272 |
| 16821-M-06 | V-ARV-SD26 | 0.03603272 |
| LY383 | D5 | 0.03603643 |
| V-ARV-SD26 | D5 | 0.03603643 |
| K1600657 | ARV 127720 | 0.03605872 |
| K1600657 | D1 | 0.03605872 |
| K1600657 | D3 | 0.03605872 |
| LY383 | ARV 94594 | 0.03606007 |
| V-ARV-SD26 | ARV 94594 | 0.03606007 |
| AHZJ19 | T1502036 | 0.03607684 |
| 924-Bi-05 | LY383 | 0.03620248 |
| 924-Bi-05 | V-ARV-SD26 | 0.03620248 |
| AHZJ19 | K1502030 | 0.03622086 |
| D4 | ARV 94594 | 0.03804994 |
| D2 | C98 | 0.03805280 |
| D1 | D7 | 0.03805358 |
| D3 | D7 | 0.03805358 |
| D2 | S1133 | 0.03806191 |
| D2 | GX110058 | 0.03806223 |
| Reo/PA/Layer/01224A/14 | 3211-V-02 | 0.03807022 |
| D2 | GuangxiR2 | 0.03808701 |
| 17227-M-10 | ARV 126695 | 0.03812161 |
| D1 | ARV 126484 | 0.03812803 |
| D3 | ARV 126484 | 0.03812803 |
| D1 | ARV 126695 | 0.03813796 |
| D3 | ARV 126695 | 0.03813796 |
| **Alabama** | **GuangxiR2** | **0.03814742** |
| ARV 106761 | D7 | 0.03814866 |

The strains displaying the closest evolutionary distance to our isolates, are shown in bold red. The evolutionary distance is shown up to d = 0.03814866.

**Table S8. Concordance metrics for genotype-based clustering methods**

| **Method** | **ARI** | **NMI** | **Cramer’s V (bc)** | **χ² *p*** |
| --- | --- | --- | --- | --- |
| λC+σB | 0.060 | 0.193 | 0.168 | 0.286 |
| μB | 0.031 | 0.240 | 0.229 | 0.228 |
| μB+σB | 0.031 | 0.240 | 0.229 | 0.228 |
| λC+μB | 0.025 | 0.291 | 0.245 | 0.212 |
| λC+μB+σB | 0.025 | 0.291 | 0.245 | 0.212 |
| σC+λC+μB | -0.033 | 0.204 | 0.000 | 0.411 |
| σC+μB+σB | -0.033 | 0.204 | 0.000 | 0.411 |
| σC+λC+μB+σB | -0.033 | 0.204 | 0.000 | 0.411 |
| σB | -0.051 | 0.126 | 0.000 | 0.609 |
| σC+μB | -0.082 | 0.082 | 0.000 | 0.763 |
| σC | -0.083 | 0.126 | 0.000 | 0.633 |
| σC+λC | -0.083 | 0.126 | 0.000 | 0.633 |
| σC+σB | -0.083 | 0.126 | 0.000 | 0.633 |
| σC+λC+σB | -0.083 | 0.126 | 0.000 | 0.633 |
| λC | -0.091 | 0.212 | 0.000 | 0.457 |

Adjusted Rand Index (ARI), Normalized Mutual Information (NMI), bias-corrected Cramér’s V, and χ² test *p*-values are shown for each classification method (based on patristic distances among study strains) compared against three-level virulence phenotypes (Low, Moderate, High).
